# Ethnobotany, phytochemistry, and biological activities of *Psidium guajava* in the treatment of diarrhea: a review

**DOI:** 10.3389/fphar.2024.1459066

**Published:** 2024-08-23

**Authors:** Chengmei Liu, Valérie Jullian, François Chassagne

**Affiliations:** UMR 152 PharmaDev, Institut de Recherche pour le Développement (IRD), Université Paul Sabatier (UPS), Toulouse, France

**Keywords:** medicinal plants, guava, tropical countries, dysentery, diarrhea, literature review, Psidium guajava

## Abstract

Psidium guajava: is a tropical tree that is widely used in traditional medicine, especially for treating diarrhea. While *P*. *guajava* has been the subject of numerous reviews, none have specifically examined its ethnobotany, pharmacology, and phytochemistry in relation to its antidiarrheal activity. This review aims to summarize the evidence of effectiveness and safety of *P*. *guajava* in the treatment of diarrhea. Literature searches were conducted through Web of Science, PubMed, and ScienceDirect by using keywords “*Psidium guajava*” and “diarrhea” in October 2022. A total of 189 studies were included in this review. *P*. *guajava* is widely used in traditional medicine in 44 countries. Decoction and oral were the most represented method of preparation and administration, respectively, while leaves represented the most frequently cited part of the plant. Around 27 antidiarrheal or antibacterial compounds have been isolated and identified, including benzophenone glycosides, terpenes, polysaccharides, phenols, and flavonoids. This article presents ethnobotanical and pharmacological evidence for the efficacy of *P*. *guajava* leaves in the treatment of diarrhea and provides reference information for further investigation of this plant. However, despite the large number of publications on the topic, there are still some questions to answer: are quercetin and its glycosides the only ones to act as antidiarrheal agents? What is the mechanism of action of *P*. *guajava* antidiarrheal compounds? are the use of guava leaves safe in all types of populations including children, and at what dosage? To answer these questions, more complete phytochemical studies and systematic clinical trials are needed.

## 1 Introduction

Diarrhea is defined as the production of abnormally loose consistency or watery stools, usually correlated with an increased number of defecations and an excessive volume of stool output occurring within 24 h (Alam and Ashraf, 2003). According to the World Health Organization (WHO), there are around 1.7 billion cases of childhood diarrheal diseases every year, and nearly 525,000 children under the age of five die each year from diarrhea due to dehydration (WHO, 2017). Globally, diarrheal diseases stand as the second leading cause of morbidity and mortality in children younger than 5 years (Ahs et al., 2010).

Diarrheal disorders can further be divided into acute and chronic. Acute diarrhea is defined as an increased frequency of defecation lasting no more than 14 days, while chronic diarrhea lasts more than 14 days. Generally, chronic diarrhea can arise from the consumption of salts or polysaccharides that the body struggles to absorb, or from a deficiency of enzymes in the intestinal mucosa (Baldi et al., 2009). In the case of acute infectious diarrhea, the predominant cause is the ingestion of water and/or food contaminated with microorganisms. Among these microbes are bacterial agents such as *Shigella*, *Salmonella*, enterotoxigenic *Escherichia coli*, and *Staphylococcus aureus*; viral pathogens including rotavirus, coronavirus, and astrovirus; as well as protozoal organisms like *Giardia lamblia* and *Entamoeba histolytica* (Aranda-Michel and Giannella, 1999). To manage acute diarrhea, oral rehydration salts (ORS) represent a safe and effective intervention. The administration of ORS has been demonstrated to decrease the mortality, even though the stool volume and diarrhea duration are not reduced (Atia and Buchman, 2009). To prevent and treat acute diarrhea, herbal products are also used, especially in traditional medicine from tropical countries.

*Psidium guajava* L., is a tree that can grow up to 12 m and features a smooth and brown bark. The leaves are green, elliptical to oval in shape, with the top of the leaves being more acute than at the base. This tropical tree belongs to the Myrtaceae family and is commonly known as guava in English, *guayabo* in Spanish, *goyavier* in French, *guavenbaum*, *guayave* in German, and *goiaba* in Portuguese (Killion, 2000). Recent work suggest that *P. guajava* originate from Brazilian Amazonia (Arévalo-Marín et al., 2024). It is now distributed in tropical and subtropical regions worldwide (Cázares-Sánchez et al., 2010).

Decoctions and infusions made from guava leaves and bark have been traditionally used to treat diarrhea and stomachache in American countries, Africa, and Asia (Gutiérrez et al., 2008; Takeda et al., 2023). Some studies have showed that *P*. *guajava* extracts had inhibitory effects on diarrhea model both *in vivo* and *in vitro* (Vieira et al., 2001; Lu et al., 2020). In recent years, researchers have focused their investigations on elucidating the chemical composition, pharmacological activities, and clinical effects associated with *P*. *guajava*. The compounds present in *P*. *guajava* can be divided into different chemical classes, including flavonoids, terpenoids, polyphenols, alkaloids and glycosides (Bhagavathy et al., 2019). It is also reported that *P*. *guajava* possesses pharmacological effects, such as anti-inflammatory and analgesic (Ojewole, 2006), antibacterial, antioxidant, and antitumor activities (Braga et al., 2014).

To date, many reviews focusing on the traditional uses, phytochemistry and pharmacology of *P*. *guajava* have been published (Gutiérrez et al., 2008; Díaz-de-Cerio et al., 2017; Naseer et al., 2018; Amadike Ugbogu et al., 2022; Takeda et al., 2023). However, no comprehensive review on the use of *P*. *guajava* for the treatment of diarrhea and its associated toxicity and safety effects has been performed yet. To address this gap, the objective of this review is to provide an overview of ethnobotanical, pharmacological, and phytochemical studies on *P*. *guajava*, specifically focusing on its effects on diarrhea.

## 2 Materials and methods

### 2.1 Literature search strategy

A systematic literature review was conducted by searching three scientific electronic databases: Web of Science, PubMed, and ScienceDirect. We used two keywords “*P. guajava*” and “diarrhea” to perform this review. Only results found before 12 October 2022 (with no limit of time before this date) were included in this study. PRISMA guidelines were followed to perform this review (Page et al., 2021).

### 2.2 Eligibility criteria

The inclusion criteria were as follows: 1) ethnobotanical studies focusing on *P*. *guajava* used to treat diarrhea/dysentery; 2) pharmacological assays of *P*. *guajava* extracts related to diarrheal disorders (i.e., *in vitro* antibacterial activities, *in vivo* antidiarrheal effects, *in vitro* and *in vivo* anti-inflammatory properties, anti-parasitic activities, *in vivo* spasmolytic effects, *in vivo* and *ex vivo* intestinal motility effects, *in vitro* antiviral activities); 3) phytochemical data from *P*. *guajava* focusing on compounds related to diarrhea; 4) toxicological studies of *P*. *guajava*; (5) clinical trials of *P*. *guajava* employed in patients with diarrheal disorders.

Studies that have one of the following criteria were excluded: 1) review type articles; 2) studies published in poor quality journal (2-year impact factor below 1.0); 3) no ethnobotanical data on diarrhea; 4) other types of articles: conference, content, dictionary, index; 5) studies that investigated other diseases; 6) studies that referred to other plant species; 7) phytochemistry not linked to diarrhea; 8) other biological assays used (i.e., anticholinesterase, antifungal, anti-oxidant, antiviral, hepatotoxicity, insecticidal, mosquitocidal, other antibacterial assays); 9) languages other than English.

### 2.3 Data extraction

Results obtained from electronic databases were uploaded to the online application: Rayyan (Ouzzani et al., 2016). Screening of titles, abstracts and full texts was performed by two independent reviewers based on the inclusion and exclusion criteria. Labels (i.e., clinical, ethnobotany, pharmacology, phytochemistry, toxicity, and veterinary) for each included study were added and reasons for the exclusion of ineligible studies were recorded. Disagreements between the two reviewers were resolved through discussion (involving a third person if needed). Information extracted included publication type, title, author names, abstract, publication name, publication date and labels.

## 3 Results

### 3.1 Description of studies

**FIGURE 1:**
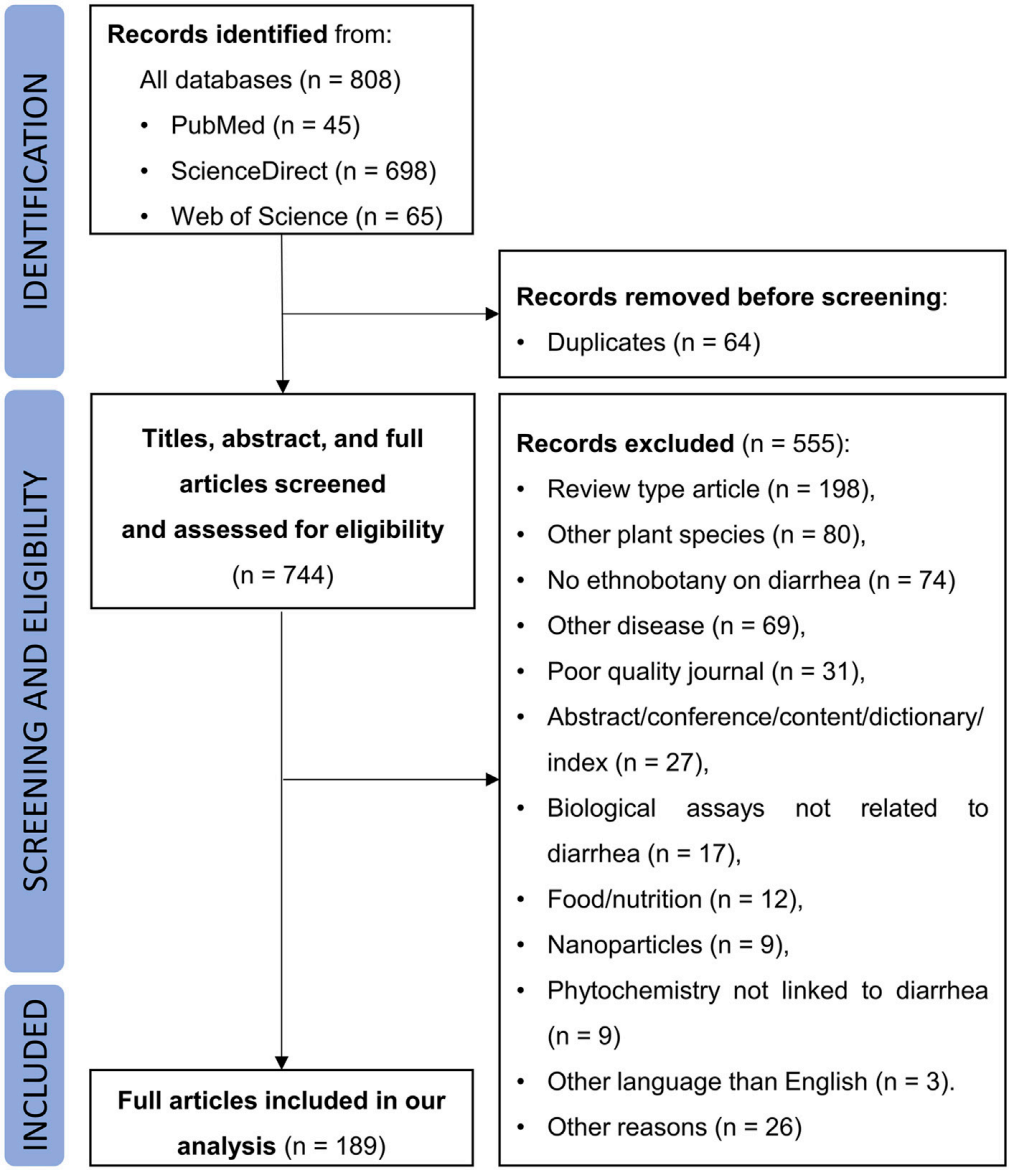
Screening flowchart of the study.

A total of 808 records were identified from the literature search, among which 64 were duplicates. The remaining 744 studies were screened by titles, abstracts and full-texts. According to the inclusion and exclusion criteria, 555 records were excluded for the following reasons: review type article (n = 198), other plant species (n = 80), no ethnobotany on diarrhea (n = 74), other disease (n = 69), poor quality journal (n = 31), abstract/conference/content/dictionary/index (n = 27), biological assays not related to diarrhea (n = 17), food/nutrition (n = 12), nanoparticles (n = 9), phytochemistry not linked to diarrhea (n = 9), other language than English (n = 3), other reasons (n = 26). Finally, 189 studies were included in this review (Figure 1).

### 3.2 Ethnobotany

**FIGURE 2:**
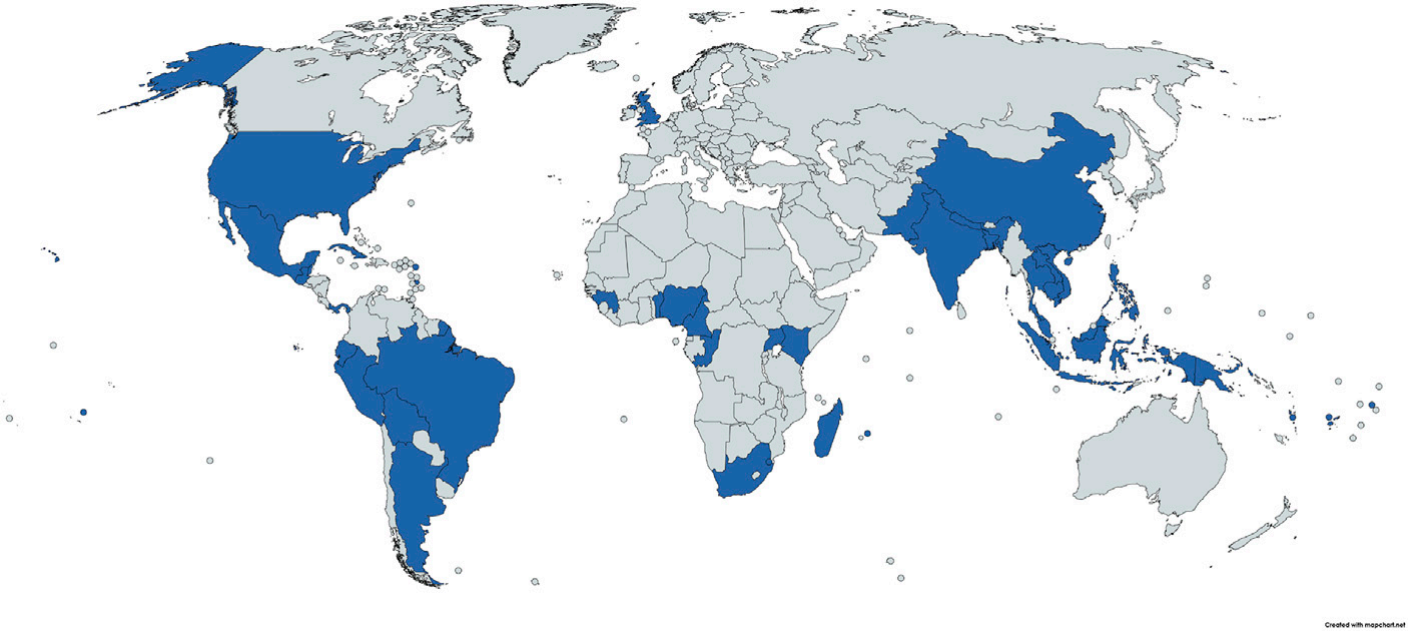
Map showing (in blue) the countries where *Psidium guajava* is used for treating diarrhea (created with mapchart.net).

Among the 189 studies included, 121 reported ethnobotanical data on *P. guajava* to treat diarrheal disorders and 6 reported ethnoveterinary knowledge of *P*. *guajava* on livestock diarrhea. These surveys were conducted in a total of 44 different countries, of which India ranked first (18 publications, 14.2%), followed by Brazil (13, 10.2%), Mexico (12, 9.4%), South Africa (8, 6.3%), Thailand (7, 5.5%), Peru (5, 3.9%), China (4, 3.1%), Indonesia (4, 3.1%), Benin (4, 3.1%), Congo (3, 2.4%), Ecuador (3, 2.4%), Malaysia (3, 2.4%), Mauritius (3, 2.4%), Uganda (3, 2.4%), Kenya (3, 2.4%), Argentina (2, 1.6%), Bolivia (2, 1.6%), Cameroon (2, 1,6%), Trinidad and Tobago (2, 1.6%), and others countries (26 in total and 1 study for each, 0.8%) including Bangladesh, Belize, Cambodia, Cuba, Fiji, French Guiana, French Polynesia, Guadeloupe, Guatemala, Guinea, Laos, Madagascar, Martinique, Nepal, Nigeria, Pakistan, Palestine, Panama, Papua New Guinea, Philippines, Samoa, Eswatini, United Kingdom, United States, Vanuatu and Vietnam (Figure 2). Overall, *P*. *guajava* is used as an antidiarrheal plant in countries across the Americas, Asia, Africa and Oceania.

Of the 127 publications reporting ethnobotanical and ethnoveterinary data, 122 were based on field surveys and 5 on historical reports (Supplementary Table S1).

**FIGURE 3:**
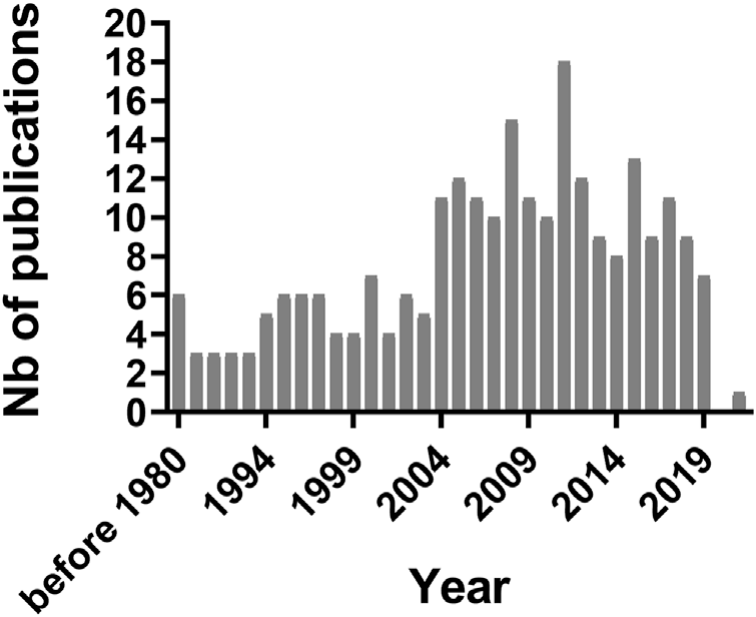
Study period and number of publications of ethnobotanical field surveys.

Ethnobotanical field surveys were performed from 1892 to 2021, and the most represented period was from 2004 to 2018 (Figure 3). In these ethnobotanical surveys, different informants were interviewed including inhabitants (i.e., breeders, elders, farmers, homegarden owners, householders, mothers, women, and those who have knowledge of medicinal plants), traditional practitioners (i.e., dukuns, local collectors, herbalists, indigenous, midwives, shamans, and traders), medical staff (nurses, dentists, pharmacists, physicians), and patients. Of the 122 publications presenting ethnobotanical field surveys, 67 focused on traditional knowledge obtained from inhabitants, 22 focused on the knowledge from traditional practitioners, 23 focused on knowledge from both inhabitants and traditional practitioners, one focused on medical staff, and two targeted patients. Seven publications did not document this information. The informant sample sizes ranging from 7 to 1,614.

**FIGURE 4:**
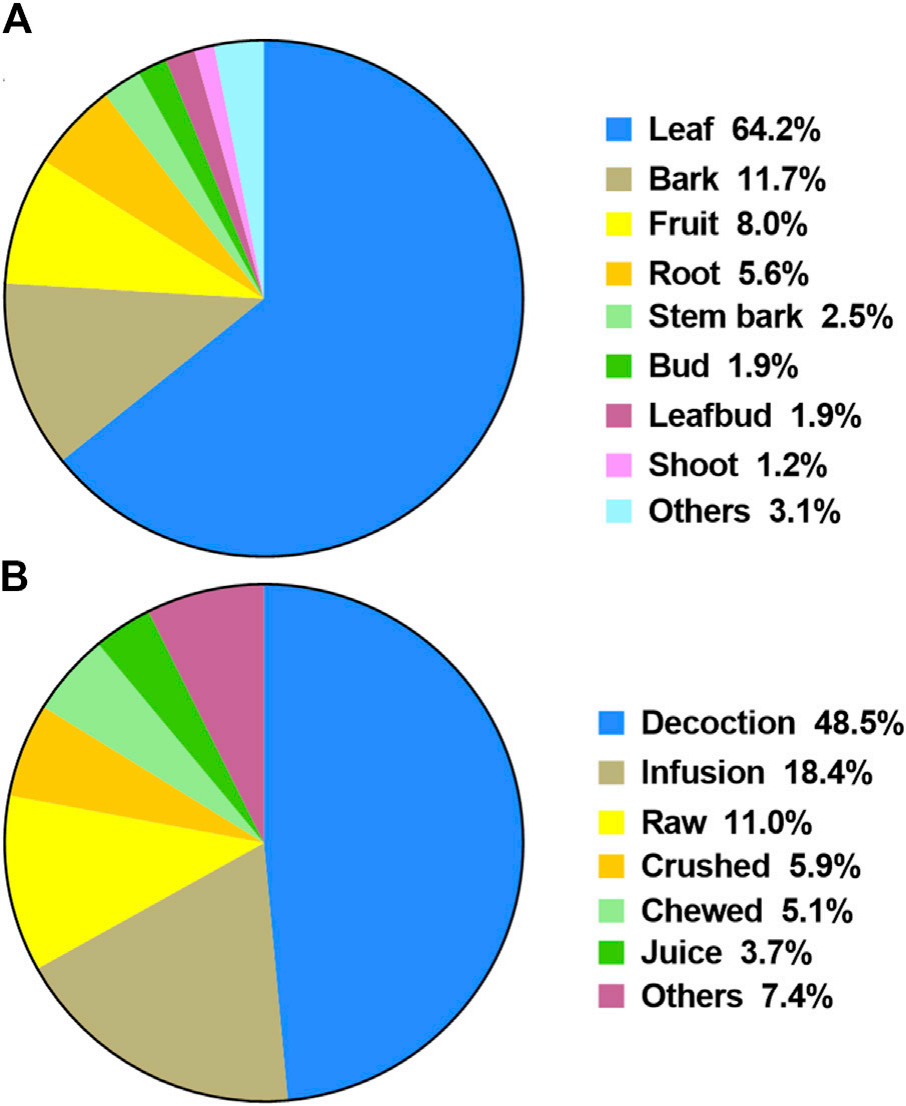
**(A)** Part of plants used by people to treat diarrhea. (Other parts include apex of branch, flower, fruit bud, sprout and wood); **(B)** Method of preparation of plants used by people to treat diarrhea. (Other methods include bark pasted, cataplasm, cooked, fresh, grilled on fire, grinded, heated, maceration, socked and squeeze).

The most frequently used plant parts were leaves (110 citations, 64.3%), followed by bark (19 cit., 11.1%), fruit (13 cit., 7.6%), root (9 cit., 5.3%), and stem bark (4 cit., 2.3%) (Figure 4).

Decoction was the main method of preparation used (70 citations, 49.3%), followed by infusion (26 cit., 18.3%), raw preparation (i.e., no preparation) (15 cit., 10.6%), crushed (8 cit., 5.6%), and chewed (7 cit., 4.9%). A total of 110 publications showed that the preparations were made from *P*. *guajava* only, while 17 publications reported that *P*. *guajava* were mixed with other plants (i.e., *Albizia adianthifolia* (Schumach.) W.Wight, *Anacardium occidentale* L., *Ananas comosus* (L.) Merr., *Bombax ceiba* L., *Coffea arabica* L., *Glochidion* sp., *Justicia gendarussa* Burm.f., *Lippia alba* (Mill.) N.E.Br. ex Britton & P.Wilson, *Mangifera indica* L., *Myrtus communis* L., *Newbouldia laevis* (P.Beauv.) Seem., *Nypa fruticans* Wurmb, *Punica granatum* L., *Ruta graveolens* L., *Spondias mombin* L., *Spondias purpurea* L.).

Oral was the most represented method of administration (87 citations, 55.8%), followed by bath (1 cit., 0.6%) and local application (1 cit., 0.6%). Sixty-seven publications (42.9%) did not document this information.

### 3.3 Pharmacology

Out of the 189 articles, 53 discussed the pharmacological properties of *P*. *guajava* extracts, including their antimicrobial activity against various pathogenic microorganisms that cause diarrhea, their antidiarrheal activity in different animal models, their anti-inflammatory properties associated with diarrhea, their effectiveness against parasites linked to diarrhea, and antiviral potential. Eleven articles reported both phytochemistry and pharmacology data. In this section, we decided to focus only on the pharmacological part of this paper, and the phytochemical part will be shown in the phytochemical section. A total of 27 studies reported the antibacterial activity of *P*. *guajava* extracts, and 17 articles investigated the antidiarrheal activity *in vivo* or *ex vivo* using animal models, 10 publications dealt with anti-inflammatory properties, six articles reported antiparasitic activity, and three showed antiviral activity. Out of the 53 articles, 48 studies focused on the inhibitory effects of leaf extracts. The preparations used included decoction, maceration, soaking, infusion, ultrasound, and crushing. A summary of the findings from these studies is presented below and more details are provided in Supplementary Table S2.

#### 3.3.1 Antibacterial activity

Diarrhea is caused by a variety of infectious agents, including bacteria, protozoa, and viruses. Among the bacterial agents are included species of *Shigella*, *Salmonella*, *E. coli*, *Yersinia*, and *Campylobacter* (Aranda-Michel and Giannella, 1999; Soltan-Dallal, 2001). Antibacterial methods are classified into two main groups: diffusion on a solid nutritive medium (agar disk and agar well diffusion method) and dilution in a liquid broth (broth dilution method). Activity can be detected by observing the growth of micro-organisms (Cos et al., 2006).

In this context, we conducted a systematic analysis of publications exploring the antibacterial activity of *P*. *guajava* concerning its relevance to diarrhea. The categorization is performed according to the testing methods employed.

##### 3.3.1.1 Agar disk diffusion method

In the agar disk diffusion method, filter paper disks (about 6 mm diameter) infused with a known concentration of the test compounds or extracts are placed on the surface of the inoculated agar. After incubation, the test substances diffuse from the disks into the agar, and so inhibit the growth of microorganisms, resulting in a visible zone of inhibition. Subsequently, the diameter of the inhibition zone is measured using a ruler. Researchers employed this method as initial screening techniques in microbiological studies, because of its simplicity, low cost, and the ease to interpret results. However, this method presents some disadvantages such as the low diffusion of non-polar compounds leading to false negative results, and the impossibility to determine the minimum inhibitory concentration (MIC) (Balouiri et al., 2016).

The n-hexane, acetone and methanolic extracts of *P*. *guajava* leaves showed activities against enteropathogenic *E*. *coli*, *Salmonella* Typhi, and *Shigella flexneri* at 50 mg/disk, with the bacterial inhibition zone ranging from 6 to 14 mm (Cáceres et al., 1993). The methanol and acetone extracts gave larger inhibition zones than n-hexane extract, while acetone extract presented the largest inhibition zone of 14 mm against *S*. *flexneri*. In another study, the methanolic extract of *P*. *guajava* leaves showed significant inhibitory activities against two isolates of *E*. *coli*, two isolates of *Salmonella* spp., and various strains of *Shigella* spp. (including *Shigella dysenteriae*, *S*. *flexneri* and *Shigella virchow*), exhibiting an inhibitory zone of more than 10 mm (at a dose of 10 mg/disk), while the standard antibiotics (nalidixic acid, tetracycline, chloramphenicol) showed activity at 30 µg/disk (Lin et al., 1999; 2002). Given the utilization of high concentrations in these two studies, we can assume that the antibacterial effect is not clear. Moreover, the antibacterial zones of the positive controls were not provided in the last publication.

The antibacterial effects of hexane, ethyl acetate and methanol extracts of *P*. *guajava* fruits were also examined using agar disk diffusion method, but no activity was found against *Bacillus subtilis*, *E*. *coli*, *Pseudomonas aeruginosa* and *S. aureus* at 500 µg/disk compared to the gentamicin at 10 µg/disk (McCook-Russell et al., 2012).

##### 3.3.1.2 Agar well diffusion method

In the agar well diffusion method, wells are created in the agar with 6–8 mm diameter, and different test substances, such as antimicrobial agents or extracts, are introduced into these wells. The substances diffuse into the agar, and inhibit the growth of the microorganisms. The antimicrobial effects are then assessed by measuring the zones of inhibition around the wells (Balouiri et al., 2016). This method presents the same advantages and disadvantages to the agar disk diffusion method and is mainly suitable for a preliminary screening.

Preliminary antimicrobial activity of guava leaves aqueous extract was investigated by agar well diffusion method (Adamu et al., 2005). Wells, with 8 mm diameter, were punched into the agar medium, and these wells were filled with the extract at a concentration of 200 mg/mL. Gentamicin (at concentration of 100 mg/mL) was used as a positive control. The results indicated a weak antibacterial inhibition against *Proteus mirabilis* with an inhibition zone diameter around 15 mm. But no effect was found for *E*. *coli*, *P*. *aeruginosa* and *S*. *aureus*. The concentrations used in this work are high and therefore the results should be considered with caution. To investigate the antimicrobial potential of *P*. *guajava* leaves, chloroform, ethanol and aqueous extracts were prepared, and agar-well diffusion test (wells with 6 mm diameter) and broth microdilution methods were used (Pesewu et al., 2008). These extracts exhibited inhibitory effects against a spectrum of tested bacterial strains, including methicillin-resistant *S. aureus* (MRSA), methicillin-sensitive *S. aureus* (MSSA), *E*. *coli*, *P*. *aeruginosa*, *Proteus vulgaris* and *Streptococcus pyogenes*. The observed inhibition zones ranged from 8 to 25 mm for 5 mg of extract (100 µL of 50 mg/mL solutions). As a plant antimicrobial control, aqueous extract of allicin at the same dose was employed, showing inhibition zones ranging from 10 to 35 mm. Additionally, an extract obtained with a Stomacher^®^ blender showed an anti-MSSA effect with a MIC value of 3.1 mg/mL. Once again, antimicrobial effect can be detected at high dose only. The decoction and hydro-ethanolic extract of *P*. *guajava* leaves showed antibacterial activities towards *Vibrio cholerae* at a dose of 100 µL of extract (no more details were indicated for the concentration) (Shittu et al., 2016). In this study, the absence of concentration details for the extracts does not allow for the precise determination of the antibacterial effect.

The aqueous and 70% acetone extracts of *P*. *guajava* leaves showed similar antibacterial activity against *S*. *aureus* and *S*. *flexneri* with an inhibition zone higher than 17 mm in agar well diffusion assay (6 mm diameter) for 5 mg of extract (80 µL of a 62.5 mg/mL solution) (de Araújo et al., 2014). The same extract showed no or little activity against *E*. *coli* and *Salmonella* Enteritidis while the inhibition zones of positive group chloramphenicol were around 22 and 34 mm at 30 µg/disk. Acetone extract showed a highest activity against *Staphylococcus epidermidis* with MIC value of 39 μg/mL. Leaf extracts of *P*. *guajava* were prepared using ethanol and hot distilled water and the antibacterial effects of the extracts on *Salmonella* species and *Shigella* species (isolated from two fecal contamination wells) were determined using the agar well diffusion method (6 mm diameter) (Sule et al., 2022). Results revealed that ethanol extract produced an inhibition zone of 22.0 mm at a dose of 20 mg/mL against *Shigella*, 17 mm at 100 mg/mL, 10.3 mm at 50 mg/mL, and a zone of inhibition of 6.7 mm at a dose of 2.5 mg/mL, but no zone of inhibition was observed on *Salmonella* species and aqueous extract did not show any antibacterial activities. An aqueous extract made from guava leaves was screened for its anti-*Bacillus anthracis* activity using agar well diffusion assay and broth microdilution methods, but guava leaf extract did not show any activity under the assay conditions used (Kaur et al., 2021).

Because the diffusion method is neither suitable for evaluating non-polar samples nor those that do not easily diffuse into agar, nor for quantifying antimicrobial activity, another method is often employed as a reference, that is the broth microdilution method.

##### 3.3.1.3 Broth microdilution method

The dilution method is a laboratory technique commonly used to determine the MIC of an antimicrobial agent against a particular microorganism. MIC value is the lowest concentration of the antimicrobial agent that produces a complete suppression of colony growth (Balouiri et al., 2016), which can be detected by measuring the optical density. Two-fold serial dilutions of test samples are made in medium to obtain the desired final concentrations. The inoculum size, the type of growth medium, and the incubation time influence MIC values. Therefore, it is important to standardize the parameters, with bacterial inoculum (1 × 10^5^ CFU/mL ≤ inoculum ≤1 × 10^7^ CFU/mL equivalent to 0.5 McFarland (1.5 × 10^8^) with dilution from 1:15 to 1:300) and time of incubation (less than 24 h, except *Mycobacterium* sp. and *Helicobacter pylori*) (Chassagne et al., 2021).

The effect of *P*. *guajava* leaf extract was studied for its antibacterial activity by broth microdilution method. The study utilized a standardized inoculum of 10^5^ CFU/mL and the extract showed inhibitory activity against *S*. *aureus* with MIC value of 256 μg/mL after 24 h of incubating (Morais-Braga et al., 2016). This result supports the use of *P*. *guajava* for modulating bacterial growth. In another study, *P*. *guajava* flavonoid mixtures, extracted from leaves, flesh and peel, were tested against *E*. *coli, S*. *aureus, Salmonella enterica and S*. *flexneri* (Zhang et al., 2018). The inoculum was adjusted to 10^6^ CFU/mL and penicillin used as positive control. The best activity was observed for leaves and peel flavonoid inhibiting the growth of *S*. *aureus* with MIC value of 0.3 mg/mL after incubating at 37°C for 24 h. Leaf, peel and fruit flavonoids showed MIC values against tested strains between 0.3 and 5 mg/mL.

In a study evaluating *P*. *guajava* from Peru, the MIC of an ethanolic extract of *P*. *guajava* leaves and stem against *S*. *aureus* was determined as 16 mg/mL (Bussmann et al., 2010). Considering that an MIC value above 625 μg/mL can be interpreted as a weak antibacterial effect (Aro et al., 2019), the MIC value obtained for the Peruvian guava is too high to demonstrate an antibacterial effect. A decoction of guava leaves showed antibacterial activities against *Citrobacter diversus*, *E*. *coli*, *Escherichia paracoli*, *Klebsiella pneumoniae*, *P*. *aeruginosa*, *S*. *aureus*, *Salmonella* Enteritidis and *S*. *flexneri* with MIC ranging from 15.6 to 62.5 μg/mL, while the decoction of stem bark inhibited the growth of tested bacteria with MIC values of 125 or 250 μg/mL (Tona et al., 1999). In this study, the investigation did not establish standardization for the bacterial inoculum. The bacterial suspension was prepared by transferring colonies directly into a small volume of 0.9% saline, and 5 mL of this suspension was added to 100 mL of Mueller-Hinton medium. Consequently, results about the antibacterial activities of the decoction should be considered with caution.

Acetone extract of *P*. *guajava* leaves showed antibacterial activities towards *E*. *coli*, *Enterococcus faecalis*, *S*. *aureus*, *Salmonella* Isangi, *Salmonella* Typhimurium, *S*. *flexneri*, *Shigella sonnei* with MIC ranging from 0.156 to 0.312 mg/mL, while the MIC of gentamicin ranged from 0.039 to 2.5 mg/mL (Bisi-Johnson et al., 2017). Ethanolic guava leaf extract with and without chlorophyll removal showed antibacterial properties against *E*. *coli*, *Listeria monocytogenes*, *P*. *aeruginosa*, *S*. *aureus* and *Vibrio parahaemolyticus* with an MIC value ranging from 32 to 128 μg/mL (Olatunde et al., 2019; 2021). This study used a final bacterial concentration of 10^6^ CFU/mL and a time of incubation of 24 h at 37°C. Aqueous extracts of *P*. *guajava* leaves obtained by maceration, infusion and decoction were tested against enteropathogenic *E*. *coli* (EPEC), *S*. *enterica* and *Clostridium perfringens* (with 10^6^ CFU/mL bacterial suspension) at 37°C for 24 h (Chouegouong et al., 2023). The infused and macerated extracts showed inhibition against three bacteria with MIC values of 1.25 mg/mL. The decoction extract showed antibacterial activities against *E*. *coli* EPEC (MIC = 2.5 mg/mL), *S*. *enterica* (MIC = 2.5 mg/mL) and *C*. *perfringens* (MIC = 1.25 mg/mL). Oxytetracycline 20% was used as the positive control, and inhibited the growth at a dose of 0.1 µg/mL. The aqueous and ethyl acetate fractions showed antibacterial activities against *E*. *coli*, *S*. *aureus* and *S*. Typhimurium with MIC values ranging from 1.25 to 7.5 mg/mL (Hall et al., 2023). This study was conducted following the criteria: inoculum of 10^5^ CFU/mL and time of incubation of 24 h at 37°C. Notably, these results demonstrate the potential of *P*. *guajava* as an effective antibacterial agent.

##### 3.3.1.4 Agar disk diffusion and broth microdilution methods

Ethanolic and aqueous extracts of *P*. *guajava* leaves were subjected to *in vitro* antibacterial test by agar disk diffusion (filter paper discs at 6 mm) and broth microdilution methods (bacterial inoculum at 10^4^ CFU/mL) (Voravuthikunchai et al., 2004). The extracts produced inhibition zones against 12 different strains of *E*. *coli* with inhibition zones ranging from 7 to 8 mm at a concentration of 2.5 mg/disk. According to the agar disk screening, both aqueous and ethanolic extracts were further studied to determine the MIC. With broth dilution method, aqueous extract (MIC = 0.19 or 0.78 mg/mL) was more active than ethanolic extract (MIC = 6.25 or 12.5 mg/mL). Amikacin, ampicillin, gentamicin, kanamycin, and tetracyline (10–30 µg) were used as controls, but the antibacterial data for the controls were not clearly explained. In another study, different organic solvents (chloroform, ethyl acetate, n-hexane, methanol) were used to extract *P*. *guajava* leaves (Afzal et al., 2019). The preliminary results showed that the methanolic extract possessed the highest inhibitory potential against *B*. *subtilis*, *E*. *coli*, and especially against *S*. *aureus* (inhibition zone ranging from 21 to 25.6 mm). However, the quantity of extract in the tested agar disks remained ambiguous due to the specified application of “1 µL/1 mL” of extract suspension per paper wick. Furthermore, the precise concentration of the suspension was not detailed. In this study, the leaf extracts exhibited significant antibacterial effect at high concentration. The chloroform extract exhibited more sensitivity to the growth of *S*. *aureus* with an MIC of 98 ± 4.1 μg/mL. The n-hexane extract showed an effect against *B*. *subtilis* (112 ± 5.2), *E*. *coli* (111 ± 1.1) and *S*. *aureus* (167 ± 6.1) μg/mL. Ethyl acetate extract inhibited the growth of *B*. *subtilis*, *E*. *coli* and *S*. *aureus* with MIC ranging from 108 ± 2 to 237 ± 5.1 μg/mL. The methanolic extract demonstrated an inhibitory effect against *B*. *subtilis*, *E*. *coli* and *S*. *aureus* with an MIC of 115 ± 4.2, 192 ± 2.1 and 233 ± 5.5 μg/mL, respectively. Another study showed that the ethanolic extract of *P*. *guajava* leaves inhibit *S*. *aureus, E coli, Bacillus cereus, Salmonella* Enteridis growth with inhibition zone diameters between 10 and 15 mm at 50 mg/disk (Hemeg et al., 2020). The MIC of this extract against *E*. *coli*, *S*. *aureus* and *S*. Enteritidis were 625, 1,250 and 625 μg/mL, respectively.

##### 3.3.1.5 Others

In this section, we summarize the results of other publications assessing the antibacterial activity by using different methods and alternative approaches.

The ethanolic extract of *P*. *guajava* leaves was examined on the growth of 11 bacteria (including *S*. *dysenteriae*, *S*. *flexneri*, *Shigella boydii*, *S*. *sonnei* and *S*. Typhi) using the *in vitro* agar dilution streak method (Maïkere-Faniyo et al., 1989). Specifically, the extract was added to the agar medium to get a final concentration of 1,000 μg/mL and stored for 6 h at 37°C before use. The bacteria were then inoculated radially on the agar medium containing the extract and incubated at 37°C for 24 h. The results revealed an inhibitory effect toward one species of *S*. Typhi, two species of *S*. *flexneri* and *S*. *dysenteriae*. Ampicillin was used as a control. Water, four diluted acetone and four diluted ethanol (20%, 50%, 60% and 80% solvent in water) were used to prepare extracts from guava leaves (Vieira et al., 2001). These extracts were tested on bacteria by two methods: radial diffusion in two layers of perforated agar (RDAP), and agar disk diffusion method. The RDAP method contains two layers of agar, the bottom layer contains the test microorganism, and the top layer (perforated with holes or wells) serves as a medium for the diffusion of the extracts being tested. All concentrations of guava leaf extracts showed inhibitory effects on the growth of *E*. *coli* and *S*. *aureus* strains (halos ranging from 15 to 32 mm). The aqueous, the 60% acetone and 60% ethanol extracts effectively inhibited the growth of *S*. *aureus* and *E*. *coli* with halos ranging from 26 to 32 mm diameter. A similar pattern of activity was obtained for the same extracts with the agar disk diffusion method (with inhibited zone diameter between 13 and 20 mm).

Lectins can be employed to investigate how pathogens recognize oligosaccharide structures on the cell surface. They have the capability to bind to specific sugars in glycoconjugates of *E*. *coli*, inhibiting its adhesion to the intestinal wall and consequently preventing infection that may lead to diarrhea. The extract from guava fruits (macerated with phosphate buffer solution pH 7) were used to detect lectin activity by assessing its hemagglutination (HA) capacity. Results showed that the extract exhibited hemagglutinating activity as it prevented adhesion of *E*. *coli* 0157:H7 to red blood cells, which demonstrated the presence of lectins. In the following study, we present an antibacterial effect mediated by light as some natural compounds exhibit toxic effects on organisms and living cells when they are excited by light (Towers et al., 1997). The ethanolic extracts of *P*. *guajava* leaves and fruits were tested for their light-mediated activities. The extracts showed activities against *B*. *subtilis*, *E*. *coli*, *P*. *aeruginosa* and *S*. *aureus* under ultraviolet (UV) light with a zone of inhibition between 8 and 12 mm for 2 mg (20 μL, 100 mg/mL) (Lopez et al., 2001; Cheeptham and Towers, 2002). An acid resistance test was performed, which exposed cells to *P. guajava* extract and then subjecting them to acidified media (pH 3.8) (Lim et al., 2013). The results showed that ethanolic extract of guava fruits had greater effect in decreasing the acid resistance of *S*. Typhimurium (10^7^ CFU/mL, incubated at 37°C for 24 h) at a dose of 50 mg/mL.

Fractional inhibitory concentration (FIC) index is used to calculate synergy, indifference, or antagonism between two compounds, where <0.5 indicates synergy, 0.5–4 indifference, and >4 antagonism (Hall et al., 1983; Hawas et al., 2022). A study was designed to test the *in vitro* antimicrobial efficacy of *P*. *guajava* leaves in combination with other plants. The dichloromethane-methanol (1:1) extract demonstrated broad-spectrum activity and this extract combined with dichloromethane-methanol (1:1) extract of *Brachylaena transvaalensis* Hutch. ex E.Phillips & Schweick. was mostly synergistic (mean fractional inhibitory concentration (ΣFIC) value of 0.4), while the decoction of *P*. *guajava* combined with dichloromethane-methanol (1:1) extract of *Acanthospermum glabratum* (DC.) Wild demonstrated a mean ΣFIC value of 0.5 (van Vuuren et al., 2015).

In another study, the antibacterial activity was determined by a microtiter plate-based assay (96-well plate), which was used to study biofilm formation and observe the adherence of bacteria to an abiotic surface (Birdi et al., 2010). The results were presented as half maximal effective concentration (EC_50_). The decoction of dried leaves of *P*. *guajava* exhibited antibacterial activity towards *S*. *flexneri* (EC_50_ value 0.3 mg/mL) and *V. cholerae* (EC_50_ value 0.8 mg/mL). Ofloxacin, an antibiotic, was used at a concentration of 1 μg/mL and completely inhibited the growth of all the bacterial strains. The decoction also showed a greater decrease in the adherence of *E*. *coli* B170 to HEp-2 cells (EC_50_ value 0.1 mg/mL), meaning that the decoction contains components that prevent the attachment of the bacterial to the cell, and inhibited the invasion of *E*. *coli* E134 and *S*. *flexneri* to Hep-2 cells, with EC_50_ value 0.06 and 0.05 mg/mL.

Overall, the studies discussed here showed that *P*. *guajava* leaves show antibacterial potential towards bacteria causing diarrhea such as *E*. *coli*, *Salmonella* spp., *Shigella* spp., *V*. *cholerae*, particularly when using methanol, acetone and ethanol as solvents. This indicates the presence of broad-spectrum antibiotic compounds in *P*. *guajava*.

#### 3.3.2 Antidiarrheal activity

##### 3.3.2.1 Gastrointestinal propulsion

Diarrhea is closely tied to gastrointestinal propulsion, which refers to the movement of ingested material through the intestinal tract (Sarna and Otterson, 2008). The intestinal tract is a long and complex system responsible for the digestion and absorption of nutrients. It consists of several organs, including mouth, esophagus, stomach, pylorus (lower part of the stomach that connects to the duodenum), small intestine (duodenum, jejunum and ileum), caecum (located between the small intestine and the large intestine), large intestine (colon), rectum and anus (Kumral and Zfass, 2018). Typically, researchers used isolated intestines to investigate propulsion and evaluate the transport of food, fluids, and waste products. In the case of diarrhea, the transit time of digestive contents through the intestines is accelerated and the absorption of water and nutrients is limited, resulting in the production of loose and watery stools. Gastrointestinal transit time is a common assay conducted with charcoal suspension and BaSO_4_ (barium sulfate) milk, and the distance traveled by the charcoal or BaSO_4_ milk is a measurement of gastrointestinal motility (Al-Saffar et al., 2019).

*P*. *guajava* leaf aqueous extract antidiarrheal effect on the transportation of water of separated intestinal parts (i.e., duodenum, jejunum, ileum and colon) was determined, and expressed as “mL” of water transferred from mucosa to serosa (results shown as negative values) or serosa to mucosa (results shown as positive values) in mice (Almeida et al., 1995). The results showed that guava extract (no details of the dose administered was provided) increased water absorption in the colon’s intestinal portion, with water flow values of −8.3 ± 4.7 μL/g/h. Additionally, on the gastrointestinal propulsion (charcoal suspension) test, mice fed with an aqueous extract solution of 0.1 mL per 10 g live weight significantly reduced gastrointestinal propulsion, as the fractional value (distances traveled by the charcoal/length of the intestine) was 0.45 ± 0.04, while the control group was 0.72 ± 0.05, with a significant difference from control (*p* < 0.05). In a second study, administration of either methanolic or aqueous *P*. *guajava* leaf extracts (400 mg/kg) for 30 min significantly slowed down the movement of charcoal meals from pylorus to the caecum (Lin et al., 2002). The gastrointestinal motility value was calculated as the percentage of total intestine length covered by the travel distance of charcoal meal. Results showed that distance covered by the charcoal meal in the experimental groups was reduced to 50%–70% of the full intestine length compared with 91%–92% in the control group. In another study, oral pretreatment with 50, 100 and 200 mg/kg of methanolic extract of *P*. *guajava* leaves on male Wistar rats significantly reduced the charcoal meal intestinal transit (from pylorus to caecum), with the percentage of charcoal meal travelled being 29.9% ± 1.4%, 25.4% ± 2.1%, 19.0% ± 1.6%, respectively, compared to negative control group (58.7% ± 1.8%) and positive control (23.4% ± 2.1% for atropine at a dose of 1 mg/kg) (Olajide et al., 1999). An inhibition of intestinal motility, evidenced by shorter distances traveled by the charcoal meal in rats, was observed in all groups administered with the leaf extract at doses of 200, 400 and 8,000 mg/kg when compared to control (*p* < 0.05), with 14.8% inhibition at 200 mg/kg, 16.9% inhibition at 400 mg/kg and 33.6% inhibition at 8,000 mg/kg (Ibeh et al., 2021).

One study showed that the normal propulsion rate in the small intestine in male Sprague-Dawley rats (200–250 g) was 75.4% ± 6.0% per hour (Lutterodt, 1992). After treating with 1.0 mL/100 g 90% microlax solution, the propulsion was 100% per hour, which was used as the reference value for diarrhea. Studies showed that an aqueous extract of guava fresh leaves at a dose of 0.2 mL/kg (no details of the concentration of the solution was provided) produced 63.6% inhibition of propulsion in the model of microlax-induced diarrhea. Morphine was used as a standard drug of reference, as morphine injected into rats decreased intestinal propulsion (Burleigh et al., 1981). This guava leaf extract exhibited a similar activity to the administration of 0.2 mg/kg of morphine sulphate (65.6% inhibition).

A gastrointestinal motility test with BaSO_4_ milk (BSM) was carried out on male Wister rats (Mazumdar et al., 2015). The distance traveled by BaSO_4_ milk was measured and expressed as a percentage of the total length of small intestine (from pylorus to the ileo-cecal junction). The ethanolic extract of *P*. *guajava* leaves (250, 500, 750 mg/kg) significantly decreased the gastrointestinal motility of rats in BSM model after 30 min with percentages of inhibition in three treated groups of 29.6%, 30% and 37.8%, respectively, while positive control group (loperamide at 2 mg/kg) exhibited 39.6% inhibition.

These studies highlight the potential of *P*. *guajava* leaf extracts as effective agents in gastrointestinal functions, particularly in diarrheal disorders. *P. guajava* acts by increasing the water absorption in the colon and by reducing gastrointestinal propulsion rates and motility. The inhibitory effects on gastrointestinal function exhibited by *P*. *guajava* extracts were comparable to those of established antidiarrheal agents such as morphine and loperamide. These findings not only validate the traditional use of guava leaves in managing gastrointestinal ailments but also provide information on potential mechanisms of action.

##### 3.3.2.2 Bacteria induced infectious diarrhea

*Citrobacter rodentium*, a mouse pathogen, is recognized for its ability to imitate the pathogenic traits observed in enteropathogenic and enterohemorrhagic *E*. *coli*. *C*. *rodentium* infected Swiss albino mice model was employed to test the efficacy of *P*. *guajava* hydro-ethanolic leaf extract in treating infectious diarrhea (Gupta and Birdi, 2015). The bacterial load in the fecal sample of mice in the test group (treated with 300 mg/kg/day *P*. *guajava* leaf extract for three consecutive days) was high on day 4, which suggested a flush out of the bacteria. The tested group showed quicker clearance of infection with six out of seven (85.7%) mice showing clearance of infection by day 19, while the control group continued to show infection till day 29.

The efficacy of an ethyl acetate fraction of *P*. *guajava* leaf extracted with water, on brown male chicks experimentally-infected with *E*. *coli* O78, was monitored by clinical signs (Geidam et al., 2015). The results showed that intestinal villi returned to normal after being treated with the fraction at a dose of 100 mg/kg for 7 days. Additionally, the bacterial shedding load of infected chicks treated with the fraction at 50 mg/kg and 100 mg/kg significantly reduced on day 8, while appetite improved, depression reduced, and an increase in body weight gain on day 6 was observed.

*P*. *guajava* ethanolic leaf extract (at 200 and 400 mg/kg) demonstrated a significant decrease in the total number of diarrheal stools, weight of stools and mean defecation rate of stools in enteropathogenic *E*. *coli* (EPEC)-infected Wistar rats after 6 and 24 h (Hirudkar et al., 2020b). Additionally, a significant reduction in the level of EPEC in stools was observed after 4 h of treatment. The leaf extract was as efficient as quercetin at 50 mg/kg and norfloxacine at 5.7 mg/kg. A model of *S. flexneri*-infected rat treated with *P*. *guajava* leaf extract at doses of 100, 200 and 400 mg/kg demonstrated a similar significant decline in total number of diarrheal stools, weight of stools and the stool water content on the first, third and fifth days after diarrhea induction and a significant reduction in the density of *S*. *flexneri* in stools of rats after the third day (Hirudkar et al., 2020a). Here again, the leaf extract was as efficient as quercetin at 50 mg/kg and norfloxacine at 5.7 mg/kg. The administration of an ethanolic *P*. *guajava* leaf extract at doses of 50, 100 and 200 mg/kg demonstrated a reduction in the incidence of diarrhea in weaned piglets infected with enterotoxigenic *E. coli* (ETEC) (Wang et al., 2021). The impact on intestinal structure and function was evaluated, as damage to these aspects can facilitate the invasion and colonization of potential pathogens, leading to diarrhea. The results revealed a significant increase in villus height (*p* < 0.001) and the villus height to crypt depth ratio (*p* < 0.001), as well as a decrease in crypt depth (*p* < 0.001) in the jejunum compared with negative group. In addition, the supplementation of 50, 100, and 200 mg/kg of *P*. *guajava* extract in diets also showed 14.3%, 8.9%, and 7.1% diarrhea incidence reduction, respectively. Based on these findings, it is confirmed that *P*. *guajava* leaf extract has a protective role in maintaining intestinal health and reducing the susceptibility to ETEC- and EPEC-induced diarrhea in mammals.

##### 3.3.2.3 Castor oil-induced diarrhea model

Castor oil is a vegetable oil obtained from the bean of *Ricinus communis* L., and is commonly used as a laxative drug in mice model of diarrhea (Chassagne, 2022).

The methanolic extract of *P*. *guajava* leaves, at a dose of 400 mg/kg, significantly reduced the weight of feces to 3.5–4 mg, compared to the average weight of feces (10.5 mg) in the control group, and decreased the frequency of droppings in a model of castor-oil induced diarrhea in rats (Lin et al., 2002). In another study, healthy mice were pre-administrated with 0.5 mL/20 g of castor oil, then the animals received a guava leaf ethanolic extract at a concentration of 750 mg/kg, and three out of five mice showed cessation of diarrhea after 120 min (Maïkere-Faniyo et al., 1989).

Treatment with an ethanolic extract of *P*. *guajava* leaves at doses of 250, 500 and 750 mg/kg induced a significant decline in the frequency of defecation (with 10%, 26% and 52% inhibition, respectively) and wetted feces (with 16%, 43% and 63% inhibition, respectively) compared to the control group (Mazumdar et al., 2015).

A methanolic extract of *P*. *guajava* leaf, administered orally at doses of 50, 100, and 200 mg/kg, produced a significant inhibitory effect on diarrhea induced by castor oil in mice (Olajide et al., 1999). After oral pretreatment with the extract, the mice exhibited a remarkable decrease in the number of wet feces, with 60, 71% and 83% of reduction compared to the control group.

##### 3.3.2.4 Drug induced diarrhea model

In a rat model of lactose-induced diarrhea treated with two oral doses (50 and 100 mg/kg) of *P*. *guajava* leaf extract, the kidney weight, serum electrolyte level, urinary volume, along with the elimination of electrolytes in urine, and nephrotic function were returned to near the level of the control animals (Koriem et al., 2019). The highest dose (100 mg/kg) of *P*. *guajava* leaf extract was as effective as standard drug desmopressin (0.2 mg/kg).

Prostaglandin E_2_ (PGE_2_) was used to induce a significant increase in the fluid volume of the rat intestine. Both aqueous and methanolic extracts of *P*. *guajava* leaf (400 mg/kg) showed the ability to inhibit PGE_2_-induced enteropooling in rats, with a reduction of the volume of intestinal fluid of 47% and 57%, respectively compared to the PGE_2_-induced group (Lin et al., 2002).

Ethyl acetate and n-butanol fractions from *P*. *guajava* leaves ethanolic extract and aqueous decoction were tested on a mice model of *Folium Sennae*-induced diarrhea (Lu et al., 2020). Butanol extract and aqueous decoction were the most effective to decrease loose stools rate, diarrhea rate, diarrhea index, and intestinal propulsion (charcoal method) of mice at a dose of 2.5 g/kg.

Overall, these studies demonstrate the role of *P. guajava* leaves in increasing water absorption in the colon, reducing gastrointestinal propulsion rates and motility, reducing the susceptibility to ETEC and EPEC infections in mammals, and reducing the number of feces in drug-induced diarrhea models.

#### 3.3.3 Antispasmodic activity

The contractions of the smooth muscles in the intestines lead to rapid transit of food residues through the digestive system, resulting in loose or watery bowel movements (Nigam et al., 2019). Therefore, to relieve diarrhea-related symptoms, it is necessary to reduce the intensity of contractions, and so to use antispasmodic drugs as they exhibit anticontractile effects. To perform antispasmodic activity test, isolated intestinal tissues obtained from living animals, including the guinea ileum and jejunum, are utilized as a model (Chassagne, 2022).

An ethanolic extract of guava leaves showed a concentration-dependent (from 200 μg/mL to 1.6 mg/mL) inhibition in the coaxially electrically-stimulated guinea-pig isolated ileum, with an initial increase in muscular tone, followed by a gradual decrease (Lutterodt, 1989). When the extract concentration reached 800 μg/mL, a sharp decrease in contractility after the initial period of complete blockade was observed. Acetylcholine (ACh) is released from postganglionic nerve endings by transmural shock, leading to spasmodic contractions in the longitudinal muscle (Lutterodt, 1989). Aqueous extracts of *P*. *guajava* leaf and stem bark at a concentration of 80 μg/mL in organ bath, were highly active with more than 80% inhibition of ACh or KCl solution-induced contractions in isolated guinea-pig ileum. (Tona et al., 1999). Another study showed that the peristaltic reflex of isolated guinea pig ileum was inhibited by a *P*. *guajava* leaf extract and one chromatographic fraction (no details of the dose was provided) (Lozoya X et al., 1994). An ethanolic extract of guava leaf reduced ACh-induced contractions of an isolated rabbit jejunum at a concentration of 333.3 μg/mL and inhibited them completely at a higher dose (1,333.3 μg/mL) (Ibeh et al., 2021).

Overall, *P. guajava* leaves showed antispasmodic activity in isolated guinea pig ileum and rabbit jejunum in a dose-dependent manner.

#### 3.3.4 Anti-inflammatory activity

Inflammation is an established etiopathology of diarrhea. For example, acute infectious diarrhea can be classified as noninflammatory and inflammatory diarrhea, the latter being caused by cytotoxin-producing non-invasive organisms (e.g., enteroaggregative or enterohemorrhagic *E*. *coli*) or invasive organisms (e.g., *Salmonella* spp., *Shigella* spp.) (Navaneethan and Giannella, 2008). Moreover, inflammation is also central to the diarrhea in patients with chronic inflammatory bowel diseases such as Crohn’s disease or ulcerative colitis (Binder, 2009). To test the anti-inflammatory activity of plant products, pro-inflammatory and anti-inflammatory cytokines can be evaluated. A reduction in pro-inflammatory cytokines (IL-1β, IL-6 and TNF-α) and an increase in anti-inflammatory cytokines (IL-10) indicate anti-inflammatory effects.

##### 3.3.4.1 Inflammatory cytokines

The anti-inflammatory property of a *P*. *guajava* leaf ethanolic extract was investigated in an EPEC-infected model on Wistar rats (Hirudkar et al., 2020b). EPEC mediated infectious diarrhea caused a significant increase in the expression of pro-inflammatory cytokines (IL-1β and TNF-α) and production of nitric oxide (NO), and the expression of these cytokines and level of NO significantly decline (*p* < 0.05) after a treatment with the extract at 200 mg/kg. It was also reported in another study that the *in vivo* anti-inflammatory effect with ethanolic guava leaf extract (200 mg/kg) produced a significant decrease in production of NO and the expression of pro-inflammatory cytokines IL-6 and TNF-α in colonic tissue in a *S*. *flexneri*-induced diarrhea rat model (Hirudkar et al., 2020a). Guava seed polysaccharide (GSPS) and its purified fractions GSF1, GSF2 and GSF3 were found to have anti-inflammatory potential. GSPS, GSF2 and GSF3 at different concentrations (8, 40 and 200 μg/mL) significantly (*p* < 0.05) increased the secretions of anti-inflammatory cytokine IL-10 in lipopolysaccharide (LPS)-stimulated peritoneal macrophages, while GSF1 slightly increased IL-10 cytokine secretions (Lin and Lin, 2020). GSPS, GSF1, GSF2 and GSF3 administrations also significantly (*p* < 0.05) reduced the production of IL-6 and the ratios of IL-6/IL-10. However, GSPS and GSF1 administrations significantly (*p* < 0.05) increased the production of IL-1β. The results showed that GSPS and its purified polysaccharide fractions, particularly GSF3, have strong anti-inflammatory potential. *P*. *guajava* ethanolic leaf extract (at doses of 50, 100 and 200 mg/kg) administered to weaned piglets infected with ETEC resulted in a significant reduction in serum levels of TNF-α (*p* < 0.001), IL-1β (*p* < 0.001), and IL-6 (*p* = 0.002) (Wang et al., 2021). Additionally, the mRNA expression of TNF-α (*p* < 0.001), IL-1β (*p* < 0.001), and IL-6 (*p* < 0.001) in the jejunum mucosa was also significantly decreased compared to the negative control group. Positive control was quinocetone (50 mg/kg).

##### 3.3.4.2 NF-κB pathway

Nuclear factor-κB (NF-κB), an inducible transcription factor, plays an important role in regulating the expression of pro-inflammatory genes (Liu et al., 2017). A clear inhibition of NF-κB reporter gene expression in murine fibrosarcoma L929sA cells was observed after pretreatment with 62.5 μg/mL dichloromethane-methanol *P*. *guajava* leaf extract (Kaileh et al., 2007). Another study demonstrated the anti-inflammatory activity of psiguadial B by suppressing the NF-κB pathway in LPS-induced immune cells (Man Kadayat et al., 2021). The results showed that psiguadial B at a dose of 10 µM significantly reduced the production of NO, and the expressions of both TNF-α and IL-6 in LPS-induced immune cells. Another study also reported the anti-inflammatory activity of the ethyl acetate fraction of guava leaf extract by suppressing the NF-κB pathway in LPS-induced *Labeo rohita* head-kidney macrophages (Sen et al., 2015). The ethyl acetate fraction inhibited the NO production (75% inhibition at the highest dose) along with the production of TNF-α and IL-1β in a concentration-dependent manner (50, 100 and 200 μg/mL).

##### 3.3.4.3 Others

Anti-arachidonate-5-lipoxygenase (A5-LOX), anti-hyaluronidase, xanthine oxidase enzyme inhibitory assays and NO production inhibitory assay in LPS activated RAW 264.7 macrophages were used to evaluate the anti-inflammatory activity (Perera et al., 2016). The ethanolic extract of *P*. *guajava* leaf exhibited anti-A5-LOX activity with half maximal inhibitory concentration (IC_50_) value of 46.5 ± 2.5 μg/mL, moderate inhibition against hyaluronidase activity (45.1% inhibition at 500 μg/mL), xanthine oxidase enzyme inhibitory activity (40.5% inhibition at 250 μg/mL), and NO production inhibitory activity at a dose of 500 μg/mL with 7.9% ± 0.3% inhibition. In another study, anti-inflammatory activity was assessed by examining the effect of guava extracts on leukocyte recruitment into the peritoneal cavity in carrageenan-induced peritonitis in mice (de Araújo et al., 2014). Female Swiss mice were administrated orally aqueous and 70% acetone extracts at doses of 50, 100 and 200 mg/kg, respectively. Results showed that both extracts at all of the concentrations tested significantly reduced the number of leukocyte from peritoneal exudates, demonstrating an inhibitory effect on cell recruitment into the peritoneal cavity. In addition, one study detected anti-inflammatory activity by injecting carrageenan in the right hind paw of male Swiss mice and Wistar rats, and by measuring the inhibition of oedema (Olajide et al., 1999). Rats were orally administered with the *P*. *guajava* leaf extract (at doses of 50, 100 and 200 mg/kg) 1 h before carrageenan injection. Results showed that *P*. *guajava* extract exhibited anti-inflammatory activity in a dose-dependent manner, with 46% inhibition (at 50 mg/kg), 56% inhibition (at 100 mg/kg) and 77% inhibition (at 200 mg/kg), respectively.

*P*. *guajava* leaf extract was administrated to *V. cholerae*-infected mice (Shittu et al., 2016). The infected mice showed enterocytes degeneration and necrosis of intestinal epithelial cells, along with infiltration of inflammatory cellular in the lamina propria. After treatment with guava leaf extract at a dose of 250 mg/kg, a reduction in the degeneration and necrosis of enterocytes, in the inflammatory exudates in the lamina propria, in the hyperplasia of goblet cells and stumpy and club-shaped villi were observed, while the regeneration of enterocytes were increased. These results demonstrate the anti-inflammatory effects and a positive effect on enterocytes regeneration of *P. guajava* in mice infected by *V. cholerae*.

Overall, *P*. *guajava* leaves (and seeds, to a lesser extent) showed an anti-inflammatory effect by acting on cytokines production, inhibiting the NF-κB pathway, and inhibiting the arachidonic acid cascade (*via* the lipoxygenase pathway), while also allowing a regeneration of enterocytes and reducing leukocytes recruitment.

#### 3.3.5 Anti-parasitic activity

*Entamoeba histolytica*, a protozoan parasite, is capable of causing intestinal amebiasis (Mortimer and Chadee, 2010). Intestinal amebiasis is a primary contributor to diarrheal illnesses worldwide (Shirley et al., 2018). Aqueous decoction of *P*. *guajava* stem bark exhibited a strong *in vitro* antiamoebic activity (against *E*. *histolytica*) with minimal amoebicidal concentration (MAC) less than 7.8 μg/mL, while the aqueous extract of leaf showed antiamoebic activity with MAC = 62.5 μg/mL (Tona et al., 1998; 1999). Tona et al. also reported the antiamoebic activity of n-butanol extract (polyphenol) and crude saponin extract prepared from aqueous decoction of *P*. *guajava* stem bark. The n-butanol fraction exhibited the strongest antiamoebic activity with a MAC of 2.6 μg/mL, and the crude saponin fraction inhibited *E*. *histolytica* growth with MAC value 8.3 μg/mL (Tona et al., 2000).

*Giardia lamblia* is a protozoan parasite responsible for giardiasis, a disease recognized as a leading global contributor to diarrhea (Gaona-López et al., 2023). Ethanolic maceration extract of *P*. *guajava* leaf had an inhibitory effect on the growth of *G*. *lamblia* with an IC_50_ value of 457.9 ± 25.1 μg/mL and IC_50_ value of 439.8 ± 24.1 μg/mL for percolated extract (Neiva et al., 2014). The aqueous extract of *P*. *guajava* bark showed an anti-giardial activity with a dose dependent effect with 82.2% of loss of trophozoite viability at 2.5 mg/mL. Metronidazole (50 µM) was used as positive control (Brandelli et al., 2009). One study also demonstrated that in *Giardia*-infected mice treated with 75 mg/kg/day of *P*. *guajava* hydro-ethanolic leaf extract, the number of *Giardia* trophozoites was reduced by 84.4% compared to negative control. Metronidazole at 15 mg/kg/day was used as positive control (Khedr et al., 2021).

Overall, *P. guajava* stem bark was shown to possess *in vitro* antiamoebic activity while *P. guajava* leaves was reported to have *in vitro* and *in vivo* anti-giardial activity.

#### 3.3.6 Antiviral activity

Rotaviruses are widely recognized as the major pathogens of diarrhea in infants, young children and animals (Alaoui Amine et al., 2020). The aqueous extract of *P*. *guajava* leaves at a concentration of 8 μg/mL inhibited the growth of two viruses: simian (SA-11) and human (HCR3) viruses with an inhibition of 47.5% and 93.8%, respectively (Gonçalves et al., 2005). Another study also reported the *in vitro* antiviral activity of the ethanolic extract of *P*. *guajava* leaves against simian virus (SA-11) (propagated in MA-104 cells monolayers) (Cecílio et al., 2012). However, after treatment with *P*. *guajava* extract at doses of 50 and 500 μg/mL, no inhibition on cytopathic effect of rotavirus on the treated MA-104 cells monolayers was evidenced.

In conclusion, *P. guajava* was reported to possess various biological activities related to diarrheal disorders including antibacterial activity (against diarrheal-causing pathogens such as ETEC, EPEC, *Salmonella* spp., *Shigella* spp., *V. cholerae*), antiparasitic activity (against *E. histolytica* and *G. lamblia*), antiviral activity (against rotaviruses), reduction in gastrointestinal propulsion rates and motility, reduction in the susceptibility to ETEC and EPEC infections in mammals, reduction in the number of feces in drug-induced diarrhea models, anti-inflammatory and antispasmodic properties. Most of these studies have been performed on *P. guajava* leaf extracts while a few studies focus on stem bark (antiparasitic activity) and seeds (anti-inflammatory effect), and some other studies have tested *P. guajava* fractions or compounds. While a large number of these studies are *in vitro* assays, a non-negligible number of studies have been performed *in vivo* (antibacterial, ant-inflammatory properties) and *ex vivo* (anti-spasmodic activity). All these studies confirm the role of *P. guajava* (especially leaves) as an antidiarrheal agent and demonstrate the multifactorial mechanisms of its activity.

### 3.4 Phytochemistry

**FIGURE 5:**
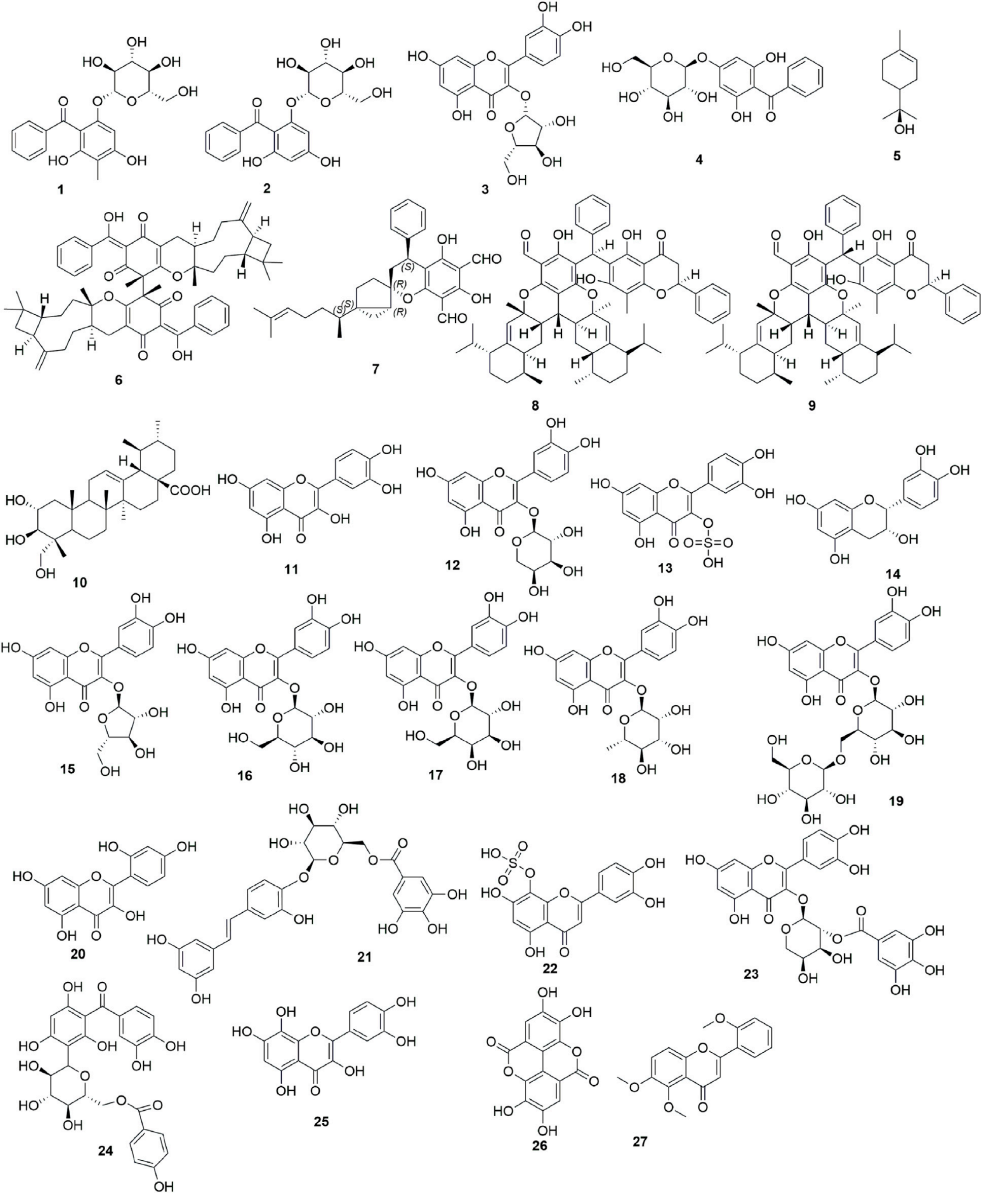
Compound structures that have been isolated from or are present in *P*. *guajava*. Guajaphenone A (**1**), garcimangosone D (**2**), guaijaverin (**3**), 2,6-dihydroxy-4-*O*-β-D-glucopyranosylbenzophenone (**4**), α-terpineol (**5**), psiguajdianone (**6**), psidial F (**7**), psidguajones A and B (**8–9**), asiatic acid (**10**), quercetin (**11**), quercetin-3*-O*-arabinoside (**12**), quercetin-3-*O*-sulfate (**13**), epicatechin (**14**), avicularin (**15**), quercetin 3-*O*-β-D-glucoside (isoquercetin) (**16**), quercetin 3-*O*-β-D-galactoside (hyperin) (**17**), quercetin 3-*O*-β-L-rhamnoside (quercitrin) (**18**), quercetin 3-*O*-gentiobioside (**19**), morin (3,5,7,2′,4′-pentahydroxyflavone) (**20**), piceatannol 4′-galloylglucoside (**21**), 8-hydroxyluteolin 8-sulfate (**22**), quercetin 3-(2″*O*-galloyl-α-L-arabinopyranoside) (**23**), maclurin 3-C-(6″-p-hydroxybenzoyl-glucoside) (**24**), gossypetin (**25**), ellagic acid (**26**), 5,6,2′-trimethoxyflavone (**27**).

Researchers have illustrated the complex composition of *P*. *guajava* by applying various chemical separation and identification technologies. To date, 27 chemical components have been described as responsible for the antidiarrheal activity and other related activities. These compounds can be classified into six categories: four benzophenone glycosides (**1**-**4**), six terpenes (**5**-**10**), polysaccharides (not shown), 10 flavonoids (**11**-**20**) and seven other active compounds (**21**-**27**). Their chemical structures are shown in Figure 5.

#### 3.4.1 Benzophenone glycosides

Bioactivity-guided fractionation of methanol extract from *P*. *guajava* leaves yielded a new benzophenone glycoside, guajaphenone A (**1**) together with two known compounds, garcimangosone D (**2**) and guaijaverin (**3**) (Ukwueze et al., 2015). These compounds were screened against bacteria using the broth microdilution method. They were found to have antibacterial activities against *E*. *coli* and *S*. *aureus* with MIC values ranging from 250 to 900 μg/mL. A benzophenone glycoside, 2,6-dihydroxy-4-*O*-β-D-glucopyranosylbenzophenone (**4**), was isolated from *P*. *guajava* leaves (Fu et al., 2011). Compound **4** exhibited significant activity in inhibiting the secretion of NO in mouse peritoneal macrophages, with an inhibition rate of 38.60% at a concentration of 10 μmol/L. However, it did not demonstrate significant growth inhibitory effect on the growth of mouse peritoneal macrophages.

#### 3.4.2 Terpenes

α-terpineol (**5**), a volatile monoterpene alcohol, is present in the essential oil of *P*. *guajava*. One study was performed to assess the antidiarrheal properties of compound **5** in the management of acute diarrhea and enteropooling induced by castor oil or PGE_2_ in Swiss mice (dos Santos Negreiros et al., 2019). The results showed that compound **5** (at concentrations of 6.25, 12.5, 25, and 50 mg/kg) was effective in reducing total stool mass (inhibitions of defecation were 54.9%, 48%, 44.4%, and 23.5%, respectively) and the total mass of diarrheal feces (inhibitions of diarrhea were 47.1%, 66%, 55.3%, and 10.3%, respectively) within 4 h after the administration of castor oil, as compared to the control (5 mg/kg loperamide). Compound **5** (12.5 mg/kg) also significantly reduced the volume of intestinal feces by 78% in mice with diarrhea induced by castor oil and decreased intestinal fluid by 38.8% in mice with PGE_2_-induced diarrhea.

Psiguajdianone (**6**) is a novel caryophyllene-derived meroterpenoid dimer, which was isolated from the leaves of *P*. *guajava* (Ning et al., 2019). Compound **6** exhibited inhibitory effects on the production of NO, TNF-α, and PGE_2_ induced by LPS in mouse RAW264.7 cells, with IC_50_ values of 9.1 ± 0.3, 12.6 ± 0.6, and 4.3 ± 0.6 μM, respectively. These findings suggest that compound **6** could serve as a promising lead molecule for the development of new anti-inflammatory agents.

One meroterpenoid named psidial F (**7**) was isolated from the leaves of *P*. *guajava* (Yu et al., 2022). Compound **7** was found to possess anti-inflammatory activity by inhibiting LPS-stimulated NO release in primary macrophages, with an inhibition rate of 62.6% at a dose of 10 µM. Psidguajones A and B (**8**-**9**), a pair of complex meroterpenoid epimers were isolated from the leaves of *P*. *guajava* (Liu et al., 2021). Compound **9** showed a moderate cytotoxic effect on HeLa cells at a concentration of 10 μM with about 76% cell viability. Asiatic acid (**10**) was also isolated from the leaves of *P*. *guajava*, and showed a dose dependent (10–500 mg/mL) spasmolytic activity in spontaneous movements of isolated rabbit jejunum preparations with EC_50_ value of 80 ± 7.8 μg/mL (Begum et al., 2002).

#### 3.4.3 Polysaccharides

Guava seed polysaccharide (GSPS) and guava seed polysaccharide fraction 1 (GSF1), GSF2 and GSF3 were found to have anti-inflammatory potential (see pharmacological section) (Lin and Lin, 2020). GSF3 is a major active proteopolysaccharide component in GSPS, composed of glucuronic acid, galacturonic acid, galactose, mannose, glucose, arabinose, ribose, xylose, fucose and rhamnose.

#### 3.4.4 Flavonoids

Quercetin (**11**) and quercetin-3-arabinoside (**12**) were isolated from *P*. *guajava* leaves. Different doses of compound **11** (0.8–12.8 μg/mL) and **12** (80 µg/mL-1.28 mg/mL) were administrated to coaxially stimulated guinea-pig isolated ileum followed by administration of naloxone (Handal et al., 1983). Naloxone, an opiate antagonist, is well-known for its remarkable capacity to counteract the effects of narcotic drug overdose. In a guinea pig ileum model, the inhibition of contractions produced by 12.8 ng/mL morphine was reversible within 3 min by 40 ng/mL naloxone, while the same effect produced by 12.8 μg/mL quercetin (**11**) could not be antagonized by similar doses of naloxone, demonstrating an effect on acetylcholine release not mediated by opiate receptors (Lutterodt, 1989). Another study also reported the effect of quercetin on growth inhibition and virulence features of common diarrheal pathogens viz. colonization of epithelial cells and production and action of enterotoxins (Birdi et al., 2010). Quercetin inhibited the invasion of both enteroinvasive *E*. *coli* and *S*. *flexneri* into HEp-2 cells. However, it had no antibacterial activity at the concentrations used (2, 20, 100, 200 μg/mL) and it did not affect adherence of enteropathogenic *E*. *coli*. Quercetin was confirmed as a major biomarker for the observed antidiarrheal potential of *P*. *guajava* against *S*. *flexneri*-induced infectious diarrhea in a model of diarrheal rat, as it significantly reduced the density of *S*. *flexneri* in stools, water content of stools and restored the alterations observed in blood parameters, and pro-inflammatory cytokines (IL-6 and TNF-α) expression at a dose of 50 mg/kg (Hirudkar et al., 2020a). In addition, quercetin also significantly reduced diarrheal stools, the expression of IL-1β and TNF-α, and increased the NO level in an EPEC-induced diarrhea rat model (Hirudkar et al., 2020b). A study reported a major phenolic compound in *P*. *guajava* which was determined as quercetin-3-*O*-sulfate (**13**). However, the authors did not perform any bioactive test (Nguyen et al., 2023).

Epicatechin (**14**), quercetin (**11**) and its glycosides (pentoside and hexoside) were detected in leaves, peel and flesh of white guava. The flavonoid extracts of white guava leaves had antibacterial effects on *E*. *coli* and *S*. *aureus* with MIC value of 2.5 and 0.313 mg/mL, respectively (Zhang et al., 2018). Quercetin glycosides were isolated from methanolic extract of *P*. *guajava* leaves and identified as quercetin 3-*O*-α-L-arabinoside (guajaverin) (**15**), quercetin 3-*O*-β-D-glucoside (isoquercetin) (**16**), quercetin 3-*O*-β-D-galactoside (hyperin) (**17**), quercetin 3-*O*-β-L-rhamnoside (quercitrin) (**18**) and quercetin3-*O*-gentiobioside (**19**). Compound **16** and **17** showed a slight spasmolytic effect on isolated guinea pig ileum (with 28.7% and 5.0% of inhibition, respectively) (Lozoya X et al., 1994).

Morin (3,5,7,2′,4′-pentahydroxyflavone) (**20**), a bio-available flavonoid, is a major component of traditional medicinal plants such as *P*. *guajava*. Morin showed anti-bacterial activities against *V*. *cholerae* with an IC_50_ value of 50 µM and an MIC of 150 µM (Nag et al., 2021). It also demonstrated that about 20% of human lung fibroblast (WI38) and human intestinal epithelial (HIEC-6) cells were killed in 24 h *in vitro* at a concentration of 2 mM, indicating low cytotoxicity.

#### 3.4.5 Dereplication strategies to highlight bioactive compounds

In Olatunde et al. (2021), the ethanolic guava leaf extract (with chlorophyll removal using the sedimentation process) contained piceatannol 4′-galloylglucoside (**21**), epicatechin (**14**), 8-hydroxyluteolin 8-sulfate (**22**), quercetin 3-(2″-galloyl-α-L-arabinopyranoside) (**23**), and maclurin 3-C-(6″-p-hydroxybenzoyl-glucoside) (**24**), detected by liquid chromatography–mass spectrometry (LC-MS). This extract showed promising antibacterial properties against *E*. *coli*, *P*. *aeruginosa*, *V*. *parahaemolyticus* with MIC values ranging from 32 to 128 μg/mL (Olatunde et al., 2021). The n-hexane, dichloromethane, ethyl acetate, and aqueous partitions of the ethanolic *P*. *guajava* crude extract were tested for their antibacterial effect (Hall et al., 2023). Results showed that the MIC values of ethyl acetate and aqueous partitions against *E*. *coli*, *S*. *aureus* and *S*. Typhimurium ranged from 1.25 to 7.5 mg/mL. Using LC-MS chemical fingerprinting, tandem mass spectrometry (MS/MS) experiments and Global Natural Product Social Molecular Networking (GNPS), 18 compounds of interest were detected from these two partitions, including 10 bioactive compounds and eight non-bioactive compounds. The known bioactive compounds were gossypetin (**25**), quercetin (**11**), avicularin (**15**), ellagic acid (**26**), 5,6,2′-trimethoxyflavone (**27**), and epicatechin (**14**).

#### 3.4.6 Others

Lectins purified from crude extract (macerated with phosphate buffer solution pH 7) of *P*. *guajava* fruits showed an inhibition on the hemagglutinating activity of enterohemorrhagic *E*. *coli* (ETEC).

### 3.5 Toxicology

#### 3.5.1 Cytotoxicity

In a study assessing the impact of *P*. *guajava* leaves infusion on chromosomes and the cell cycle, *Allium cepa* L. root-tip cells and Wistar rat bone marrow cells served as *in vivo* plant and animal test systems, respectively (Teixeira et al., 2003). Human peripheral blood lymphocytes were employed as an *in vitro* test system. The investigation revealed no statistically significant alterations in either the cell cycle or the number of chromosome alterations when compared to untreated controls, following treatments with the infusion (2.6 and 26.2 mg/mL), in rat cells or cultured human lymphocytes. Nevertheless, it is noteworthy that at higher concentrations, the infusion induced a statistically significant inhibition of cellular division in *A. cepa* L. root-tip cells. Another study reported the effect of *P*. *guajava* infusions on the chromosome breaking activity in human lymphocytes *in vitro* (Roncada et al., 2004). Mitomycin C (MMC) and cytosine-β-arabino-furanoside (Ara-C) were used as chromosome aberration inducers. The infusion at a dose of 0.3 mg/mL significantly decreased the number of cells with breaks when associated with Ara-C.

The cytotoxicity of *P*. *guajava* leaves and branch extracts against peritoneal macrophages was studied (Delgado-Altamirano et al., 2017). The median cytotoxic concentration (CC_50_) value of dichloromethane extract and dichloromethane/methanol (1:1) extract were more than 200 μg/mL, while the CC_50_ of aqueous extract was 96.2 ± 0.6 μg/mL. Cecílio et al. (2012) observed that the ethanolic extract of *P*. *guajava* leaves induced no cellular morphologic changes on rhesus monkey kidney cell line MA-104 at a dose of 500 μg/mL after 48 h of incubation (Cecílio et al., 2012). In the screening of the cytotoxicity of 33 ethnopharmacologically selected medicinal plants from the Democratic Republic of Congo, the CC_50_ value of a decoction of *P*. *guajava* leaves against MRC-5 cells (human lung fibroblasts) was 32.9 μg/mL which can be interpreted as a high cytotoxic effect (Musuyu Muganza et al., 2012).

#### 3.5.2 Toxicology

In a toxicological study using the brine shrimp (*Artemia* sp.) lethality assay (BSLA), the ethanolic extract of *P*. *guajava* bark exhibited a median lethal concentration (LC_50_) of 12.5 μg/mL, which would classify it as toxic compared to the reference compound potassium dichromate (LC_50_ = 12.2 μg/mL) (Clemen-Pascual et al., 2022). Another study reported the toxicity of organic and aqueous extracts of *P*. *guajava* leaves (at 2 mg/mL) in BSLA, in which extracts were considered toxic if the mortality rate was 50% and more (Ramulondi et al., 2019). The findings revealed that the organic and aqueous leaf extract induced mortality rates of 7% and 11% respectively after 48 h. Similarly, the aqueous root extract demonstrated lethality in 20% of brine shrimps after 48 h, while the organic root extract exhibited a more substantial impact, causing mortality in 59% of brine shrimps within the same duration.

The acute toxicity evaluation of *P*. *guajava* leaf extract was also conducted using the median lethal dose (LD_50_) determination method (Ibeh et al., 2021). Seven groups of five Wistar rats were used and treated orally with doses of 500, 1,000, 2000, 3,000, 4,000, 5,000, and 6,000 mg/kg for each group. The rats were monitored for toxicity symptoms and mortality over a period of 24 h and 7 days. Results indicated that there were no obvious toxicity symptoms and no death observed with the highest dose of 6,000 mg/kg. Rats given 4,000 mg/kg of the extract appeared calm immediately after administration, but they regained agility and physical activity within 2 h and all remained active at the end of the study period. Based on these finding, the LD_50_ was determined to be more than 6,000 mg/kg, indicating that *P*. *guajava* leaves may be safe for oral use.

While certain extracts (i.e., extracts made from bark or root) of *P*. *guajava* demonstrated toxicity in the brine shrimp model, the absence of acute toxicity in Wistar rats suggests that the safety profile of *P*. *guajava* extracts may vary depending on the model, and on the extraction method and plant part used. Further research is needed to elucidate the mechanisms underlying these differential toxicological responses and to establish safe and effective dosing regimens for human use. Nonetheless, the overall findings support the potential safety of *P*. *guajava* leaf extract for oral consumption, warranting continued investigation into its therapeutic applications.

### 3.6 Clinical trials

A 5-day, randomized, parallel-group, multi-arm interventional study with a patient allocation ratio of 1:1:1:1 trial was conducted to evaluate the clinical efficacy of guava leaves decoction in the treatment of acute diarrhea in adults (Birdi et al., 2020). A total of 137 diarrheal patients (18–60 years) were enrolled based on their clinical symptoms, and 109 (57% women, 43% men) were included according to the inclusion and exclusion criteria. Three doses of guava leaves decoction (6-leaves, 10-leaves, and 14-leaves) were compared with controls receiving oral rehydration solution. The results indicated that the 14-leaves decoction (7.4 g) was the most effective in reducing stool frequency, improving stool consistency, and reducing nausea, abdominal pain, and episodes of vomiting. Administration of the decoction, three times a day helped the patients return to normalcy in 72 h, while it took 120 h for the control group. The safety of guava decoction was reflected by normal levels of hemoglobin, liver and kidney parameters, which did not significantly change during the treatment for all groups.

QG-5^®^, is a phytodrug with a standardized concentration of flavonoids (estimated as quercetin-equivalent 1 mg/500 mg capsule), developed from *P*. *guajava* leaves (Lozoya et al., 2002). A randomized, double-blind trial examined the efficacy of QG-5^®^ in 100 patients (20–96 years) with acute diarrheic disease. Patients were randomly distributed into two groups, the experimental group (n = 50) received 1 capsule of QG-5^®^ orally every 8 h for 3 days, while the control group (n = 50) received placebo capsules. Results showed a significant difference in the experimental group with a reduction in the number of abdominal pain episodes starting from the second day of administration compared to the control group. However, no significant changes were detected in the consistency and frequency of liquid stools compared with the control group.

## 4 Conclusion and future perspectives

This review summarized the ethnobotanical uses, pharmacological properties, phytochemical constituents, toxicological profiles, and clinical trials associated with *P*. *guajava* for the treatment of diarrhea based on scientific publications up to the year 2022. *P*. *guajava* is widely used in various countries across the Americas, Asia, Africa and Oceania for treating diarrhea and dysentery. Decoction and oral intake were the most common methods of preparation and administration, respectively. Additionally, *P*. *guajava* exhibits good efficacy whether administered alone or within herbal combinations. In the pharmacological studies, different models (bacteria, cell, parasite, organ, animal) were used *in vivo* or *in vitro* to test the antidiarrheal activity of crude extracts and bioactive compounds. Their antibacterial, antidiarrheal, antispasmodic, anti-inflammatory, antiparasitic, and antiviral activities have been demonstrated. To date, around 27 compounds exhibiting antidiarrheal or antibacterial activities related to diarrhea have been identified in *P*. *guajava*. These compounds include benzophenone glycosides, terpenes, polysaccharides and flavonoids. Among them, quercetin and its glycosides emerge as the predominant bioactive compounds. In the toxicity assessment, it was observed that a toxic response occurred when the brine shrimp was exposed to an ethanolic extract of *P*. *guajava* bark and organic root extract. However, no obvious toxicity effects were found when the rats were administrated with a 6,000 mg/kg dose of *P. guajava* leaf extract. We can hypothesize that the plant parts and organic solvents used may contribute to the differences in toxicity. Also, no side-effects were reported in the two clinical trials analyzed in this review. While extensive scientific research has been conducted to investigate *P*. *guajava*, the issues related to its utilization in clinical settings and to its development as a standardized drug persist. First of all, pharmacological research on guava have primarily focused on the examination of its crude extract. However, a standardized *P*. *guajava* extract is essential, as phytochemical variation arise depending on time of collection, season, soil, or climate. The use of QG-5^®^ in Mexico resulted in a notable reduction in abdominal pain, but it did not alleviate diarrheal symptoms. Secondly, pharmacological studies mainly involve the use of animal and cellular models, leading to the use of high doses and a lack of practical guidance for clinical studies. Consequently, there is a critical need for more targeted investigations that bridge the gap between preclinical observations and potential applications in clinical settings. Thirdly, the existing findings provide a foundational understanding of the toxicity of *P*. *guajava*. However, determining the safe dose range is a crucial step in optimizing the utility of the extract while minimizing any potential harm to living organisms. To evaluate the safety and efficacy of *P*. *guajava*, more comprehensive and systematic clinical trials are needed. Finally, while current research has provided the potential antidiarrheal properties of *P*. *guajava*, the antidiarrheal mechanism of *P*. *guajava* has not been fully elucidated. Further investigations are needed to reveal this/these mechanism(s).

Previous studies have successfully isolated compounds from *P*. *guajava*, particularly highlighting the prevalence of quercetin and its glycosides as predominant compounds with demonstrated antidiarrheal activities. Quercetin exhibits a spasmolytic effect, but its sole involvement in the overall antidiarrheal activity is questioned. Further investigations are needed to determine whether a synergistic interplay among diverse compounds contributes to the antidiarrheal effect. Consequently, there is a pressing need to identify and isolate these compounds from *P*. *guajava*.

## Data Availability

The original contributions presented in the study are included in the article/Supplementary Material, further inquiries can be directed to the corresponding author.

## Abbreviations

A5-LOX Arachidonate-5-lipoxygenase; ACh Acetylcholine; Ara-C Cytosine-β-arabin-furanoside; BSLA Brine shrimp lethality assay; BSM BaSO_4_ milk; CC_50_ Median cytotoxic concentration; EC_50_ Half maximal effective concentration; EPEC Enteropathogenic *E.scherichia coli*; ETEC Enterotoxigenic *Escherichia coli*; FIC Fractional inhibitory concentration; ΣFIC Mean fractional inhibitory concentration; GNPS Global natural product social molecular networking; GSF Guava seed fraction; GSPS Guava seed polysaccharide; HA Hemagglutination; IC_50_ Half maximal inhibitory concentration; IL-1β Interleukin 1 β; IL-6 Interleukin 6; IL-10 Interleukin 10; LC50 median lethal concentration; LC-MS Liquid chromatography-mass; LD50 median lethal dose; LPS Lipopolysaccharide; MAC Minimal amoebicidal concentration; MIC Minimum inhibitory concentration; MMC Mitomycin C; MS/MS Tandem mass spectrometry; NF-κB Nuclear factor-κB; NO Nitric oxide; ORS Oral rehydration salts; PGE_2_ Prostaglandin E_2_; PRISMA Preferred Reporting Items for Systematic Reviews and Meta-Analyses; RDAP Radial diffusion in two layers of perforated agar; TNF-α Tumor necrosis factor-α; UV Ultraviolet; WHO World health organization.
