# Supplementary material for: Ethnobotany, phytochemistry, and biological activities of Psidium guajava in the treatment of diarrhea: a review
Source: Front Pharmacol. 2024 Aug 23;15:1459066. doi: 10.3389/fphar.2024.1459066 (PMC11377350; doi:10.3389/fphar.2024.1459066)
Supplement: Supplementary file 1 [file Table1.docx]

**Ethnobotany, phytochemistry, and biological activities of *Psidium guajava* in the treatment of diarrhea: a review**

Supplementary files

Chengmei Liu^1^, Valérie Jullian^1^, François Chassagne^1^*

*^1^UMR 152 PharmaDev, Université Paul Sabatier (UPS), Institut de Recherche pour le Développement (IRD), Toulouse, France*

**TABLE S1**

**Table S1:** Ethnobotanical uses of *P*. *guajava* in the treatment of diarrheal disorders

| **Part of plants used** | **Country** | **Method of preparation** | **Method of administration** | **In formulas with** | **Indication** | **Type of articles** | **Study period** | **Informants** | **Sample size** | **Name of the book** | **Status** | **References** |
| --- | --- | --- | --- | --- | --- | --- | --- | --- | --- | --- | --- | --- |
| Branch (apex) | Panama | Decoction | Oral | ND | Diarrhea | Field survey | ND | Traditional practitioners | 11 | ND | ND | Gupta et al., 2005 |
| Bark | Belize | Crushed and decoction | Oral | ND | Diarrhea | Field survey | 1976 (May)-1978 (May) | ND | ND | ND | ND | Arnason et al., 1980 |
|  | Bolivia | ND | ND | ND | Diarrhea | Field survey | 2004 (March)-2006 (February) | Traditional practitioners (7), and inhabitants (19) | 26 | ND | Cultivated | Thomas et al., 2011 |
|  | India | Bark pasted or raw | ND | ND | Diarrhea, dysentery | Field survey | 2017-2019 | Traditional practitioners, and inhabitants | 84 | ND | ND | Thangliankhup et al., 2022 |
|  | Mauritius | Infusion | Oral | ND | Dysentery | Field survey | 2014-2015 | Traditional practitioners (9), and inhabitants (113) | 122 | ND | Cultivated, wild | Samoisy and Mahomoodally, 2016 |
|  | Peru | Decoction | ND | *Anacardium occidentale* and *ciruelo* (bark) | Dysentery | Field survey | 2004 (October)-2005 (August) | Inhabitants (rural) | 140 | NA | Cultivated | Sanz-Biset et al., 2009 |
|  |  |  | ND | ND | Diarrhea | Field survey | 2004 (October)-2005 (August) | Inhabitants (rural) | 140 | NA | Cultivated | Sanz-Biset et al., 2009 |
|  |  |  | Oral | ND | Diarrhea | Field survey | 2007 (May)-2008 (May) | Inhabitants | 119 | ND | ND | Odonne et al., 2013 |
|  |  | Grinded and decoction | Oral | ND | Diarrhea, dysentery | Field survey | 2010 (May-November) | Traditional practitioners (21), and inhabitants (99) | 120 | ND | Cultivated | Monigatti et al., 2013 |
| Bark, fruit (ripe), leaf (young) | French Polynesia | Chewed or crushed or decoction or raw | Oral | ND | Diarrhea | Field survey | 2021 (February-April) | Traditional practitioners (34), and inhabitants (99) | 133 | ND | ND | Chassagne et al., 2022 |
| Bark, fruit, leaf | Ecuador | Decoction | ND | ND | Diarrhea | Field survey | 2011 (July-October) | Inhabitants | 82 | ND | ND | Giovannini, 2015 |
| Bark, leaf | Brazil | ND | ND | ND | Diarrhea | Historical report | 1864, 1874, 1888, 1892, 1897 and 1920 | NA | NA | Formulary and Medical Guide (Formulário e GuiaMédico) | ND | Ricardo et al., 2017 |
|  | India | Decoction | Oral | ND | Diarrhea, (chronic), dysentery (chronic) | Field survey | 2014-2018 | Traditional practitioners (60), and inhabitants | 240 | ND | ND | Jamir et al., 2022 |
|  |  | Juice | Oral | ND | Diarrhea, dysentery | Field survey | 2015 (November)-2017 (October) | Traditional practitioners | 27 | ND | ND | Panmei et al., 2019 |
|  | Mexico | Infusion | ND | ND | Diarrhea, mucoid diarrhea | Field survey | 2005 (April)-2006 (August) | Inhabitants | 33 | ND | ND | Giovannini and Heinrich, 2009 |
|  | Nepal | Decoction | ND | *Bombax ceiba* (bark) and *Mangifera indica* (bark) | Diarrhea, dysentery | Field survey | 1999-2007 | Traditional practitioners (20), and inhabitants (25) | 45 | ND | ND | Ghimire and Bastakoti, 2009 |
|  | Peru | Decoction | Oral | ND | Diarrhea | Field survey | 2007 (May)-2008 (May) | Inhabitants | 119 | ND | ND | Odonne et al., 2013 |
|  | Uganda | Decoction | Oral | ND | Diarrhea | Field survey | 2004 (March-August) | Inhabitants | 205 | ND | ND | Ssegawa and Kasenene, 2007 |
| Bark, leaf, sprout | Brazil | Decoction | ND | ND | Diarrhea | Field survey | 2012 (March)-2013 (July) | Inhabitants | 22 | ND | Exotic | Tribess et al., 2015 |
| Bark, wood | Cambodia | Grilled on fire and decoction | Oral | ND | Diarrhea | Field survey | 2013 (April)-2014 (January) | Inhabitants | 202 | ND | ND | Chassagne et al., 2016 |
| Bud | Brazil | ND | ND | ND | Diarrhea | Field survey | ND | Inhabitants | 200 | ND | ND | Di Stasi et al., 2002 |
|  | Mauritius | Infusion | Oral | ND | Diarrhea | Field survey | 2014-2015 | Traditional practitioners (9), and inhabitants (113) | 122 | ND | Cultivated, wild | Samoisy and Mahomoodally, 2016 |
| Bud, leaf | Martinique | Decoction | Oral | ND | Diarrhea | Field survey | 1990 (September)-1994 (December) | Inhabitants | 100 | ND | ND | Longuefosse and Nossin, 1996 |
| Flower, fruit, leaf, root | India | Decoction or juice | Oral | ND | Dysentery | Field survey | 2012 (April)-2013 (May) | Inhabitants | 48 | NA | ND | Sivasankari et al., 2014 |
| Fruit | Brazil | Maceration | ND | ND | Diarrhea, dysentery | Field survey | 1996 (March)-1998 (October) | Traditional practitioners (6), and inhabitants (37) | 43 | ND | Cultivated | Coelho-Ferreira, 2009 |
|  | Ecuador | Infusion | ND | ND | Diarrhea | Field survey | 2014 (September)-2015 (June) | Inhabitants | 138 | ND | Cultivated | Caballero-Serrano et al., 2019 |
|  | India | Cooked | ND | ND | Diarrhea, dysentery | Field survey | 2017-2019 | Traditional practitioners, and inhabitants | 84 | ND | ND | Thangliankhup et al., 2022 |
|  | Mexico | Fresh | Oral | ND | Diarrhea, dysentery | Field survey | 1992 (January)-1994 (November) | Traditional practitioners (herbalists and midwives) | 13 | ND | ND | Frei et al., 1998 |
|  | United States | Raw | Oral | ND | Diarrhea | Historical report | 2014 | NA | NA | ND | ND | Soelberg et al., 2016 |
| Fruit (fresh, dried) | United Kingdom | Juice | Oral | ND | Diarrhea | Field survey | 2005-2007 (20 months) | Inhabitants | 23 | ND | ND | Ceuterick et al., 2008 |
| Fruit, leaf (young), stem bark | India | Decoction | Oral | ND | Diarrhea | Field survey | 2004 (September)-2005 (June) (5 months) | Inhabitants | 16 | ND | ND | Hajdu and Hohmann, 2012 |
| Leaf | Argentina | Decoction or infusion or soaked in water | ND | ND | Diarrhea | Field survey | 2007-2011 (8 months) | Inhabitants | 94 | ND | Cultivated | Kujawska and Hilgert, 2014 |
|  |  | ND | ND | ND | Bloody diarrhea | Field survey | 2014-2015, and 2019 (8 months) | Inhabitants | 85 | ND | ND | Kujawska and Schmeda-Hirschmann, 2022 |
|  | Benin | Decoction | ND | *Newbouldia laevis* (leaf), mixed with kaolin | Diarrhea, dysentery | Field survey | ND | Traditional practitioners, plant sellers, and patients | 785 | ND | ND | Dassekpo et al., 2020 |
|  |  | Powder | Oral | ND | Diarrhea | Field survey | 2018 (September) - 2019 (February) | Inhabitants | 506 | ND | Wild | Tchetan et al., 2021 |
|  |  | Raw | Oral | ND | Diarrhea | Field survey | ND | Inhabitants (breeders and farmers) | 690 | ND | ND | Ouachinou et al., 2019 |
|  | Brazil | Cataplasm | ND | *Campomanesia reitziana, Plinia peruviana* | Diarrhea | Field survey | 2012 (March)-2013 (July) | Inhabitants | 22 | ND | Exotic | Tribess et al., 2015 |
|  |  | Decoction | ND | *Lippia alba* (leaf) and *Spondias purpurea* (leaf) | Diarrhea | Field survey | 2017-2019 | Traditional practitioners | 27 | ND | ND | Almeida et al., 2022 |
|  |  | Decoction | ND | *Spondias purpurea* (leaf) | Diarrhea | Field survey | 2017-2019 | Traditional practitioners | 27 | ND | ND | Almeida et al., 2022 |
|  |  | Decoction | Oral | ND | Diarrhea | Field survey | 2016 (May)-2018 (March) (over 145 days) | Inhabitants | 8 | ND | ND | Yazbek et al., 2019 |
|  |  | Decoction or infusion | ND | ND | Diarrhea | Field survey | 2013 (July-November) | Inhabitants (elders) | 60 | ND | ND | Ribeiro et al., 2017 |
|  | Cambodia | Crushed with sugar and water | Oral | ND | Diarrhea | Field survey | 2013 (April)-2014 (January) | Inhabitants | 202 | ND | ND | Chassagne et al., 2016 |
|  |  | Decoction | Oral | *Aporosa villosa* (leaf) | Diarrhea prevention (post-partum) | Field survey | 2013 (April)-2014 (January) | Inhabitants | 202 | ND | ND | Chassagne et al., 2016 |
|  | Cameroon | Decoction | Oral | ND | Diarrhea, dysentery | Field survey | 1995, 1996 and 1998 | Traditional practitioners (local herbalists), inhabitants (elders) | ND | ND | ND | Noumi and Yomi, 2001 |
|  | China | Decoction | ND | ND | Diarrhea | Field survey | 2015 (August)-2016 (February) | Traditional practitioners, and inhabitants (farmers) | 27 | ND | Wild | Li and Xing, 2016 |
|  |  |  | ND | *Rhodomyrtus tomentosa* (leaf) | Diarrhea | Field survey | 2015 (August)-2016 (February) | Traditional practitioners, and inhabitants (farmers) | 27 | ND | ND | Li and Xing, 2016 |
|  |  | Infusion | Oral | ND | Diarrhea | Field survey | 2005-2006 | Traditional practitioners (indigenous), herbal medicinal hawkers/retailers | 52 | ND | ND | Au et al., 2008 |
|  |  | ND | Oral | ND | Diarrhea | Field survey | 2009 (March)-2012 (November) | Inhabitants | 45 | ND | ND | Zheng et al., 2013 |
|  | Cuba | Decoction | Oral | *Annona squamosa* (aerial part), *Ceiba pentandra* (fruit epicarp) | Diarrhea | Field survey | since 1983 | Traditional practitioners, and inhabitants (knowledgeable people) | 130 | ND | ND | Cano and Volpato, 2004 |
|  |  |  | Oral | *Punica granatum* (fruit epicarp) | Diarrhea | Field survey | since 1983 | Traditional practitioners, and inhabitants (knowledgeable people) | 130 | ND | ND | Cano and Volpato, 2004 |
|  | Ecuador | Infusion | Oral | ND | Diarrhea | Field survey | 2002 (September)-2003 (December) | Inhabitants | 1593 | ND | ND | Tene et al., 2007 |
|  | Eswatini | Decoction | Oral | *Albizia adianthifolia* (root) | Diarrhea | Field survey | 1998 (June)-2000 (March) | Traditional practitioners | ND | ND | ND | Amusan et al., 2002 |
|  | Fiji | ND | ND | ND | Diarrhea, dysentery | Field survey | 1991 (January-May) | Traditional practitioners | ND | ND | ND | McClatchey, 1996 |
|  | French Guiana | Decoction or juice | ND | ND | Diarrhea | Field survey | 2015 (February-May) | Inhabitants | 83 | ND | Cultivated | Tareau et al., 2017 |
|  | Guatemala | Decoction | ND | ND | Diarrhea, dysentery | Field survey | ND | ND | ND | ND | ND | Cáceres et al., 1993 |
|  | Guinea | Decoction | ND | ND | Diarrhea | Field survey | 2014 (October)- 2015 (June) | Traditional practitioners | 64 | ND | ND | Baldé et al., 2016 |
|  | India | Decoction | ND | ND | Bloody dysentery | Field survey | 2006 (April)-2008 (October) | Traditional practitioners (153), and inhabitants (84) | 237 | ND | Cultivated | Tangjang et al., 2011 |
|  |  |  | Oral | ND | Diarrhea | Field survey | 2008-2011 | Inhabitants | ND | ND | ND | Mallik et al., 2012 |
|  |  |  | Oral | ND | Dysentery | Field survey | 2017 (December)-2018 (December) | Inhabitants | 57 | ND | ND | Radha et al., 2022 |
|  |  | Juice | ND | ND | Dysentery | Field survey | 2008 (March)-2009 (February) | Inhabitants | 844 | ND | Cultivated | Upadhyay et al., 2010 |
|  |  | ND | ND | ND | Dysentery | Field survey | ND | Inhabitants | 237 | ND | ND | Beverly and Sudarsanam, 2011 |
|  |  | ND | Oral | *Mangifera indica* (bark), *Ananas comosus* (leaf) | Amebic dysentery, diarrhea | Field survey | 2009 (January)-2011 (June) | Inhabitants | 84 | ND | ND | Sharma et al., 2012 |
|  |  | Raw | ND | ND | Dysentery | Field survey | 2011 (September)-2013 (August) | Traditional practitioners | 66 | ND | ND | Vijayakumar et al., 2015 |
|  | Indonesia | Crushed, squeezed (not heated, with or without water), heated (not in water) | Local application or oral | ND | Diarrhea | Field survey | 2004 (2 months) | Traditional practitioners | 19 | ND | ND | Roosita et al., 2008 |
|  | Kenya | Decoction | ND | ND | Diarrhea | Field survey | ND | Inhabitants | 119 | ND | ND | (Njoroge and Bussmann, 2006) |
|  | Madagascar | Decoction | Oral | ND | Diarrhea | Field survey | 2016 (November-December) | Inhabitants | 103 | ND | ND | (Riondato et al., 2019) |
|  | Malaysia | Decoction | Oral | ND | Diarrhea | Field survey | 1996-1997 | Traditional practitioners (herbalists, shamans, midwives, collectors, traders), and inhabitants | 24 | ND | ND | (Ong and Nordiana, 1999) |
|  |  |  |  | ND | Diarrhea | Field survey | ND | Inhabitants | 24 | ND | Cultivated, exotic | (Rajoo et al., 2022) |
|  | Mauritius | Decoction | Oral | ND | Diarrhea | Field survey | 2011-2012 | Inhabitants (women) | 332 | ND | ND | (Suroowan and Mahomoodally, 2013) |
|  |  |  | Oral | ND | Diarrhea | Field survey | 2011–2012 | Inhabitants | 307 | ND | Cultivated, wild | (Nunkoo and Mahomoodally, 2012) |
|  | Mexico | Infusion | Oral | ND | Diarrhea | Field survey | 2000-2004 | Inhabitants | 69 | ND | ND | (Andrade-Cetto, 2009) |
|  |  |  |  | ND | Diarrhea | Field survey | 2011 (December)-2012 (May) | Inhabitants | 118 | ND | ND | (Juárez-Vázquez et al., 2013) |
|  |  |  |  | ND | Diarrhea | Field survey | 2015 (January)-2016 (July) | Traditional practitioners (nurses, physicians, pharmacists, and dentists) | 1614 | ND | ND | (Alonso-Castro et al., 2017) |
|  |  |  |  | ND | Diarrhea, dysentery | Field survey | 2001 (May)-2002 (April) | Inhabitants | 298 | ND | ND | (Canales et al., 2005) |
|  |  |  |  | ND | Diarrhea, dysentery | Field survey | 2011 (July-November) | Inhabitants | 162 | ND | ND | (Alonso-Castro et al., 2012) |
|  |  | ND | ND | *Cyrtocarpa edulis* (fruit) | Dysentery | Field survey | 2011 (July-November) | Inhabitants | 162 | ND | ND | (Alonso-Castro et al., 2012) |
|  |  | ND | ND | ND | Diarrhea | Field survey | 1985 (November)-1988 (March) | Traditional practitioners (15), and inhabitants (250) | 265 | ND | ND | (Heinrich et al., 1992) |
|  |  | ND | ND | ND | Diarrhea | Field survey | ND | Inhabitants | ND | ND | Cultivated | (Zamora-Martinez and de Pascual Pola, 1992) |
|  |  | ND | ND | *Ruta graveolens* (leaf), *Myrtus communis* (leaf) | Diarrhea | Field survey | 2011 (July-November) | Inhabitants | 162 | ND | ND | (Alonso-Castro et al., 2012) |
|  | Nigeria | ND | ND | ND | Dysentery | Field survey | ND | ND | ND | NA | ND | (Adamu et al., 2005) |
|  | Palestine | Decoction | Oral | ND | Diarrhea | Field survey | 2015 (February-June) | Traditional practitioners (herbalists) | 100 | ND | ND | (Jaradat et al., 2016) |
|  | Papua New Guinea | ND | Oral | ND | Diarrhea | Field survey | ND | ND | ND | ND | ND | (Prescott et al., 2012) |
|  | Peru | Decoction | Oral | *Citrus aurantifolia* (fruit juice) and *Anacardium occidentale* (leaf) | Diarrhea | Field survey | 2007 (May)-2008 (May) | Inhabitants | 119 | ND | ND | (Odonne et al., 2013) |
|  |  |  |  | *Citrus* spp. and *Anacardium occidentale* (leaf) and sugarcane alcohol | Diarrhea | Field survey | 2007 (May)-2008 (May) | Inhabitants | 119 | ND | ND | (Odonne et al., 2013) |
|  |  | Infusion | Oral | ND | Diarrhea | Field survey | 2006-2008 | Inhabitants | 30 | ND | ND | (Valadeau et al., 2010) |
|  | Philippines | Decoction | Oral | ND | Diarrhea | Field survey | 2008-2009 (6 weeks) | Inhabitants | 116 | NA | ND | (Abe and Ohtani, 2013) |
|  | Samoa | ND | ND | ND | Diarrhea | Field survey | 1984-1992 | Inhabitants | 7 | ND | Cultivated | (Cox, 1993) |
|  | South Africa | Crushed and decoction | Oral | ND | Diarrhea | Field survey | ND | Traditional practitioners | 100 | ND | ND | (Madikizela et al., 2012) |
|  |  | Crushed and mixed with cold, warm or hot water | Oral | ND | Diarrhea | Field survey | 2008 (February–March) | Households | 80 | ND | Cultivated | (de Wet et al., 2010) |
|  |  | Infusion | ND | ND | Diarrhea | Field survey | 2015 (January) | Inhabitants | 16 | ND | ND | (Hulley and Van Wyk, 2019) |
|  |  | ND | ND | ND | Diarrhea | Field survey | ND | ND | ND | ND | ND | (van Vuuren et al., 2015b) |
|  | Thailand | Decoction or infusion | Oral | ND | Diarrhea | Field survey | 2015–2017 | Inhabitants | ND | ND | Cultivated | (Panyadee et al., 2019) |
|  |  | Decoction or raw | Oral | ND | Diarrhea | Field survey | 2007 (March)-2008 (March) | Traditional practitioners (4), and inhabitants (34) | 38 | ND | Cultivated | (Srithi et al., 2009) |
|  |  |  |  | ND | Diarrhea | Field survey | 2015–2017 | Inhabitants | ND | ND | Cultivated | (Panyadee et al., 2019) |
|  |  | Raw | Oral | ND | Diarrhea | Field survey | 2004-2006 | Inhabitants | 30 | NA | ND | (Inta et al., 2008) |
|  |  |  |  | ND | Diarrhea | Field survey | 2013 (July)-2014 (January) | Traditional practitioners | 10 | ND | Cultivated | (Neamsuvan et al., 2016) |
|  | Uganda | Crushed in water, decoction | Oral | ND | Diarrhea | Field survey | 2017 (December)-2018 (June) | Traditional practitioners, herbal medicine gatherers, users of medicinal plants | 202 | ND | Cultivated | (Gumisiriza et al., 2019) |
|  |  | Decoction | Oral | ND | Diarrhea | Field survey | 2000 (June)-2001 (June) | Traditional practitioners (47), and households (126) | 173 | ND | Semi-wild | (Tabuti et al., 2003) |
|  | United States | Infusion | ND | ND | Diarrhea | Historical report | 2014 | NA | NA | ND | ND | (Soelberg et al., 2016) |
|  |  |  | ND | ND | Diarrhea | Historical report | Pre-1900 | NA | NA | Eggers, 1876 | ND | (Soelberg et al., 2016) |
|  | Vanuatu | Chewed | ND | ND | Diarrhea | Field survey | 2006 (May- November) | Inhabitants | 11 | ND | Cultivated, wild | (Bradacs et al., 2011) |
|  | Vietnam | Decoction | Oral | ND | Abdominal pain | Field survey | 2016 (April)-2017 (March) | Inhabitants | 93 | ND | ND | (Lee et al., 2019) |
| Leaf (dry) | Benin | ND | ND | ND | Dysentery | Field survey | ND | Inhabitants (mothers) | 1000 | ND | ND | (Allabi et al., 2011) |
| Leaf (fresh) | India | Chewed | Oral | ND | Diarrhea, dysentery | Field survey | 2007–2009 (2 years) | Traditional practitioners, and inhabitants (elders) | 10 | NA | Cultivated | (Kichu et al., 2015) |
| Leaf (mature) | India | Chewed or crushed in water | Oral | ND | Diarrhea | Field survey | 2002 (October)-2006 (June) | Traditional practitioners (27), and inhabitants (with knowledge in medicinal plants) (88) | 115 | ND | Cultivated | (Tetali et al., 2009) |
| Leaf (tender) | India | ND | ND | ND | Diarrhea | Field survey | 2017 (November)-2019 (May) | Homegardens owners | 100 | ND | Cultivated | (Roy et al., 2022) |
|  |  | Raw | Oral | ND | Diarrhea | Field survey | 2017 (November)-2019 (May) | Homegardens owners | 100 | ND | Cultivated | (Roy et al., 2022) |
| Leaf (young) | Bangladesh | Infusion | Oral | *Punica granatum* (bud) and babla (leaf) | Diarrhea | Field survey | 2010 (January)-2012 (June) | Inhabitants | 1280 | ND | ND | (Kadir et al., 2013) |
|  | Guadeloupe | Decoction | ND | ND | Diarrhea | Field survey | 2008 (August)-2009 (February) | Households from Terre-de-Bas (100), households from Terre-de-Haut (116) | 216 | ND | ND | (Boulogne et al., 2011) |
|  | Guinea | ND | ND | ND | Diarrhea | Field survey | 2015 (January)-2015 (May) | Traditional practitioners | 10 | ND | ND | (Baldé et al., 2016) |
|  | India | Raw | ND | ND | Bloody dysentery | Field survey | 2006 (April)-2008 (October) | Traditional practitioners (153), and inhabitants (84) | 237 | ND | Cultivated | (Tangjang et al., 2011) |
|  |  |  | ND | ND | Diarrhea | Field survey | ND | ND | ND | ND | ND | (Nautiyal and Goswami, 2022) |
|  | Indonesia | Infusion | Oral | ND | Diarrhea | Field survey | ND | Traditional practitioners, and inhabitants (elders) | ND | ND | ND | (Grosvenor et al., 1995) |
|  | Laos | Chewed | Oral |  | Diarrhea, infant diarrhea | Field survey | 2005 (December)-2010 (August) | Inhabitants | ND | ND | ND | (de Boer et al., 2012) |
|  | Malaysia | Chewed | Oral | ND | Diarrhea | Field survey | 1997 (June-October) | Traditional practitioners, and inhabitants | ND | ND | ND | (Ong and Nordiana, 1999) |
|  | Thailand | Raw | ND | ND | Diarrhea | Field survey | 2011 (June)-2012 (January) | Traditional practitioners | 9 | NA | Cultivated | (Neamsuvan et al., 2012) |
| Leaf, bud | Trinidad and Tobago | Decoction | Oral | ND | Diarrhea | Field survey | 1995 | Inhabitants | 130 | ND | ND | (Lans and Brown, 1998) |
|  |  |  |  | ND | Diarrhea | Field survey | 1995 (April- October) | Inhabitants | 111 | ND | ND | (Lans et al., 2000) |
| Leaf, fruit | Bolivia | Decoction | Oral | ND | Diarrhea | Field survey | 1995-1997 | Inhabitants | 21 | ND | ND | (Bourdy et al., 2000) |
|  | Mexico | Decoction | Oral | ND | Diarrhea | Field survey | 2000 (August)-2001 (April) | Traditional practitioners, and inhabitants (housewives, farmers) | 119 | ND | ND | (Hernández et al., 2003) |
|  | Philippines | Chewed | Oral | ND | Diarrhea | Field survey | 2008-2009 (6 weeks) | Inhabitants | 116 | NA | ND | (Abe and Ohtani, 2013) |
| Leaf, fruit bud | Brazil | ND | ND | ND | Diarrhea, dysentery | Field survey | 2011 (January-March) | Caregivers, patients (aged over 18 years) | 398 | ND | ND | (Neiva et al., 2014) |
| Leaf, leaf bud | Brazil | Decoction or infusion | Oral | ND | Diarrhea, dysentery | Field survey | 2008 (May-July) | Inhabitants | 91 | ND | Cultivated | (Cartaxo et al., 2010) |
| Leaf, root | Mexico | Infusion | Oral | ND | Diarrhea, dysentery | Field survey | 1992 (January)-1994 (November) | Traditional practitioners (herbalists and midwives) | 13 | ND | ND | (Frei et al., 1998) |
|  | Peru | Decoction | Oral | ND | Diarrhea | Field survey | 1994 (January) | Inhabitants | ND | ND | ND | (Desmarchelier et al., 1996) |
| Leaf, seed | Cameroon | Infusion | Oral | ND | Diarrhea | Field survey | 2019 (January-March) | Inhabitants | 47 | ND | ND | (Chouegouong et al., 2023) |
| Leaf, stem bark | Congo | Decoction | ND | ND | Diarrhea | Historical report | 1990 | ND | ND | Kambu, 1990 | ND | (Tona et al., 1998) |
|  |  |  | ND | ND | Diarrhea | Historical report | 1990 | ND | ND | Kambu, 1991 | ND | (Tona et al., 1999) |
| Leafbud | French Polynesia | ND | ND | *Cordyline fruticosa* (leafbud)*,* lime juice and coconut water | Diarrhea | Field survey | 2021 (February-April) | Traditional practitioners (34), and inhabitants (99) | 133 | ND | ND | (Chassagne et al., 2022) |
|  | Peru | Raw | Oral | ND | Diarrhea | Field survey | 2006-2008 | Inhabitants | 30 | ND | ND | (Valadeau et al., 2010) |
| ND | Brazil | ND | ND | ND | Diarrhea | Field survey | 2009 (June)-2011 (December) | Inhabitants (women) | 34 | ND | Cultivated | (Tuler and da Silva, 2014) |
| ND |  | ND | ND | ND | Diarrhea | Historical report | 1980-1990 | NA | NA | ND | ND | (do Nascimento Magalhães et al., 2019) |
| ND |  | ND | ND | ND | Diarrhea, dysentery | Field survey | 2010-2013 | Inhabitants | 393 | ND | ND | (Bieski et al., 2015) |
| ND |  | ND | ND | ND | Dysentery | Field survey | 2014 (May-October) | Inhabitants | 74 | ND | Cultivated | (de Santana et al., 2016) |
| ND | China | Decoction or raw | Oral | ND | Diarrhea | Field survey | 2008 (12 months) | Inhabitants (key informants include traditional healers, herbalists and plant collectors) | 128 | ND | ND | (Ghorbani et al., 2011) |
| ND | Congo | ND | ND | ND | Diarrhea | Field survey | 2019 (February-June) | Households | 300 | ND | Cultivated | (Mondo et al., 2021) |
| ND | India | ND | ND | ND | Diarrhea | Field survey | ND | Traditional practitioners | 59 | ND | ND | (Deb et al., 2015) |
| ND | Indonesia | ND | ND | ND | Diarrhea | Field survey | ND | Inhabitants (farmers) | 23 | ND | ND | (Mulyoutami et al., 2009) |
| ND | Kenya | Decoction | Oral | ND | Diarrhea | Field survey | 2009 (March-April) | Traditional practitioners(5), and inhabitants (5) | 10 | ND | ND | (Nagata et al., 2011) |
| ND | Mexico | ND | ND | ND | Diarrhea | Field survey | 1999 (March)-2000 (July) | Inhabitants | 8 | ND | ND | (Leonti et al., 2002) |
| ND |  | ND | ND | ND | Diarrhea, dysentery | Field survey | 1994 (February)-1996 (October) | Traditional practitioners (healers, midwives, hierbateros) | ND | ND | ND | (Heinrich et al., 1998) |
| ND | Pakistan | ND | ND | ND | Diarrhea | Field survey | 2012 (June)-2013 (May) | Traditional practitioners | 224 | ND | Cultivated | (Ahmed et al., 2014) |
| ND | South Africa | ND | ND | ND | Diarrhea | Field survey | 2015-2018 | Inhabitants | 37 | ND | ND | (Mhlongo and Van Wyk, 2019) |
| ND |  | ND | ND | ND | Diarrhea | Field survey | ND | ND | ND | ND | ND | (Lin et al., 2002) |
| ND | Thailand | ND | ND | ND | Diarrhea | Field survey | ND | Cancer patients | 120 | ND | ND | Poonthananiwatkul et al., 2015 |
| Root | Indonesia | Decoction | ND | *Glochidion* sp. (leaf), *Nypa fruticans* (leaf), *Justicia gendarussa* (leaf) | Bloody diarrhea | Field survey | ND | Traditional practitioners (dukuns) | 21 | ND | ND | Elliott and Brimacombe, 1987 |
|  | Kenya | Infusion | Oral | ND | Diarrhea | Field survey | 1997 | Inhabitants (mothers) | 7 | ND | ND | Geissler et al., 2002 |
|  | Martinique | Decoction | Oral | ND | Diarrhea | Field survey | 1991 (September)-1994 (December) | Inhabitants | 100 | ND | ND | Longuefosse and Nossin, 1996 |
|  | South Africa | Decoction | Oral | *Punica granatum* (root) | Diarrhea | Field survey | 2011 (January-July) | Traditional practitioners | 52 | ND | ND | Semenya et al., 2012 |
|  |  |  |  |  | Diarrhea | Field survey | 2011 (January-July) | Traditional practitioners | 52 | ND | ND | Semenya et al., 2012 |
|  |  | Infusion | Oral | *Punica granatum* (root or pericarp) | Diarrhea | Field survey | 2011 (January-July) | Traditional practitioners | 51 | ND | ND | Semenya and Maroyi, 2012 |
| Shoot | Brazil | Infusion | Bath, oral | ND | Diarrhea | Field survey | 1996 (March)-1998 (October) | Traditional practitioners (6), and inhabitants (37) | 43 | ND | Cultivated | Coelho-Ferreira, 2009 |
|  | Thailand | Decoction | Oral | ND | Diarrhea | Field survey | 1993-2011 | Traditional practitioners (14), and inhabitants (438) | 452 | ND | ND | Tangjitman et al., 2013 |
| Stem bark | India | Decoction | Oral | *Spondias mombin* or *Anacardium occidentale* and *Coffea arabica* | Diarrhea | Field survey | 2004 (September)-2005 (June), (5 months) | Inhabitants | 16 | ND | ND | Hajdu and Hohmann, 2012 |

**Legend:** NA: Not applicable; ND: Not documented

**TABLE S2**

**Table S2**: Pharmacological activities of *P*. *guajava* justifying its effect on diarrheal disorders

| **Model types** | **Cells/microbes/animals** | **Species used** | **Biological models** | **Part of plants** | **Preparation** | **Extracts** | **Dose** | **Results** | **Reference** |
| --- | --- | --- | --- | --- | --- | --- | --- | --- | --- |
| *ex vivo* | Animals | Guinea-pigs | Antidiarrheic effect (inhibition of continuously spontaneously contraction and electrically (coaxially) contraction in isolated ileum) | Leaf | Maceration (hot) | Petroleum ether/ methanol | 200 µg/mL - 1.6 mg/mL | *P*. *guajava* methanolic extract produced a reduction in tone contractions with a dose-dependent, and showed an inhibition of acetylcholine release in the coaxially stimulated ileum | (Lutterodt, 1989) |
| *ex vivo* | Animals | Guinea-pigs | Antidiarrheic effect (inhibition of ileum contraction) | Leaf/ stem bark | Decoction | Water | 80 µg/mL | *P*. *guajava* leaves and stem bark were highly active with at least an 80% inhibition of ACh or KCI-induced contractions | (Tona et al., 1999) |
| *ex vivo* | Animals | Guinea-pigs | Antidiarrheic effect (peristaltic reflex of isolated guinea pig ileum) | Leaf | Maceration | 70% methanol | ND | The peristaltic reflex of isolated guinea pig ileum was inhibited by the *P*. *guajava* extract | (Lozoya X et al., 1994) |
| *ex vivo* | Animals | Rabbit | Antidiarrheic effect (inhibit contraction in isolated jejunum) | Leaf | Maceration (hot) | Ethanol | 333.33, 666.66 and 1333.33 µg/mL | *P*. *guajava* extract inhibited acetylcholine-induced contractions, and terminating them completely at higher dose | (Ibeh et al., 2021) |
| *ex vivo* | Animals | Swiss-albino mice (male) | Antiparasitic activity (*Giardia lamblia* trophozoite count in the intestine) | Leaf | Maceration | 70% ethanol | 75 mg/kg/day | Mice treated with *P*. *guajava* extract showed significant *Giardia* trophozoite count reduction and the percentage reduction was 84.36% | (Khedr et al., 2021) |
| *in vitro* | Bacteria | 7 strains of *Escherichia coli* | Antibacterial (agar disk diffusion method, broth microdilution method) | Leaf | Crush/ maceration | Water/ 95% ethanol | 2.5 mg/disk | *P*. *guajava* aqueous extract produced inhibition zones against all strains of *Escherichia coli* (7 to 8 mm).  MIC were between 0.19 and 0.78 mg/mL for aqueous extract and 6.25 and 12.5 mg/mL for ethanolic extract. | (Voravuthikunchai et al., 2004) |
| *in vitro* | Bacteria | *Bacillus anthracis* | Antibacterial (agar well diffusion method) | Leaf | Crush | Water | 20 mg/well | No effect (the extract from *P*. *guajava* did not produce a zone of inhibition) | (Kaur et al., 2021) |
| *in vitro* | Bacteria | *Bacillus cereus*, *Enterococcus faecalis*, *Escherichia coli*, *Proteus vulgaris*, *Salmonella* Typhimurium, *Shigella flexneri*, *Staphylococcus aureus* | Antibacterial (broth microdilution method) | Leaf | Maceration (hot) | Dichloromethane: methanol (1:1) | ND | *P. guajava* extract combined with *Brachylaena transvaalensis* was mostly synergistic (mean ΣFIC value of 0.39). The combination with *Acanthospermum glabratum* also demonstrated a tendency to synergistic interactions with a mean (across all pathogens) ΣFIC value of 0.46 | (van Vuuren et al., 2015a) |
| *in vitro* | Bacteria | *Bacillus cereus, Escherichia coli, Salmonella* Enteritidis*, Staphylococcus aureus* | Antibacterial (agar disk diffusion method and broth microdilution method) | Leaf | Maceration | 70% ethanol | 50 mg/disk | *P*. *guajava* showed the ability to inhibit all tested bacteria growth (inhibition zone diameter ranging from 10 to 15 mm). The MIC of *P*. *guajava* against *E*. *coli*, *S*. *aureus* and *S*. Enteritidis were 625, 1250 and 625 µg/mL, respectively. | (Hemeg et al., 2020) |
| *in vitro* | Bacteria | *Bacillus subtilis*, *Pseudomonas aeruginosa*, *Escherichia coli* and *Staphylococcus aureus* | Antibacterial (agar disk diffusion method) | Fruit | Soaked | Hexane/ ethyl acetate/ methanol | 500 µg/disk | No effect | (McCook-Russell et al., 2012) |
| *in vitro* | Bacteria | *Bacillus subtilis, Staphylococcus aureus, Escherichia coli, Pseudomonas aeruginosa, Candida albicans* | Antibacterial (agar disk diffusion method) | Leaf/fruit | Maceration | 95% ethanol | 2 mg/disk | *P*. *guajava* extract showed activities against *B*. *subtilis*, *E*. *coli*, *P*. *aeruginosa* and *S*. *aureus* under UV light with a zone of inhibition between 8 and 12 mm | [(Cheeptham and Towers, 2002)](file:///C:\Users\liuch\AppData\Local\Netease\MailMaster\Users\liuch\AppData\Local\Netease\MailMaster\view\1\A827\review%20literature\pharmacology\pharmacology\Light-mediated%20activities%20of%20some%20Thai%20medicinal%20plant%20teas.pdf) |
| *in vitro* | Bacteria | *Clostridium perfringens*, *Enteropathogenic Escherichia coli* (EPEC) and *Salmonella enterica* | Antibacterial (broth microdilution method) | Leaf | Maceration/ infusion/ decoction | Water | NA | The infused and macerated extracts showed inhibition against three bacteria with MIC values of 1.25 mg/mL. The decoction showed antibacterial activities against *E*. *coli* EPEC (MIC = 2.5 mg/mL), *S*. *enterica* (MIC = 2.5 mg/mL) and *C*. *perfringens* (MIC = 1.25 mg/mL) | [(Chouegouong et al., 2023)](file:///C:\Users\liuch\AppData\Local\Netease\MailMaster\Users\liuch\AppData\Local\Netease\MailMaster\view\1\A827\review%20literature\pharmacology\pharmacology\Ethnopharmacological%20survey%20and%20antibacterial%20activity%20of%20medicinal%20plant%20extracts%20used%20against%20bacterial%20enteritis%20in%20rabbits.pdf) |
| *in vitro* | Bacteria | *Escherichia coli* | Antiadhesion effect of bacteria (hemagglutinating model with *Escherichia coli*) | Fruit | Maceration | Phosphate Buffer Solution | ND | Guava has a galactose-specific lectin that prevents adhesion of *E*. *coli* O157:H7 to red blood cells | (Coutiño-Rodríguez R et al., 2001) |
| *in vitro* | Bacteria | *Escherichia coli* and *Staphylococcus aureus* | Antibacterial (broth microdilution method) | Leaf/ stem bark | Maceration/decoction | 96% ethanol/ Water | NA | The ethanol extract showed antibacterial activity with *S*. *aureus* (MIC = 16 mg/mL) | (Bussmann et al., 2010) |
| *in vitro* | Bacteria | *Escherichia coli*, *Enterococcus faecalis*, *Staphylococcus aureus*, *Salmonella isangi*, *Salmonella* Typhimurium, *Shigella flexneri*, *Shigella sonnei* | Antibacterial (broth microdilution method) | Leaf | Soaked | Acetone | NA | Considerable antibacterial activities (MIC ranging from 0.156 mg/mL to 0.312 mg/mL after 24 h of incubation) | (Bisi-Johnson et al., 2017) |
| *in vitro* | Bacteria | *Escherichia coli, Escherichia paracoli, Citrobacter diversus, Klebsiella pneumoniae, Salmonella* Enteritidis*, Shigella flexneri, Staphylococcus aureus* and *Pseudomonas aeruginosa* | Antibacterial (broth microdilution method) | Leaf/ stem bark | Decoction | Water | NA | *P*. *guajava* leaves decoction showed antibacterial activities for each microorganism (15.62 < MIC < 62.5 µg/mL), and stem bark decoction show antibacterial activities with MIC = 125 or 250 µg/mL | (Tona et al., 1999) |
| *in vitro* | Bacteria | *Escherichia coli*, methicillin susceptible *Staphylococcus aureus* (MSSA), methicillin-resistant *Staphylococcus aureus* (MRSA), *Pseudomonas aeruginosa*, *Proteus vulgaris* and *Streptococcus pyogenes* | Antibacterial (agar well diffusion method, broth microdilution method) | Leaf | Soaked/blender | Chloroform/ 80% ethanol/ water | 5 mg/well | All of the extracts showed antibacterial inhibition (with inhibition zone diameter between 8 mm and 25 mm), the blender extract showed anti-MSSA inhibition (MIC = 3.1 mg/mL) | (Pesewu et al., 2008) |
| *in vitro* | Bacteria | *Escherichia coli*, *Pseudomonas aeruginosa* and *Staphylococcus aureus* | Antibacterial (broth microdilution method) | Leaf | Maceration | 70% ethanol | NA | *P*. *guajava* extract showed inhibitory activity against *S*. *aureus* only (MIC = 256 µg/mL) | (Morais-Braga et al., 2016) |
| *in vitro* | Bacteria | *Escherichia coli*, *Salmonella* spp. (13 isolates). and *Shigella* spp. (9 isolates). | Antibacterial (agar disk diffusion method) | Leaf | Maceration | Water/ methanol | 10 mg/disk | *P*. *guajava* leaves methanolic extract showed significant inhibitory activities against *Salmonella* spp. (two isolates), *Shigella* spp. (*S*. *flexneri, S*. *virchow* and *S*. *dysenteriae*) and *E*. *coli* (two isolates) (inhibitory zone ≥10 mm) | (Lin et al., 2002) |
| *in vitro* | Bacteria | *Escherichia coli, Salmonella* Typhi*, Shigella flexneri* | Antibacterial (agar disk diffusion method) | Leaf | Soaked | n-hexane/ acetone/ methanol | 50 mg/disk | *P*. *guajava* extracts showed activity against the three bacteria with inhibition zone ranging from 6 to 14mm, methanol and acetone extracts gave larger inhibition zones than n-hexane extract | (Cáceres et al., 1993) |
| *in vitro* | Bacteria | *Escherichia coli*, *Salmonella* Typhimurium, and *Staphylococcus aureus* | Antibacterial (broth microdilution method) | Leaf | Ultrasound | n-hexane, dichloromethane, ethyl-acetate fractions from ethanolic extract | NA | The aqueous and ethyl acetate fractions showed antibacterial activities with MIC values between 1.25 and 7.5 mg/mL | (Hall et al., 2023) |
| *in vitro* | Bacteria | *Escherichia coli, Staphylococcus aureus, Salmonella* and *Shigella* | Antibacterial (broth microdilution method) | Leaf/ fruit peel/ fruit flesh | Soaked (hot) | 70% ethanol | NA | White guava leaves and peel flavonoid inhibited the growth of *S*. *aureus* with MIC values of 0.313 mg/mL | (Zhang et al., 2018) |
| *in vitro* | Bacteria | *Proteus mirabilis, Pseudomonas aeruginosa, Staphylococcus aureus, Escherichia coli* | Antibacterial (agar well diffusion method) | Leaf | Maceration | Water | 200 mg/mL | *P*. *guajava* aqueous extract showed weak antibacterial inhibition against *P*. *mirabilis* (inhibition zone diameter < 15 mm) | (Adamu et al., 2005) |
| *in vitro* | Bacteria | *Salmonella* and *Shigella* (isolated strains) | Antibacterial (agar well diffusion method) | Leaf | Maceration/infusion | Ethanol/water (hot) | 25, 50, 100, 200 mg/mL | *P*. *guajava* ethanolic extract produced inhibition zone against *Shigella* with 22.0 mm at 200 mg/mL, 17 mm at 100 mg/mL, 10.3 mm at 50 mg/mL, 6.7 mm at 25 mg/mL. No effects were found on *Salmonella*, aqueous extract did not show any antibacterial activities | (Sule et al., 2022) |
| *in vitro* | Bacteria | *Salmonella* Typhi*, Salmonella* B, *Salmonella* D, three *Shigella flexneri* strains*,* two *Shigella dysenteriae* strains*,* two *Shigella boydii* strains *and Shigella sonnei* | Antibacterial (agar dilution streak method) | Leaf | Soaked | Ethanol | 1000 µg/mL | *P*. *guajava* showed antibacterial activities against *Salmonella* D, *S*. *flexneri* 2a, *S*. *flexneri* 4a and *S*. *dysenteriae* | (Maïkere-Faniyo et al., 1989) |
| *in vitro* | Bacteria | *Salmonella* Typhimurium | Acid resistance (exposure cells to guajava extract and then subjecting them to acidified media (pH 3.8)) | Branch/ fruit/ leaf | Soaked | Acetone/ ethanol/ methanol | 50 mg/mL | The ethanolic extract of guava fruit had greater effect in decreasing the acid resistance of *S*. *Typhimurium* | (Lim et al., 2013) |
| *in vitro* | Bacteria | *Shigella flexneri* and *Vibrio cholerae* | Antibacterial (broth microdilution method) | Leaf | Decoction | Water | NA | The decoction of *P*. *guajava* showed antibacterial activity towards *S*. *flexneri* (EC_50_ value 0.265 mg/mL) and *V*. *cholerae* (EC_50_ value 0.777 mg/mL) | (Birdi et al., 2010) |
| *in vitro* | Bacteria | *Staphylococcus aureus, Bacillus subtilis, Escherichia coli, Pasteurella multocida* | Antibacterial (agar disk diffusion method, broth microdilution method) | Leaf | Maceration | Ethyl acetate/ n-hexane/ chloroform/ methanol | NA | Methanolic extract of *P*. *guajava* possessed highest inhibitory potential against all tested strains with inhibited zone ranging from 21 to 25.66 mm. Chloroform extract of *P*. *guajava* demonstrated more sensitivity towards the growth of *S*. *aureus* with MIC value of 98 ± 4.05 μg/mL. Methanolic extract against *B*. *subtilis* (with MIC value of 115 ± 4.22 μg/mL), n-hexane and ethyl acetate against *E*. *coli* (with MIC values of 110 ± 1.1 and 108 ± 2 μg/mL, respectively). | (Afzal et al., 2019) |
| *in vitro* | Bacteria | *Staphylococcus aureus, Staphylococcus epidermidis, Enterococcus faecalis, Escherichia coli, Salmonella* Enteritidis*, Shigella flexneri, Klebisiella pneumoniae* and *methicillin-resistant Staphylococcus aureus (MRSA)* | Antibacterial (agar well diffusion method, broth microdilution method) | Leaf | Soaked | Water/ 70% acetone | 5 mg/well | Both extracts showed antibacterial activity against all tested strains, especially *S*. *aureus* and *S*. *flexneri* (inhibition zone diameter >17 mm), acetone showed a highest activity against *S*. *epidermidis* (MIC = 39 μg/mL) | (de Araújo et al., 2014) |
| *in vitro* | Bacteria | Two *Escherichia coli* strains and two *Staphylococcus aureus* strains | Antibacterial (radial diffusion in two layers of perforated agar (RDAP), and agar disk diffusion method) | Leaf | Decoction/ maceration (hot) | Water, diluted acetone and diluted ethanol (20%, 50%, 60% and 80% solvent in water) | ND | All concentrations of guava leaf extracts showed inhibitory effects on the growth of *E*. *coli* and *S*. *aureus* strains (with halos ranging from 15 to 32 mm) | (Vieira et al., 2001) |
| *in vitro* | Bacteria | *Vibrio cholerae* | Antibacterial (agar disk diffusion method) | Leaf | Decoction/ maceration | Water/ 50% ethanol | ND | *P*. *guajava* exhibited inhibitory activities | [(Shittu et al., 2016)](file:///C:\Users\liuch\AppData\Local\Netease\MailMaster\Users\liuch\AppData\Local\Netease\MailMaster\view\1\A827\review%20literature\pharmacology\pharmacology\Intestinal%20ameliorative%20effects%20of%20traditional%20Ogi-tutu,%20Vernonia%20amygdalina.pdf) |
| *in vitro* | Bacteria | *Vibrio parahaemolyticus, Escherichia coli, Pseudomonas aeruginosa, Staphylococcus aureus, Listeria monocytogenes* | Antibacterial (broth microdilution method) | Leaf | Ultrasound | 70% ethanol | NA | *P*. *guajava* leaf extract with and without chlorophyll removal showed antibacterial properties against all the selected bacteria, with MIC values ranging from 32 to 128 µg/mL | (Olatunde et al., 2021) |
| *in vitro* | Cell | *Labeo rohita* head-kidney macrophages | Anti-inflammatory (production of nitric oxide (NO), TNF-α and IL-β by blocking the NF-κB pathway in lipopolysaccharide (LPS)-induced *Labeo rohita* macrophages) | Leaf | Maceration | n-hexane: chloroform (1:1) and ethyl acetate fractions from 90% methanol extract | 50, 100, 200 µg/mL | *P*. *guajava* leaves ethyl acetate fraction inhibited the production of LPS-induced NO (75% inhibition at the highest dose), TNF-α and IL-1β in a dose-dependent manner. | (Sen et al., 2015) |
| *in vitro* | Cell | Macrophage (from female BALB/c mice) | Anti-inflammatory activity (cytokine secretion profiles) | Guava seed polysaccharide (GSPS) and its purified fractions GSF1, GSF2 and GSF3 | ND | ND | 8, 40 and 200 µg/mL | GSPS, GSF2 and GSF3 significantly (*p* < 0.05) increased IL-10 (an anti-inflammatory cytokine) cytokine secretions in LPS-stimulated peritoneal macrophages; GSPS, GSF1, GSF2 and GSF3 administrations significantly (*p* < 0.05) decreased IL-6 (a pro-inflammatory cytokine) and IL-6/IL-10 cytokine secretion ratios | (Lin and Lin, 2020) |
| *in vitro* | Cell | Murine fibrosarcoma L929sA | Anti-inflammatory (NF-κB-inhibitory activity) | Leaf | Maceration | Dichloromethane: methanol (1:1) | 62.5 µg/mL | A clear inhibition of NF-κB reporter gene expression can be observed with 1h pretreatment *P*. *guajava* extract | (Kaileh et al., 2007) |
| *in vitro* | Cell | RAW 264.7 macrophages | Anti-inflammatory (production of nitric oxide (NO)) | Leaf | Soaked | Ethanol | 500 μg/mL | *P*. *guajava* leaf extract inhibited LPS-induced NO production | (Perera et al., 2016) |
| *in vitro* | Cell and bacteria | Hep-2 (human laryngeal epithelial cell line), enteropathogenic *Escherichia coli* B170, enteroinvasive *Escherichia coli* E134 and *Shigella flexneri* | Antibacterial (effect on bacterial colonization) | Leaf | Decoction | Water | NA | The decoction of *P*. *guajava* decreased the adherence of *E*. *coli* B170 to Hep-2 cells, with EC_50_ value 0.1 mg/mL, and inhibited the invasion of *E*. *coli* E134 and *S*. *flexneri* to Hep-2 cells, with EC_50_ value 0.06 and 0.05 mg/mL | (Birdi et al., 2010) |
| *in vitro* | Parasite | *Entamoeba histolytica* | Antiamoebic test (dilution microplate method) | Leaf/ stem bark | Decoction | Water | NA | *P*. *guajava* stem bark and leaves decoctions inhibited the growth of the parasite with MAC ≤ 7.81 µg/mL, and MAC = 62.5 µg/mL respectively | (Tona et al., 1998, 1999, 2000) |
| *in vitro* | Parasite | *Entamoeba histolytica* | Antiamoebic test (dilution microplate method) | Leaf/ stem bark | Decoction | n-butanol, saponins and alkaloidic fractions from a water extract | NA | *P*. *guajava* stem bark butanol and saponins fractions inhibited the growth of the parasite with MAC = 2.6 and 8.3 µg/mL, respectively | (Tona et al., 2000) |
| *in vitro* | Parasite | *Giardia lamblia* | Antiparasitic activity | Leaf | Maceration/ percolation | 70% ethanol | NA | *P*. *guajava* macerated and percolated extracts showed activity against *G*. *lamblia* (IC_50_ = 457.91 ± 25.06 and 439.83 ± 24.11 μg/mL), | (Neiva et al., 2014) |
| *in vitro* | Parasite | *Giardia lamblia* | Antiparasitic activity (dilution microplate method) | Bark | Infusion | Water | 0.02, 0.313 and 2.5 mg/mL | *P*. *guajava* barks showed an anti-giardial activity with a dose dependent effect with 82.2% inhibition at a dose of 2.5 mg/mL | (Brandelli CL et al., 2009) |
| *in vitro* | Viruses | Simian (SA-11) and human (HCR3) rotaviruses | Antiviral activity (reduction of virus titers using TCID50 determinations) | Leaf | Decoction | Water | 8 μg/mL | *P*. *guajava* leaf extract showed activity against simian rotavirus with 93.8% inhibition and 47.5% inhibition of human (HCR3) viruses | (Gonçalves et al., 2005) |
| *in vitro* | Viruses | Simian rotavirus SA11 (propagated in MA-104 cells monolayers) | Antiviral activity (cytopathic effect of rotavirus on the treated MA-104 cells monolayers) | Leaf | Soaked | 95% ethanol | 50 and 500 µg/mL | Both doses of the extract did not show inhibition on cytopathic effect of rotavirus on the treated MA-104 cells monolayers | (Cecílio et al., 2012) |
| *in vivo* | Animals | Brown chicks (male) | Antidiarrheic effect (*E*. *coli* O78 infected chick (fecal bacterial shedding load of chicks)) | Leaf | Soaked | Water (extract)/ ethyl acetate (fraction) | 50 and 100 mg/kg | The bacterial shedding load of infected chick treated with extract at 50 mg/kg and 100 mg/kg significantly reduce at day 8 | (Geidam et al., 2015) |
| *in vivo* | Animals | Brown male chicks (*Escherichia coli* (O78)) | Antidiarrheic effect (*E*. *coli* O78 infected chick activity and body weight) | Leaf | Soaked | Water (extract)/ ethyl acetate (fraction) | 100 mg/kg | Appetence improve and depression reduce, improvement in body weight gain at day 6 | (Geidam et al., 2015) |
| *in vivo* | Animals | Brown male chicks (*Escherichia coli* (O78)) | Antidiarrheic effect (intestinal villous collapse with stunting, matting and fusion of villi) | Leaf | Soaked | Water (extract)/ ethyl acetate (fraction) | 100 mg/kg | Intestinal villi recovery to normalcy after treating with 100 mg/kg of ethyl acetate fraction of *P*. *guajava* for 7 days | (Geidam et al., 2015) |
| *in vivo* | Animals | KM mice | Antidiarrheic effect (diarrhea rates, diarrhea index, intestinal propulsion rate (determined by activated charcoal method)) | Leaf | Decoction/ soaked | Ethyl acetate and n-butanol fractions from water and 95% ethanol extracts | 2.5 g/kg | Inhibitory effects on diarrhea were observed after administration of water extract and n-butanol fractions of *P*. *guajava* at a dose of 2.5 g/kg | (Lu et al., 2020) |
| *in vivo* | Animals | Mice (adult, inoculated by *Vibrio cholerae*) | Anti-inflammatory (intestinal histopathological changes of *V*. *cholerae*-inoculated mice) | Leaf | Maceration | 50% ethanol | 250 mg/kg | *P*. *guajava* leaf extract showed anti-inflammatory activity at a dose of 250 mg/kg, i.e., the degeneration and necrosis of enterocytes, the inflammatory exudates in the lamina propria, hyperplasia of goblet cells and stumpy and club-shaped villi were reduced, while the regeneration of enterocytes were increased | (Shittu et al., 2016) |
| *in vivo* | Animals | Mice (female and male) | Antidiarrheic effect (castor oil test) | Leaf | Soaked | Ethanol | 750 mg/kg | *P*. *guajava* showed antidiarrheal activity | (Maïkere-Faniyo et al., 1989) |
| *in vivo* | Animals | Rattus norvegicus rats | Antidiarrheic effect (castor oil test, charcoal meal) | Leaf | Maceration | Water/ methanol | 400 mg/kg | *P*. *guajava* methanolic and aqueous extracts significantly reduced the weight of feces to 3.5 - 4 mg, compared to the average weight of feces in the control group (10.53 mg feces) and reduced the frequency of defecation. Movement of charcoal meal was reduced to 50-70% of the full intestine compared with 91-92% in the control group | (Lin et al., 2002) |
| *in vivo* | Animals | Rattus norvegicus rats | Antidiarrheic effect (fluid volume in the intestine) | Leaf | Maceration | Water/ methanol | 400 mg/kg | *P*. *guajava* leaves aqueous and methanolic extracts showed the ability to inhibit PGE_2_-induced enteropooling, with a reduction of the volume of intestinal fluid of 47% and 54%, respectively | (Lin et al., 2002) |
| *in vivo* | Animals | Sprague-Dawley rats (male) | Antidiarrheic effect (kidney weight, serum electrolytes and urinary volume and electrolytes) | Leaf | Maceration | 70% ethanol | 100 mg/kg body weight | Diarrheal rats treated with *P*. *guajava* leaf extract exhibited an increase in the total urine volume and electrolyte elimination, decrease of kidney weight and the reduce levels of serum electrolytes | (Koriem et al., 2019) |
| *in vivo* | Animals | Sprague-Dawley rats (male) | Antidiarrheic effect (microlax-induced experimental diarrhea) | Leaf | Soaked | Water | 0.2 mL/kg | A dose of 0.2 mL/kg fresh leaf extract produced 63.6% inhibition of propulsion | (Lutterodt, 1992) |
| *in vivo* | Animals | Swiss albino mice | Antidiarrheic effect (*Citrobacter rodentium* mouse model of infection: clearance of infection and bacterial load in the fecal sample) | Leaf | ND | 50% hydroalcoholic | 300 mg/kg/day | The mice treated with *P*. *guajava* leaf extract showed quicker clearance of infection and a flush out of the bacteria | (Gupta and Birdi, 2015) |
| *in vivo* | Animals | Swiss mice (male and female) | Anti-inflammatory (carrageenan-induced peritonitis) | Leaf | Soaked | Water/ 70% acetone | 50, 100 and 200 mg/kg | Both extracts significantly reduced the number of leukocyte from peritoneal exudates, which showed an inhibitory effect on cell recruitment into the peritoneal cavity | (de Araújo et al., 2014) |
| *in vivo* | Animals | Swiss mice (male) and Wistar rats (male) | Antidiarrheic effect (castor oil test) | Leaf | Maceration | Methanol | 50, 100 and 200 mg/kg | The number of wet feces produced by mice was reduced in a dose-dependent manner, as 60, 71 and 83 % of reduction compared to control group | (Olajide et al., 1999) |
| *in vivo* | Animals | Swiss mice (male) and Wistar rats (male) | Antidiarrheic effect (charcoal meal transit) | Leaf | Maceration | Methanol | 50, 100 and 200 mg/kg | *P*. *guajava* extract significantly reduced the charcoal meal transit in a dose-related manner, (29.9%, 25.4%, and 19%), compared to 58.7% for the negative control group and 23.4 % for the positive control group. | (Olajide et al., 1999) |
| *in vivo* | Animals | Swiss mice (male) and Wistar rats (male) | Anti-inflammatory (carrageenan-induced paw oedema) | Leaf | Maceration | Methanol | 50, 100 and 200 mg/kg | *P*. *guajava* extract exhibited anti-inflammatory activity, by inhibiting the oedema in a dose dependent manner (46%, 56% and 77%) | (Olajide et al., 1999) |
| *in vivo* | Animals | Weaned piglets | Antidiarrheic effect (enterotoxigenic *E*. *coli* (ETEC)-infected piglet (growth performance and diarrhea incidence in piglets)) | Leaf | Maceration | 95% ethanol | 50, 100 and 200 mg/kg | The supplementation of 50, 100 and 200 mg/kg of *P*. *guajava* extract in diets showed 14.29%, 8.93% and 7.14% diarrhea incidence reduction, respectively | (Wang et al., 2021) |
| *in vivo* | Animals | Weaned piglets | Antidiarrheic effect (intestinal mucosal damage, such as reduced villus height, villus loss) | Leaf | Maceration | 95% ethanol | 50, 100 and 200 mg/kg | Treatment with *P*. *guajava* extract increased villus height (*p* < 0.001) and villus height to crypt depth ratio (*p* <0.001), and decreased crypt depth (*p* < 0.001) in the jejunum compared with the negative group | (Wang et al., 2021) |
| *in vivo* | Animals | Weaned piglets | Anti-inflammatory (levels of TNF-α, IL-1β and IL-6 in the serum and jejunum mucosa) | Leaf | Maceration | 95% ethanol | 50, 100 and 200 mg/kg | Treatment with *P*. *guajava* extract decreased the level of TNF-α, IL-1β and IL-6 in the serum, and mRNA expression of TNF-α, IL-1β and IL-6 in the jejunum mucosa compared with the negative group | (Wang et al., 2021) |
| *in vivo* | Animals | Wistar rats | Antidiarrheic effect (enteropathogenic *Escherichia coli (*EPEC*)*-infected rat) | Leaf | Maceration | Ethanol | 200 and 400 mg/kg | *P*. *guajava* extract and quercetin showed a significant decline in the total number of diarrheal stools, weight of stools and mean defecation rate of stools after 6 and 24 h | (Hirudkar et al., 2020b) |
| *in vivo* | Animals | Wistar rats | Antidiarrheic effect (enteropathogenic *Escherichia coli (*EPEC*)*-infected rat, estimation of the level of EPEC in stools) | Leaf | Maceration | Ethanol | 200 mg/kg | *P*. *guajava* extract and quercetin showed a significant decline the level of EPEC in stools after 4 h of treatment | (Hirudkar et al., 2020b) |
| *in vivo* | Animals | Wistar rats | Antidiarrheic effect *(Shigella flexneri*-induced rat: stool content) | Leaf | Maceration | Ethanol | 100, 200 and 400 mg/kg | *P*. *guajava* extract treatment demonstrated a significant decline in total number of diarrheal stools, weight of stools and water content of stools after 1st, 3rd and 5th day after diarrhea induction | (Hirudkar et al., 2020a) |
| *in vivo* | Animals | Wistar rats | Antidiarrheic effect (*Shigella flexneri*-induced rat: level of *S*. *flexneri* in stools) | Leaf | Maceration | Ethanol | 200 mg/kg | *P*. *guajava* extract demonstrated a significant reduction in the density of *S*. *flexneri* in stools of rats after 3rd day | (Hirudkar et al., 2020a) |
| *in vivo* | Animals | Wistar rats | Anti-inflammatory (production of NO and levels of IL-1β, and TNF-α) | Leaf | Maceration | Ethanol | 200 mg/kg | EPEC-infected rats treated with *P*. *guajava* extract showed a significant (*p* < 0.05) decline in the production of NO and expression of IL-1β and TNF-α | (Hirudkar et al., 2020b) |
| *in vivo* | Animals | Wistar rats | Anti-inflammatory (*Shigella flexneri*-induced rat: level of NO, IL-1β and TNF-α) | Leaf | Maceration | Ethanol | 200 mg/kg | *P*. *guajava* extract and quercetin significantly reduced the production of NO and the expression of IL-6 and TNF-α | (Hirudkar et al., 2020a) |
| *in vivo* | Animals | Wistar rats (adult) | Antidiarrheic effect (charcoal meal transit) | Leaf | Maceration (hot) | Ethanol | 200, 400 and 800 mg/kg | *P*. *guajava* extract showed a dose-dependent inhibition of intestinal motility with 14.82% inhibition at 200 mg/kg, 16.87% inhibition at 400 mg/kg and 33.59% inhibition at 8000 mg/kg | (Ibeh et al., 2021) |
| *in vivo* | Animals | Wistar rats (duodenum, ileum, colon) | Antidiarrheic effect (intestinal transport of water, gastrointestinal propulsion) | Leaf | Infusion | Water | ND | *P*. *guajava* extract increased water absorption in the colon with water flow values of -8.3 ± 4.7 µL/g/h, and reduced gastrointestinal propulsion with 0.45 fractional value, while the control group was 0.72 | (Almeida et al., 1995) |
| *in vivo* | Animals | Wister rats (male) | Antidiarrheic effect (castor oil test) | Leaf | ND | 98% ethanol | 250, 500 and 750 mg/kg | *P*. *guajava* leaf extract inhibited the frequency of defecation (with10%, 26% and 52% inhibition, respectively) and wetted feces (with 16%, 43% and 63% inhibition, respectively) when compared to the control group | (Mazumdar et al., 2015) |
| *in vivo* | Animals | Wister rats (male) | Antidiarrheic effect (gastrointestinal motility test with BaSO4 milk (BSM) model) | Leaf | ND | 98% ethanol | 250, 500 and 750 mg/kg | Gastrointestinal motility of rats in BSM model decrease at 30 min study and percentages of inhibition are 37.83%, 30.01% and 29.56% at doses of 750, 500 and 250 mg/kg, respectively, while positive control exhibits 39.60% inhibition | (Mazumdar et al., 2015) |

Legend: NA: Not applicable; ND: Not documented; Maceration: soaking in all kinds of solvents more than 24h; Decoction: boiling the plant material with water; Soaked: soaking in all kinds of solvents less than 24h (including overnight), percolation and reflux; Infusion: soaking in hot water (below the boiling point). A Slash “ / ” corresponds to the term “and”.

**REFERENCES**

**References:**

Abe, R., and Ohtani, K. (2013). An ethnobotanical study of medicinal plants and traditional therapies on Batan Island, the Philippines. *J. Ethnopharmacol.* 145, 554–565.

Adamu, H. M., Abayeh, O. J., Agho, M. O., Abdullahi, A. L., Uba, A., Dukku, H. U., et al. (2005). An ethnobotanical survey of Bauchi State herbal plants and their antimicrobial activity. *J. Ethnopharmacol.* 99, 1–4.

Afzal, M., Iqbal, R., Mahmood, Z., Zeshan, B., and Wattoo, J. (2019). Study of GC-MS and HPLC characterized metabolic compounds in guava (Psidium guajava L.) leaves. *Pak. J. Agric. Sci.* 56, 709–713.

Ahmed, N., Mahmood, A., Mahmood, A., Tahir, S. S., Bano, A., Malik, R. N., et al. (2014). Relative importance of indigenous medicinal plants from Layyah district, Punjab Province, Pakistan. *J. Ethnopharmacol.* 155, 509–523.

Allabi, A. C., Busia, K., Ekanmian, V., and Bakiono, F. (2011). The use of medicinal plants in self-care in the Agonlin region of Benin. *J. Ethnopharmacol.* 133, 234–243.

Almeida, B. V., Ribeiro, D. A., Santos, M. O., de Macêdo, D. G., Macedo, J. G. F., Macêdo, M. J. F., et al. (2022). Mixtures of medicinal plants from caatinga: Basis for further bioprospecting studies. *South Afr. J. Bot.* Available at: https://www.sciencedirect.com/science/article/pii/S0254629921005433 (Accessed January 1, 1AD).

Almeida, C., Karnikowski, M., Foleto, R., and Baldisserotto, B. (1995). Analysis of antidiarrhoeic effect of plants used in popular medicine. *Rev. Saude Publica* 29, 428–433.

Alonso-Castro, A. J., Domínguez, F., Maldonado-Miranda, J. J., Castillo-Pérez, L. J., Carranza-Álvarez, C., Solano, E., et al. (2017). Use of medicinal plants by health professionals in Mexico. *J. Ethnopharmacol.* 198, 81–86.

Alonso-Castro, A. J., Jose Maldonado-Miranda, J., Zarate-Martinez, A., Jacobo-Salcedo, M. del R., Fernández-Galicia, C., Alejandro Figueroa-Zuñiga, L., et al. (2012). Medicinal plants used in the Huasteca Potosina, México. *J. Ethnopharmacol.* 143, 292–298. doi: 10.1016/j.jep.2012.06.035

Amusan, O. O. G., Dlamini, P. S., Msonthi, J. D., and Makhubu, L. P. (2002). Some herbal remedies from Manzini region of Swaziland. *J. Ethnopharmacol.* 79, 109–112. doi: 10.1016/S0378-8741(01)00381-6

Andrade-Cetto, A. (2009). Ethnobotanical study of the medicinal plants from Tlanchinol, Hidalgo, México. *J. Ethnopharmacol.* 122, 163–171. doi: 10.1016/j.jep.2008.12.008

Arnason, T., Uck, F., Lambert, J., and Hebda, R. (1980). Maya medicinal plants of San Jose Succotz, Belize. *J. Ethnopharmacol.* 2, 345–364. doi: 10.1016/S0378-8741(80)81016-6

Au, D. T., Wu, J., Jiang, Z., Chen, H., Lu, G., and Zhao, Z. (2008). Ethnobotanical study of medicinal plants used by Hakka in Guangdong, China. *J. Ethnopharmacol.* 117, 41–50. doi: 10.1016/j.jep.2008.01.016

Baldé, A. M., Traoré, M. S., Baldé, M. A., Barry, M. S., Diallo, A., Camara, M., et al. (2016). Ethnomedical and ethnobotanical investigations on the response capacities of Guinean traditional health practioners in the management of outbreaks of infectious diseases: The case of the Ebola virus epidemic. *J. Ethnopharmacol.* 182, 137–149. doi: 10.1016/j.jep.2016.02.021

Beverly, C. D., and Sudarsanam, G. (2011). Ethnomedicinal plant knowledge and practice of people of Javadhu hills in Tamilnadu. *Asian Pac. J. Trop. Biomed.* 1, 79–81. doi: 10.1016/S2221-1691(11)60129-9

Bieski, I. G. C., Leonti, M., Arnason, J. T., Ferrier, J., Rapinski, M., Violante, I. M. P., et al. (2015). Ethnobotanical study of medicinal plants by population of Valley of Juruena Region, Legal Amazon, Mato Grosso, Brazil. *J. Ethnopharmacol.* 173, 383–423. doi: 10.1016/j.jep.2015.07.025

Birdi, T., Daswani, P., Brijesh, S., Tetali, P., Natu, A., and Antia, N. (2010). Newer insights into the mechanism of action of Psidium guajava L. leaves in infectious diarrhoea. *BMC Complement. Altern. Med.* 10, 33. doi: 10.1186/1472-6882-10-33

Bisi-Johnson, M. A., Obi, C. L., Samuel, B. B., Eloff, J. N., and Okoh, A. I. (2017). Antibacterial activity of crude extracts of some South African medicinal plants against multidrug resistant etiological agents of diarrhoea. *BMC Complement. Altern. Med.* 17, 321. doi: 10.1186/s12906-017-1802-4

Boulogne, I., Germosén-Robineau, L., Ozier-Lafontaine, H., Fleury, M., and Loranger-Merciris, G. (2011). TRAMIL ethnopharmalogical survey in Les Saintes (Guadeloupe, French West Indies): A comparative study. *J. Ethnopharmacol.* 133, 1039–1050. doi: 10.1016/j.jep.2010.11.034

Bourdy, G., DeWalt, S. J., Chávez de Michel, L. R., Roca, A., Deharo, E., Muñoz, V., et al. (2000). Medicinal plants uses of the Tacana, an Amazonian Bolivian ethnic group. *J. Ethnopharmacol.* 70, 87–109. doi: 10.1016/S0378-8741(99)00158-0

Bradacs, G., Heilmann, J., and Weckerle, C. S. (2011). Medicinal plant use in Vanuatu: A comparative ethnobotanical study of three islands. *J. Ethnopharmacol.* 137, 434–448. doi: 10.1016/j.jep.2011.05.050

Brandelli CL, Giordani RB, De Carli GA, and Tasca T (2009). Indigenous traditional medicine: in vitro anti-giardial activity of plants used in the treatment of diarrhea. *Parasitol. Res.* 104, 1345–9. doi: 10.1007/s00436-009-1330-3

Bussmann, R. W., Malca-García, G., Glenn, A., Sharon, D., Chait, G., Díaz, D., et al. (2010). Minimum inhibitory concentrations of medicinal plants used in Northern Peru as antibacterial remedies. *J. Ethnopharmacol.* 132, 101–108. doi: 10.1016/j.jep.2010.07.048

Caballero-Serrano, V., McLaren, B., Carrasco, J. C., Alday, J. G., Fiallos, L., Amigo, J., et al. (2019). Traditional ecological knowledge and medicinal plant diversity in Ecuadorian Amazon home gardens. *Glob. Ecol. Conserv.* 17, e00524. doi: 10.1016/j.gecco.2019.e00524

Cáceres, A., Fletes, L., Aguilar, L., Ramirez, O., Figueroa, L., Taracena, A. M., et al. (1993). Plants used in Guatemala for the treatment of gastrointestinal disorders. 3. Confirmation of activity against enterobacteria of 16 plants. *J. Ethnopharmacol.* 38, 31–38. doi: 10.1016/0378-8741(93)90076-H

Canales, M., Hernández, T., Caballero, J., Vivar, A. R. de, Avila, G., Duran, A., et al. (2005). Informant consensus factor and antibacterial activity of the medicinal plants used by the people of San Rafael Coxcatlán, Puebla, México. *J. Ethnopharmacol.* 97, 429–439. doi: 10.1016/j.jep.2004.11.013

Cano, J. H., and Volpato, G. (2004). Herbal mixtures in the traditional medicine of Eastern Cuba. *J. Ethnopharmacol.* 90, 293–316. doi: 10.1016/j.jep.2003.10.012

Cartaxo, S. L., de Almeida Souza, M. M., and de Albuquerque, U. P. (2010). Medicinal plants with bioprospecting potential used in semi-arid northeastern Brazil. *J. Ethnopharmacol.* 131, 326–342. doi: 10.1016/j.jep.2010.07.003

Cecílio, A. B., Faria, D. B. de, Oliveira, P. de C., Caldas, S., Oliveira, D. A. de, Sobral, M. E. G., et al. (2012). Screening of Brazilian medicinal plants for antiviral activity against rotavirus. *J. Ethnopharmacol.* 141, 975–981. doi: 10.1016/j.jep.2012.03.031

Ceuterick, M., Vandebroek, I., Torry, B., and Pieroni, A. (2008). Cross-cultural adaptation in urban ethnobotany: The Colombian folk pharmacopoeia in London. *J. Ethnopharmacol.* 120, 342–359. doi: 10.1016/j.jep.2008.09.004

Chassagne, F., Butaud JF, Torrente F, Conte E, Ho R, and Raharivelomanana P (2022). Polynesian medicine used to treat diarrhea and ciguatera: An ethnobotanical survey in six islands from French Polynesia. *J. Ethnopharmacol.* 292, 115186. doi: 10.1016/j.jep.2022.115186

Chassagne, F., Hul, S., Deharo, E., and Bourdy, G. (2016). Natural remedies used by Bunong people in Mondulkiri province (Northeast Cambodia) with special reference to the treatment of 11 most common ailments. *J. Ethnopharmacol.* 191, 41–70. doi: 10.1016/j.jep.2016.06.003

Cheeptham, N., and Towers, G. H. N. (2002). Light-mediated activities of some Thai medicinal plant teas. *Fitoterapia* 73, 651–662. doi: 10.1016/S0367-326X(02)00224-1

Chouegouong, M. T., Majoumouo, M. S., Menkem, E. Z., Yimgang, L. V., Toghueo, R. M. K., Etchu, K. A., et al. (2023). Ethnopharmacological survey and antibacterial activity of medicinal plant extracts used against bacterial enteritis in rabbits. *Adv. Tradit. Med.* 23, 213–223. doi: 10.1007/s13596-021-00615-1

Coelho-Ferreira, M. (2009). Medicinal knowledge and plant utilization in an Amazonian coastal community of Marudá, Pará State (Brazil). *J. Ethnopharmacol.* 126, 159–175. doi: 10.1016/j.jep.2009.07.016

Coutiño-Rodríguez R, Hernández-Cruz P, and Giles-Ríos H (2001). Lectins in fruits having gastrointestinal activity: their participation in the hemagglutinating property of Escherichia coli O157:H7. *Arch. Med. Res.* 32, 251–7. doi: 10.1016/s0188-4409(01)00287-9

Cox, P. A. (1993). Saving the ethnopharmacological heritage of Samoa. *J. Ethnopharmacol.* 38, 177–180. doi: 10.1016/0378-8741(93)90014-V

Dassekpo, I. S., Achigan-Dako, E. G., Tenté, B., Houssou, C. S., and Ahanchédé, A. (2020). Valuation of Newbouldia laevis and its endogenous conservation in Benin (West Africa). *J. Herb. Med.* 23, 100388. doi: 10.1016/j.hermed.2020.100388

de Araújo, A. A., Soares, L. A. L., Assunção Ferreira, M. R., de Souza Neto, M. A., da Silva, G. R., de Araújo, R. F., et al. (2014). Quantification of polyphenols and evaluation of antimicrobial, analgesic and anti-inflammatory activities of aqueous and acetone–water extracts of Libidibia ferrea, Parapiptadenia rigida and Psidium guajava. *J. Ethnopharmacol.* 156, 88–96. doi: 10.1016/j.jep.2014.07.031

de Boer, H. J., Lamxay, V., and Björk, L. (2012). Comparing medicinal plant knowledge using similarity indices: A case of the Brou, Saek and Kry in Lao PDR. *J. Ethnopharmacol.* 141, 481–500. doi: 10.1016/j.jep.2012.03.017

de Santana, B. F., Voeks, R. A., and Funch, L. S. (2016). Ethnomedicinal survey of a maroon community in Brazil’s Atlantic tropical forest. *J. Ethnopharmacol.* 181, 37–49. doi: 10.1016/j.jep.2016.01.014

de Wet, H., Nkwanyana MN, and van Vuuren SF (2010). Medicinal plants used for the treatment of diarrhoea in northern Maputaland, KwaZulu-Natal Province, South Africa. *J. Ethnopharmacol.* 130, 284–9. doi: 10.1016/j.jep.2010.05.004

Deb, L., Laishram, S., Khumukcham, N., Ningthoukhongjam, D., Nameirakpam, S. S., Dey, A., et al. (2015). Past, present and perspectives of Manipur traditional medicine: A major health care system available for rural population in the North-East India. *J. Ethnopharmacol.* 169, 387–400. doi: 10.1016/j.jep.2014.12.074

Desmarchelier, C., Gurni, A., Ciccia, G., and Giulietti, A. M. (1996). Ritual and medicinal plants of the Ese’ejas of the Amazonian rainforest (Madre de Dios, Perú). *J. Ethnopharmacol.* 52, 45–51. doi: 10.1016/0378-8741(96)01390-6

Di Stasi, L. C., Oliveira, G. P., Carvalhaes, M. A., Queiroz-Junior, M., Tien, O. S., Kakinami, S. H., et al. (2002). Medicinal plants popularly used in the Brazilian Tropical Atlantic Forest. *Fitoterapia* 73, 69–91. doi: 10.1016/S0367-326X(01)00362-8

do Nascimento Magalhães, K., Guarniz, W. A. S., Sá, K. M., Freire, A. B., Monteiro, M. P., Nojosa, R. T., et al. (2019). Medicinal plants of the Caatinga, northeastern Brazil: Ethnopharmacopeia (1980–1990) of the late professor Francisco José de Abreu Matos. *J. Ethnopharmacol.* 237, 314–353. doi: 10.1016/j.jep.2019.03.032

Elliott, S., and Brimacombe, J. (1987). The medicinal plants of Gunung Leuser National Park, Indonesia. *J. Ethnopharmacol.* 19, 285–317. doi: 10.1016/0378-8741(87)90006-7

Frei, B., Baltisberger, M., Sticher, O., and Heinrich, M. (1998). Medical ethnobotany of the Zapotecs of the Isthmus-Sierra (Oaxaca, Mexico): Documentation and assessment of indigenous uses. *J. Ethnopharmacol.* 62, 149–165. doi: 10.1016/S0378-8741(98)00051-8

Geidam, Y. A., Ambali, A. G., Onyeyili, P. A., Tijjani, M. B., Gambo, H. I., and Gulani, I. A. (2015). Antibacterial efficacy of ethyl acetate fraction of Psidium guajava leaf aqueous extract on experimental Escherichia coli (O78) infection in chickens. *Vet. World* 8, 358–362. doi: 10.14202/vetworld.2015.358-362

Geissler, P. W., Harris, S. A., Prince, R. J., Olsen, A., Odhiambo, R. A., Oketch-Rabah, H., et al. (2002). Medicinal plants used by Luo mothers and children in Bondo district, Kenya. *J. Ethnopharmacol.* 83, 39–54. doi: 10.1016/S0378-8741(02)00191-5

Ghimire, K., and Bastakoti, R. R. (2009). Ethnomedicinal knowledge and healthcare practices among the Tharus of Nawalparasi district in central Nepal. *For. Ecol. Manag.* 257, 2066–2072. doi: 10.1016/j.foreco.2009.01.039

Ghorbani, A., Langenberger, G., Feng, L., and Sauerborn, J. (2011). Ethnobotanical study of medicinal plants utilised by Hani ethnicity in Naban River Watershed National Nature Reserve, Yunnan, China. *J. Ethnopharmacol.* 134, 651–667. doi: 10.1016/j.jep.2011.01.011

Giovannini, P. (2015). Medicinal plants of the Achuar (Jivaro) of Amazonian Ecuador: Ethnobotanical survey and comparison with other Amazonian pharmacopoeias. *J. Ethnopharmacol.* 164, 78–88. doi: 10.1016/j.jep.2015.01.038

Giovannini, P., and Heinrich, M. (2009). Xki yoma’ (our medicine) and xki tienda (patent medicine)—Interface between traditional and modern medicine among the Mazatecs of Oaxaca, Mexico. *J. Ethnopharmacol.* 121, 383–399. doi: 10.1016/j.jep.2008.11.003

Gonçalves, J. L. S., Lopes, R. C., Oliveira, D. B., Costa, S. S., Miranda, M. M. F. S., Romanos, M. T. V., et al. (2005). In vitro anti-rotavirus activity of some medicinal plants used in Brazil against diarrhea. *J. Ethnopharmacol.* 99, 403–407. doi: 10.1016/j.jep.2005.01.032

Grosvenor, P. W., Gothard, P. K., McWilliam, N. C., Supriono, A., and Gray, D. O. (1995). Medicinal plants from Riau Province, Sumatra, Indonesia. Part 1: Uses. *J. Ethnopharmacol.* 45, 75–95. doi: 10.1016/0378-8741(94)01209-I

Gumisiriza, H., Birungi, G., Olet, E. A., and Sesaazi, C. D. (2019). Medicinal plant species used by local communities around Queen Elizabeth National Park, Maramagambo Central Forest Reserve and Ihimbo Central Forest Reserve, South western Uganda. *J. Ethnopharmacol.* 239, 111926. doi: 10.1016/j.jep.2019.111926

Gupta, M. P., Solís, P. N., Calderón, A. I., Guinneau-Sinclair, F., Correa, M., Galdames, C., et al. (2005). Medical Ethnobotany of the Teribes of Bocas del Toro, Panama. *J. Ethnopharmacol.* 96, 389–401. doi: 10.1016/j.jep.2004.08.032

Gupta, P., and Birdi, T. (2015). Psidium guajava leaf extract prevents intestinal colonization of Citrobacter rodentium in the mouse model. *J. Ayurveda Integr. Med.* 6, 50–52. doi: 10.4103/0975-9476.146557

Hajdu, Z., and Hohmann, J. (2012). An ethnopharmacological survey of the traditional medicine utilized in the community of Porvenir, Bajo Paraguá Indian Reservation, Bolivia. *J. Ethnopharmacol.* 139, 838–857. doi: 10.1016/j.jep.2011.12.029

Hall, A. M., Baskiyar, S., Heck, K. L., Hayden, M. D., Ren, C., Nguyen, C., et al. (2023). Investigation of the chemical composition of antibacterial Psidium guajava extract and partitions against foodborne pathogens. *Food Chem.* 403, 134400. doi: 10.1016/j.foodchem.2022.134400

Heinrich, M., Ankli, A., Frei, B., Weimann, C., and Sticher, O. (1998). Medicinal plants in Mexico: healers’ consensus and cultural importance. *Soc. Sci. Med.* 47, 1859–1871. doi: 10.1016/S0277-9536(98)00181-6

Heinrich, M., Rimpler, H., and Barrera, N. A. (1992). Indigenous phytotherapy of gastrointestinal disorders in a lowland Mixe community (Oaxaca, Mexico): Ethnopharmacologic evaluation. *J. Ethnopharmacol.* 36, 63–80. doi: 10.1016/0378-8741(92)90062-V

Hemeg, H. A., Moussa, I. M., Ibrahim, S., Dawoud, T. M., Alhaji, J. H., Mubarak, A. S., et al. (2020). Antimicrobial effect of different herbal plant extracts against different microbial population. *Saudi J. Biol. Sci.* 27, 3221–3227. doi: 10.1016/j.sjbs.2020.08.015

Hernández, T., Canales, M., Avila, J. G., Duran, A., Caballero, J., Vivar, A. R. de, et al. (2003). Ethnobotany and antibacterial activity of some plants used in traditional medicine of Zapotitlán de las Salinas, Puebla (México). *J. Ethnopharmacol.* 88, 181–188. doi: 10.1016/S0378-8741(03)00213-7

Hirudkar, J. R., Parmar, K. M., Prasad, R. S., Sinha, S. K., Jogi, M. S., Itankar, P. R., et al. (2020a). Quercetin a major biomarker of Psidium guajava L. inhibits SepA protease activity of Shigella flexneri in treatment of infectious diarrhoea. *Microb. Pathog.* 138, 103807. doi: 10.1016/j.micpath.2019.103807

Hirudkar, J. R., Parmar, K. M., Prasad, R. S., Sinha, S. K., Lomte, A. D., Itankar, P. R., et al. (2020b). The antidiarrhoeal evaluation of Psidium guajava L. against enteropathogenic Escherichia coli induced infectious diarrhoea. *J. Ethnopharmacol.* 251, 112561. doi: 10.1016/j.jep.2020.112561

Hulley, I. M., and Van Wyk, B.-E. (2019). Quantitative medicinal ethnobotany of Kannaland (western Little Karoo, South Africa): Non-homogeneity amongst villages. *South Afr. J. Bot.* 122, 225–265. doi: 10.1016/j.sajb.2018.03.014

Ibeh, L., Ijioma, S., Emmanuel, O., Timothy, C., and Ugbogu, E. (2021). Psidium guajava leaf extract improves gastrointestinal functions in rats and rabbits: an implication for ulcer and diarrhoea management. *Biomarkers* 26, 737–746. doi: 10.1080/1354750X.2021.1992651

Inta, A., Shengji, P., Balslev, H., Wangpakapattanawong, P., and Trisonthi, C. (2008). A comparative study on medicinal plants used in Akha’s traditional medicine in China and Thailand, cultural coherence or ecological divergence? *J. Ethnopharmacol.* 116, 508–517. doi: 10.1016/j.jep.2007.12.015

Jamir, K., Seshagirirao, K., and Meitei, M. D. (2022). Indigenous oral knowledge of wild medicinal plants from the Peren district of Nagaland, India in the Indo Burma hot-spot. *Acta Ecol. Sin.* 42, 206–223. doi: 10.1016/j.chnaes.2021.04.001

Jaradat, N., Ayesh, O., and Anderson, C. (2016). Ethnopharmacological survey about medicinal plants utilized by herbalists and traditional practitioner healers for treatments of diarrhea in the West Bank/Palestine. *J. Ethnopharmacol.* 182, 57–66. doi: 10.1016/j.jep.2016.02.013

Juárez-Vázquez, M. del C., Carranza-Álvarez, C., Alonso-Castro, A. J., González-Alcaraz, V. F., Bravo-Acevedo, E., Chamarro-Tinajero, F. J., et al. (2013). Ethnobotany of medicinal plants used in Xalpatlahuac, Guerrero, México. *J. Ethnopharmacol.* 148, 521–527. doi: 10.1016/j.jep.2013.04.048

Kadir, M. F., Bin Sayeed, M. S., and Mia, M. M. K. (2013). Ethnopharmacological survey of medicinal plants used by traditional healers in Bangladesh for gastrointestinal disorders. *J. Ethnopharmacol.* 147, 148–156. doi: 10.1016/j.jep.2013.02.023

Kaileh, M., Berghe, W. V., Boone, E., Essawi, T., and Haegeman, G. (2007). Screening of indigenous Palestinian medicinal plants for potential anti-inflammatory and cytotoxic activity. *J. Ethnopharmacol.* 113, 510–516. doi: 10.1016/j.jep.2007.07.008

Kaur, R., Tiwari, A., Manish, M., Maurya, I. K., Bhatnagar, R., and Singh, S. (2021). Common garlic (Allium sativum L.) has potent Anti-Bacillus anthracis activity. *J. Ethnopharmacol.* 264, 113230. doi: 10.1016/j.jep.2020.113230

Khedr, S. I., Mokhamer, E. H. M., Hassan, A. A. A., El-Feki, A. S., Elkhodary, G. M., and El-Gerbed, M. S. A. (2021). Psidium guajava Linn leaf ethanolic extract: In vivo giardicidal potential with ultrastructural damage, anti-inflammatory and antioxidant effects. *Saudi J. Biol. Sci.* 28, 427–439. doi: 10.1016/j.sjbs.2020.10.026

Kichu, M., Malewska, T., Akter, K., Imchen, I., Harrington, D., Kohen, J., et al. (2015). An ethnobotanical study of medicinal plants of Chungtia village, Nagaland, India. *J. Ethnopharmacol.* 166, 5–17. doi: 10.1016/j.jep.2015.02.053

Koriem, K. M. M., Arbid, M. S., and Saleh, H. N. (2019). Antidiarrheal and protein conservative activities of Psidium guajava in diarrheal rats. *J. Integr. Med.* 17, 57–65. doi: 10.1016/j.joim.2018.12.001

Kujawska, M., and Hilgert, N. I. (2014). Phytotherapy of Polish migrants in Misiones, Argentina: Legacy and acquired plant species. *J. Ethnopharmacol.* 153, 810–830. doi: 10.1016/j.jep.2014.03.044

Kujawska, M., and Schmeda-Hirschmann, G. (2022). The use of medicinal plants by Paraguayan migrants in the Atlantic Forest of Misiones, Argentina, is based on Guaraní tradition, colonial and current plant knowledge. *J. Ethnopharmacol.* 283, 114702. doi: 10.1016/j.jep.2021.114702

Lans, C., and Brown, G. (1998). Ethnoveterinary medicines used for ruminants in Trinidad and Tobago. *Prev. Vet. Med.* 35, 149–163. doi: 10.1016/S0167-5877(98)00066-X

Lans, C., Harper, T., Georges, K., and Bridgewater, E. (2000). Medicinal plants used for dogs in Trinidad and Tobago. *Prev. Vet. Med.* 45, 201–220. doi: 10.1016/S0167-5877(00)00123-9

Lee, C., Kim, S.-Y., Eum, S., Paik, J.-H., Bach, T. T., Darshetkar, A. M., et al. (2019). Ethnobotanical study on medicinal plants used by local Van Kieu ethnic people of Bac Huong Hoa nature reserve, Vietnam. *J. Ethnopharmacol.* 231, 283–294. doi: 10.1016/j.jep.2018.11.006

Leonti, M., Sticher, O., and Heinrich, M. (2002). Medicinal plants of the Popoluca, México: organoleptic properties as indigenous selection criteria. *J. Ethnopharmacol.* 81, 307–315. doi: 10.1016/S0378-8741(02)00078-8

Li, D., and Xing, F. (2016). Ethnobotanical study on medicinal plants used by local Hoklos people on Hainan Island, China. *J. Ethnopharmacol.* 194, 358–368. doi: 10.1016/j.jep.2016.07.050

Lim, S.-W., Kim, S.-W., Lee, S.-C., and Yuk, H.-G. (2013). Exposure of Salmonella Typhimurium to guava extracts increases their sensitivity to acidic environments. *Food Control* 33, 393–398. doi: 10.1016/j.foodcont.2013.03.033

Lin, H.-C., and Lin, J.-Y. (2020). Characterization of guava (Psidium guajava Linn) seed polysaccharides with an immunomodulatory activity. *Int. J. Biol. Macromol.* 154, 511–520. doi: 10.1016/j.ijbiomac.2020.03.137

Lin, J., Puckree, T., and Mvelase, T. (2002). Anti-diarrhoeal evaluation of some medicinal plants used by Zulu traditional healers. *J. Ethnopharmacol.* 79, 53–56. doi: 10.1016/S0378-8741(01)00353-1

Longuefosse, J.-L., and Nossin, E. (1996). Medical ethnobotany survey in Martinique. *J. Ethnopharmacol.* 53, 117–142. doi: 10.1016/0378-8741(96)01425-0

Lozoya X, Meckes M, Abou-Zaid M, Tortoriello J, Nozzolillo C, and Arnason JT (1994). Quercetin glycosides in Psidium guajava L. leaves and determination of a spasmolytic principle. *Arch. Med. Res.* 25, 11–5. Available at: https://pubmed.ncbi.nlm.nih.gov/8019108/

Lu, Mao D, Li X, Ma Y, Luan Y, Cao Y, et al. (2020). Changes of intestinal microflora diversity in diarrhea model of KM mice and effects of Psidium guajava L. as the treatment agent for diarrhea. *J. Infect. Public Health* 13, 16–26. doi: 10.1016/j.jiph.2019.04.015

Lutterodt, G. D. (1989). Inhibition of gastrointestinal release of acetylcholine by quercetin as a possible mode of action of Psidium guajava leaf extracts in the treatment of acute diarrhoeal disease. *J. Ethnopharmacol.* 25, 235–47. doi: 10.1016/0378-8741(89)90030-5

Lutterodt, G. D. (1992). Inhibition of microlax-induced experimental diarrhoea with narcotic-like extracts of Psidium guajava leaf in rats. *J. Ethnopharmacol.* 37, 151–157. doi: 10.1016/0378-8741(92)90073-Z

Madikizela, B., Ndhlala, A. R., Finnie, J. F., and Van Staden, J. (2012). Ethnopharmacological study of plants from Pondoland used against diarrhoea. *J. Ethnopharmacol.* 141, 61–71. doi: 10.1016/j.jep.2012.01.053

Maïkere-Faniyo, R., Van Puyvelde, L., Mutwewingabo, A., and Habiyaremye, F. X. (1989). Study of Rwandese medicinal plants used in the treatment of diarrhoea I. *J. Ethnopharmacol.* 26, 101–109. doi: 10.1016/0378-8741(89)90057-3

Mallik, B. K., Panda, T., and Padhy, R. N. (2012). Ethnoveterinary practices of aborigine tribes in Odisha, India. *Asian Pac. J. Trop. Biomed.* 2, S1520–S1525. doi: 10.1016/S2221-1691(12)60447-X

Mazumdar, S., Akter, R., and Talukder, D. (2015). Antidiabetic and antidiarrhoeal effects on ethanolic extract of Psidium guajava (L.) Bat. leaves in Wister rats. *Asian Pac. J. Trop. Biomed.* 5, 10–14. doi: 10.1016/S2221-1691(15)30163-5

McClatchey, W. (1996). The ethnopharmacopoeia of Rotuma. *J. Ethnopharmacol.* 50, 147–156. doi: 10.1016/0378-8741(95)01343-1

McCook-Russell, K. P., Nair, M. G., Facey, P. C., and Bowen-Forbes, C. S. (2012). Nutritional and nutraceutical comparison of Jamaican Psidium cattleianum (strawberry guava) and Psidium guajava (common guava) fruits. *Food Chem.* 134, 1069–1073. doi: 10.1016/j.foodchem.2012.03.018

Mhlongo, L. S., and Van Wyk, B.-E. (2019). Zulu medicinal ethnobotany: new records from the Amandawe area of KwaZulu-Natal, South Africa. *South Afr. J. Bot.* 122, 266–290. doi: 10.1016/j.sajb.2019.02.012

Mondo, J. M., Chuma, G. B., Kwalya, P. B., Balagizi, S. A., Ndjadi, S. S., Mugumaarhahama, Y., et al. (2021). Neglected and underutilized crop species in Kabare and Walungu territories, Eastern D.R. Congo: Identification, uses and socio-economic importance. *J. Agric. Food Res.* 6, 100234. doi: 10.1016/j.jafr.2021.100234

Monigatti, M., Bussmann, R. W., and Weckerle, C. S. (2013). Medicinal plant use in two Andean communities located at different altitudes in the Bolívar Province, Peru. *J. Ethnopharmacol.* 145, 450–464. doi: 10.1016/j.jep.2012.10.066

Morais-Braga, M. F. B., Carneiro, J. N. P., Machado, A. J. T., dos Santos, A. T. L., Sales, D. L., Lima, L. F., et al. (2016). Psidium guajava L., from ethnobiology to scientific evaluation: Elucidating bioactivity against pathogenic microorganisms. *J. Ethnopharmacol.* 194, 1140–1152. doi: 10.1016/j.jep.2016.11.017

Mulyoutami, E., Rismawan, R., and Joshi, L. (2009). Local knowledge and management of simpukng (forest gardens) among the Dayak people in East Kalimantan, Indonesia. *For. Ecol. Manag.* 257, 2054–2061. doi: 10.1016/j.foreco.2009.01.042

Nagata, J. M., Jew, A. R., Kimeu, J. M., Salmen, C. R., Bukusi, E. A., and Cohen, C. R. (2011). Medical pluralism on Mfangano Island: Use of medicinal plants among persons living with HIV/AIDS in Suba District, Kenya. *J. Ethnopharmacol.* 135, 501–509. doi: 10.1016/j.jep.2011.03.051

Nautiyal, S., and Goswami, M. (2022). Role of traditional ecological knowledge on field margin vegetation in sustainable development: A study in a rural-urban interface of Bengaluru. *Trees For. People* 8, 100207. doi: 10.1016/j.tfp.2022.100207

Neamsuvan, O., Phumchareon, T., Bunphan, W., and Kaosaeng, W. (2016). Plant materials for gastrointestinal diseases used in Chawang District, Nakhon Si Thammarat Province, Thailand. *J. Ethnopharmacol.* 194, 179–187. doi: 10.1016/j.jep.2016.09.001

Neamsuvan, O., Tuwaemaengae, T., Bensulong, F., Asae, A., and Mosamae, K. (2012). A survey of folk remedies for gastrointestinal tract diseases from Thailand’s three southern border provinces. *J. Ethnopharmacol.* 144, 11–21. doi: 10.1016/j.jep.2012.07.043

Neiva, V., Ribeiro, M., Nascimento, F., Cartagenes, M., Coutinho-Moraes, D., and do Amaral, F. (2014). Plant species used in giardiasis treatment: ethnopharmacology and in vitro evaluation of anti-Giardia activity. *Rev. Bras. Farmacogn.* 24, 215–224. doi: 10.1016/j.bjp.2014.04.004

Njoroge, G. N., and Bussmann, R. W. (2006). Herbal usage and informant consensus in ethnoveterinary management of cattle diseases among the Kikuyus (Central Kenya). *J. Ethnopharmacol.* 108, 332–339. doi: 10.1016/j.jep.2006.05.031

Noumi, E., and Yomi, A. (2001). Medicinal plants used for intestinal diseases in Mbalmayo Region, Central Province, Cameroon. *Fitoterapia* 72, 246–254. doi: 10.1016/S0367-326X(00)00288-4

Nunkoo, D. H., and Mahomoodally, M. F. (2012). Ethnopharmacological survey of native remedies commonly used against infectious diseases in the tropical island of Mauritius. *J. Ethnopharmacol.* 143, 548–564. doi: 10.1016/j.jep.2012.07.013

Odonne, G., Valadeau, C., Alban-Castillo, J., Stien, D., Sauvain, M., and Bourdy, G. (2013). Medical ethnobotany of the Chayahuita of the Paranapura basin (Peruvian Amazon). *J. Ethnopharmacol.* 146, 127–153. doi: 10.1016/j.jep.2012.12.014

Olajide, O. A., Awe, S. O., and Makinde, J. M. (1999). Pharmacological studies on the leaf of Psidium guajava. *Fitoterapia* 70, 25–31. doi: 10.1016/S0367-326X(98)00010-0

Olatunde, O. O., Tan, S. L. D., Shiekh, K. A., Benjakul, S., and Nirmal, N. P. (2021). Ethanolic guava leaf extracts with different chlorophyll removal processes: Anti-melanosis, antibacterial properties and the impact on qualities of Pacific white shrimp during refrigerated storage. *Food Chem.* 341, 128251. doi: 10.1016/j.foodchem.2020.128251

Ong, H. C., and Nordiana, M. (1999). Malay ethno-medico botany in Machang, Kelantan, Malaysia. *Fitoterapia* 70, 502–513. doi: 10.1016/S0367-326X(99)00077-5

Ouachinou, J. M.-A. S., Dassou, G. H., Idohou, R., Adomou, A. C., and Yédomonhan, H. (2019). National inventory and usage of plant-based medicine to treat gastrointestinal disorders with cattle in Benin (West Africa). *South Afr. J. Bot.* 122, 432–446. doi: 10.1016/j.sajb.2019.03.037

Panmei, R., Gajurel, P. R., and Singh, B. (2019). Ethnobotany of medicinal plants used by the Zeliangrong ethnic group of Manipur, northeast India. *J. Ethnopharmacol.* 235, 164–182. doi: 10.1016/j.jep.2019.02.009

Panyadee, P., Balslev, H., Wangpakapattanawong, P., and Inta, A. (2019). Medicinal plants in homegardens of four ethnic groups in Thailand. *J. Ethnopharmacol.* 239, 111927. doi: 10.1016/j.jep.2019.111927

Perera, H. D. S. M., Samarasekera, J. K. R. R., Handunnetti, S. M., and Weerasena, O. V. D. S. J. (2016). In vitro anti-inflammatory and anti-oxidant activities of Sri Lankan medicinal plants. *Ind. Crops Prod.* 94, 610–620. doi: 10.1016/j.indcrop.2016.09.009

Pesewu, G. A., Cutler, R. R., and Humber, D. P. (2008). Antibacterial activity of plants used in traditional medicines of Ghana with particular reference to MRSA. *J. Ethnopharmacol.* 116, 102–111. doi: 10.1016/j.jep.2007.11.005

Poonthananiwatkul, B., Lim, R. H. M., Howard, R. L., Pibanpaknitee, P., and Williamson, E. M. (2015). Traditional medicine use by cancer patients in Thailand. *J. Ethnopharmacol.* 168, 100–107. doi: 10.1016/j.jep.2015.03.057

Prescott, T. A. K., Kiapranis, R., and Maciver, S. K. (2012). Comparative ethnobotany and in-the-field antibacterial testing of medicinal plants used by the Bulu and inland Kaulong of Papua New Guinea. *J. Ethnopharmacol.* 139, 497–503. doi: 10.1016/j.jep.2011.09.058

Radha, P., Udhayavani, C., Nagaraj, R., and Sivaranjani, K. (2022). Documentation and quantitative analysis of the traditional knowledge on medicinal plants in Udumalpet Block, Tiruppur District, Tamil Nadu, India. *Acta Ecol. Sin.* 42, 122–142. doi: 10.1016/j.chnaes.2021.10.009

Rajoo, K. S., Lepun, P., Alan, R., Singh Karam, D., Abdu, A., Rosli, Z., et al. (2022). Ethnobotanical study of medicinal plants used by the Kenyah community of Borneo. *J. Ethnopharmacol.*, 115780. doi: 10.1016/j.jep.2022.115780

Ribeiro, R. V., Bieski, I. G. C., Balogun, S. O., and Martins, D. T. de O. (2017). Ethnobotanical study of medicinal plants used by Ribeirinhos in the North Araguaia microregion, Mato Grosso, Brazil. *J. Ethnopharmacol.* 205, 69–102. doi: 10.1016/j.jep.2017.04.023

Ricardo, L. M., Paula-Souza, J. de, Andrade, A., and Brandão, M. G. L. (2017). Plants from the Brazilian Traditional Medicine: species from the books of the Polish physician Piotr Czerniewicz (Pedro Luiz Napoleão Chernoviz, 1812–1881). *Rev. Bras. Farmacogn.* 27, 388–400. doi: 10.1016/j.bjp.2017.01.002

Riondato, I., Donno, D., Roman, A., Razafintsalama, V. E., Petit, T., Mellano, M. G., et al. (2019). First ethnobotanical inventory and phytochemical analysis of plant species used by indigenous people living in the Maromizaha forest, Madagascar. *J. Ethnopharmacol.* 232, 73–89. doi: 10.1016/j.jep.2018.12.002

Roosita, K., Kusharto, C. M., Sekiyama, M., Fachrurozi, Y., and Ohtsuka, R. (2008). Medicinal plants used by the villagers of a Sundanese community in West Java, Indonesia. *J. Ethnopharmacol.* 115, 72–81. doi: 10.1016/j.jep.2007.09.010

Roy, M., Sarkar, B. Ch., Shukla, G., Vineeta, Debnath, M. K., Nath, A. J., et al. (2022). Traditional homegardens and ethnomedicinal plants: Insights from the Indian Sub-Himalayan region. *Trees For. People* 8, 100236. doi: 10.1016/j.tfp.2022.100236

Samoisy, A. K., and Mahomoodally, F. (2016). Ethnopharmacological appraisal of culturally important medicinal plants and polyherbal formulas used against communicable diseases in Rodrigues Island. *J. Ethnopharmacol.* 194, 803–818. doi: 10.1016/j.jep.2016.10.041

Sanz-Biset, J., Campos-de-la-Cruz, J., Epiquién-Rivera, M. A., and Cañigueral, S. (2009). A first survey on the medicinal plants of the Chazuta valley (Peruvian Amazon). *J. Ethnopharmacol.* 122, 333–362. doi: 10.1016/j.jep.2008.12.009

Semenya, S., Potgieter, M., Tshisikhawe, M., Shava, S., and Maroyi, A. (2012). Medicinal utilization of exotic plants by Bapedi traditional healers to treat human ailments in Limpopo province, South Africa. *J. Ethnopharmacol.* 144, 646–655. doi: 10.1016/j.jep.2012.10.005

Semenya, S. S., and Maroyi, A. (2012). Medicinal plants used by the Bapedi traditional healers to treat diarrhoea in the Limpopo Province, South Africa. *J. Ethnopharmacol.* 144, 395–401. doi: 10.1016/j.jep.2012.09.027

Sen, S. S., Sukumaran, V., Giri, S. S., and Park, S. C. (2015). Flavonoid fraction of guava leaf extract attenuates lipopolysaccharide-induced inflammatory response via blocking of NF-κB signalling pathway in Labeo rohita macrophages. *Fish Shellfish Immunol.* 47, 85–92. doi: 10.1016/j.fsi.2015.08.031

Sharma, U. K., Pegu, S., Hazarika, D., and Das, A. (2012). Medico-religious plants used by the Hajong community of Assam, India. *J. Ethnopharmacol.* 143, 787–800. doi: 10.1016/j.jep.2012.06.053

Shittu, O., Ajayi, O., Bankole, S., and Popoola, T. (2016). Intestinal ameliorative effects of traditional Ogi-tutu, Vernonia amygdalina and Psidium guajava in mice infected with Vibrio cholera. *Afr. Health Sci.* 16, 620–628. doi: 10.4314/ahs.v16i2.33

Sivasankari, B., Anandharaj, M., and Gunasekaran, P. (2014). An ethnobotanical study of indigenous knowledge on medicinal plants used by the village peoples of Thoppampatti, Dindigul district, Tamilnadu, India. *J. Ethnopharmacol.* 153, 408–423. doi: 10.1016/j.jep.2014.02.040

Soelberg, J., Davis, O., and Jäger, A. K. (2016). Historical versus contemporary medicinal plant uses in the US Virgin Islands. *J. Ethnopharmacol.* 192, 74–89. doi: 10.1016/j.jep.2016.07.005

Srithi, K., Balslev, H., Wangpakapattanawong, P., Srisanga, P., and Trisonthi, C. (2009). Medicinal plant knowledge and its erosion among the Mien (Yao) in northern Thailand. *J. Ethnopharmacol.* 123, 335–342. doi: 10.1016/j.jep.2009.02.035

Ssegawa, P., and Kasenene, J. M. (2007). Medicinal plant diversity and uses in the Sango bay area, Southern Uganda. *J. Ethnopharmacol.* 113, 521–540. doi: 10.1016/j.jep.2007.07.014

Sule, A., Olalemi, A., and Ogundare, A. (2022). Efficacy of leaf extracts of Psidium guajava (L.) on enteric bacterial isolates from faecally impacted groundwater. *Water Pract. Technol.* 17, 167–174. doi: 10.2166/wpt.2021.113

Suroowan, S., and Mahomoodally, F. (2013). Complementary and alternative medicine use among Mauritian women. *Complement. Ther. Clin. Pract.* 19, 36–43. doi: 10.1016/j.ctcp.2012.07.002

Tabuti, J. R. S., Lye, K. A., and Dhillion, S. S. (2003). Traditional herbal drugs of Bulamogi, Uganda: plants, use and administration. *J. Ethnopharmacol.* 88, 19–44. doi: 10.1016/S0378-8741(03)00161-2

Tangjang, S., Namsa, N. D., Aran, C., and Litin, A. (2011). An ethnobotanical survey of medicinal plants in the Eastern Himalayan zone of Arunachal Pradesh, India. *J. Ethnopharmacol.* 134, 18–25. doi: 10.1016/j.jep.2010.11.053

Tangjitman, K., Wongsawad, C., Winijchaiyanan, P., Sukkho, T., Kamwong, K., Pongamornkul, W., et al. (2013). Traditional knowledge on medicinal plant of the Karen in northern Thailand: A comparative study. *J. Ethnopharmacol.* 150, 232–243. doi: 10.1016/j.jep.2013.08.037

Tareau, M. A., Palisse, M., and Odonne, G. (2017). As vivid as a weed… Medicinal and cosmetic plant uses amongst the urban youth in French Guiana. *J. Ethnopharmacol.* 203, 200–213. doi: 10.1016/j.jep.2017.03.031

Tchetan, E., Olounlade, A. P., Houehanou, T. D., Azando, E. V. B., Kaneho, J. A., Houinato, M. R. B., et al. (2021). Ethnoveterinary knowledge of sheep and goat farmers in Benin (West Africa): effect of socioeconomic and environmental factors. *Heliyon* 7, e07656. doi: 10.1016/j.heliyon.2021.e07656

Tene, V., Malagón, O., Finzi, P. V., Vidari, G., Armijos, C., and Zaragoza, T. (2007). An ethnobotanical survey of medicinal plants used in Loja and Zamora-Chinchipe, Ecuador. *J. Ethnopharmacol.* 111, 63–81. doi: 10.1016/j.jep.2006.10.032

Tetali, P., Waghchaure, C., Daswani, P. G., Antia, N. H., and Birdi, T. J. (2009). Ethnobotanical survey of antidiarrhoeal plants of Parinche valley, Pune district, Maharashtra, India. *J. Ethnopharmacol.* 123, 229–236. doi: 10.1016/j.jep.2009.03.013

Thangliankhup, K., Lalfakawma, Gouda, S., and Khomdram, S. D. (2022). Ethnomedicinal plants of Kuki-Chin tribes in Kaihlam wildlife sanctuary of Manipur, India. *Acta Ecol. Sin.* doi: 10.1016/j.chnaes.2022.07.011

Thomas, E., Semo, L., Morales, M., Noza, Z., Nuñez, H., Cayuba, A., et al. (2011). Ethnomedicinal practices and medicinal plant knowledge of the Yuracarés and Trinitarios from Indigenous Territory and National Park Isiboro-Sécure, Bolivian Amazon. *J. Ethnopharmacol.* 133, 153–163. doi: 10.1016/j.jep.2010.09.017

Tona, L., Kambu, K., Mesia, K., Cimanga, K., Apers, S., De Bruyne, T., et al. (1999). Biological screening of traditional preparations from some medicinal plants used as antidiarrhoeal in Kinshasa, Congo. *Phytomedicine* 6, 59–66. doi: 10.1016/S0944-7113(99)80036-1

Tona, L., Kambu, K., Ngimbi, N., Cimanga, K., and Vlietinck, A. J. (1998). Antiamoebic and phytochemical screening of some Congolese medicinal plants. *J. Ethnopharmacol.* 61, 57–65. doi: 10.1016/S0378-8741(98)00015-4

Tona, L., Kambu, K., Ngimbi, N., Mesia, K., Penge, O., Lusakibanza, M., et al. (2000). Antiamoebic and spasmolytic activities of extracts from some antidiarrhoeal traditional preparations used in Kinshasa, Congo. *Phytomedicine* 7, 31–38. doi: 10.1016/S0944-7113(00)80019-7

Tribess, B., Pintarelli, G. M., Bini, L. A., Camargo, A., Funez, L. A., de Gasper, A. L., et al. (2015). Ethnobotanical study of plants used for therapeutic purposes in the Atlantic Forest region, Southern Brazil. *J. Ethnopharmacol.* 164, 136–146. doi: 10.1016/j.jep.2015.02.005

Tuler, A. C., and da Silva, N. C. B. (2014). Women’s ethnomedicinal knowledge in the rural community of São José da Figueira, Durandé, Minas Gerais, Brazil. *Rev. Bras. Farmacogn.* 24, 159–170. doi: 10.1016/j.bjp.2014.03.004

Upadhyay, B., Parveen, Dhaker, A. K., and Kumar, A. (2010). Ethnomedicinal and ethnopharmaco-statistical studies of Eastern Rajasthan, India. *J. Ethnopharmacol.* 129, 64–86. doi: 10.1016/j.jep.2010.02.026

Valadeau, C., Castillo, J. A., Sauvain, M., Lores, A. F., and Bourdy, G. (2010). The rainbow hurts my skin: Medicinal concepts and plants uses among the Yanesha (Amuesha), an Amazonian Peruvian ethnic group. *J. Ethnopharmacol.* 127, 175–192. doi: 10.1016/j.jep.2009.09.024

van Vuuren, S. F., Nkwanyana, M. N., and de Wet, H. (2015a). Antimicrobial evaluation of plants used for the treatment of diarrhoea in a rural community in northern Maputaland, KwaZulu-Natal, South Africa. *BMC Complement. Altern. Med.* 15, 53. doi: 10.1186/s12906-015-0570-2

van Vuuren, S., Nkwanyana, M., and de Wet, H. (2015b). Antimicrobial evaluation of plants used for the treatment of diarrhoea in a rural community in northern Maputaland, KwaZulu-Natal, South Africa. *BMC Complement. Altern. Med.* 15. doi: 10.1186/s12906-015-0570-2

Vieira, R. H. S. dos F., Rodrigues, D. dos P., Gonçalves, F. A., Menezes, F. G. R. de, Aragão, J. S., and Sousa, O. V. (2001). Microbicidal effect of medicinal plant extracts (Psidium guajava Linn. and Carica papaya Linn.) upon bacteria isolated from fish muscle and known to induce diarrhea in children. *Rev. Inst. Med. Trop. Sao Paulo* 43, 145–148. doi: 10.1590/S0036-46652001000300005

Vijayakumar, S., Morvin Yabesh, J. E., Prabhu, S., Manikandan, R., and Muralidharan, B. (2015). Quantitative ethnomedicinal study of plants used in the Nelliyampathy hills of Kerala, India. *J. Ethnopharmacol.* 161, 238–254. doi: 10.1016/j.jep.2014.12.006

Voravuthikunchai, S., Lortheeranuwat, A., Jeeju, W., Sririrak, T., Phongpaichit, S., and Supawita, T. (2004). Effective medicinal plants against enterohaemorrhagic Escherichia coli O157:H7. *J. Ethnopharmacol.* 94, 49–54. doi: 10.1016/j.jep.2004.03.036

Wang, D., Zhou, L., Zhou, H., Hu, H., and Hou, G. (2021). Chemical composition and protective effect of guava (Psidium guajava L.) leaf extract on piglet intestines. *J. Sci. Food Agric.* 101, 2767–2778. doi: 10.1002/jsfa.10904

Yazbek, P. B., Matta, P., Passero, L. F., Santos, G. dos, Braga, S., Assunção, L., et al. (2019). Plants utilized as medicines by residents of Quilombo da Fazenda, Núcleo Picinguaba, Ubatuba, São Paulo, Brazil: A participatory survey. *J. Ethnopharmacol.* 244, 112123. doi: 10.1016/j.jep.2019.112123

Zamora-Martinez, M. C., and de Pascual Pola, C. N. (1992). Medicinal plants used in some rural populations of Oaxaca, Puebla and Veracruz, Mexico. *J. Ethnopharmacol.* 35, 229–257. doi: 10.1016/0378-8741(92)90021-I

Zhang, W., Wang, J., Chen, Y., Zheng, H., Xie, B., and Sun, Z. (2018). Flavonoid compounds and antibacterial mechanisms of different parts of white guava (Psidium guajava L. cv. Pearl). *Nat. Prod. Res.* 34, 1621–1625. doi: 10.1080/14786419.2018.1522313

Zheng, X., Wei, J., Sun, W., Li, R., Liu, S., and Dai, H. (2013). Ethnobotanical study on medicinal plants around Limu Mountains of Hainan Island, China. *J. Ethnopharmacol.* 148, 964–974. doi: 10.1016/j.jep.2013.05.051
